# Deep generative neural network for accurate drug response imputation

**DOI:** 10.1038/s41467-021-21997-5

**Published:** 2021-03-19

**Authors:** Peilin Jia, Ruifeng Hu, Guangsheng Pei, Yulin Dai, Yin-Ying Wang, Zhongming Zhao

**Affiliations:** 1 Center for Precision Health, School of Biomedical Informatics, The University of Texas Health Science Center at Houston, Houston, TX USA; 2 Human Genetics Center, School of Public Health, The University of Texas Health Science Center at Houston, Houston, TX USA; 3 MD Anderson Cancer Center UTHealth Graduate School of Biomedical Sciences, Houston, TX USA; 4 Department of Biomedical Informatics, Vanderbilt University Medical Center, Nashville, TN USA

**Keywords:** Cancer genomics, Machine learning, Drug development

## Abstract

Drug response differs substantially in cancer patients due to inter- and intra-tumor heterogeneity. Particularly, transcriptome context, especially tumor microenvironment, has been shown playing a significant role in shaping the actual treatment outcome. In this study, we develop a deep variational autoencoder (VAE) model to compress thousands of genes into latent vectors in a low-dimensional space. We then demonstrate that these encoded vectors could accurately impute drug response, outperform standard signature-gene based approaches, and appropriately control the overfitting problem. We apply rigorous quality assessment and validation, including assessing the impact of cell line lineage, cross-validation, cross-panel evaluation, and application in independent clinical data sets, to warrant the accuracy of the imputed drug response in both cell lines and cancer samples. Specifically, the expression-regulated component (EReX) of the observed drug response achieves high correlation across panels. Using the well-trained models, we impute drug response of The Cancer Genome Atlas data and investigate the features and signatures associated with the imputed drug response, including cell line origins, somatic mutations and tumor mutation burdens, tumor microenvironment, and confounding factors. In summary, our deep learning method and the results are useful for the study of signatures and markers of drug response.

## Abstract (web summary)

Drug response in cancer patients vary dramatically due to inter- and intra-tumor heterogeneity and transcriptome context plays a significant role in shaping the actual treatment outcome. Here, the authors develop a deep variational autoencoder model to compress gene signatures into latent vectors and accurately impute drug response.

## Introduction

A systematic investigation of the association between cancer genomics data and their therapeutic possibilities can greatly aid the translation of theoretical tumor biology. Several large-scale pharmacogenomics projects have been conducted at the cell line level with many conditions, substantially advancing our knowledge of drug response. These projects include the Cancer Cell Line Encyclopedia (CCLE)^1^, Genomics of Drug Sensitivity in Cancer (GDSC)^2^, and The Library of Integrated Network-Based Cellular Signatures (LINCS)^3^, among others. Although such data were much valuable, it was based on preclinical features and suffered from limitations on small sample size and high heterogeneity. On the other hand, The Cancer Genome Atlas (TCGA) project^4^ has released comprehensive multi-omics datasets in 33 cancer types/subtypes. Unfortunately, the pharmacogenomics information is generally unavailable in these cancer datasets, leading to a strong gap between the cancer genomic studies and therapeutic responses at the pan-cancer level. To fill in this gap, several studies have attempted to infer drug response for TCGA data by developing various prediction models, including statistical models that link gene expression to drug response^5^ and lncRNA-drug prediction models using Elastic Net regression^6^.

In silico prediction (also called imputation) of drug response currently remains challenging due to several factors. First, the selection of targeted therapies on specific oncogenes in clinical practice has been limited to only a few specific mutations^7–9^, e.g., BRAF V600E mutation^10,11^, or a few signature genes^12^. However, mutational status alone is insufficient to determine the oncogenic state of a given cancer sample or recommend the appropriate therapeutic choice. This issue becomes worse for those drugs with general cytotoxicity or having multiple molecular targets. Second, cancer heterogeneity is a well-known factor contributing to the great variability in drug response in cancer patients. For instance, in lung cancer with *KRAS* mutations, the epithelial–mesenchymal transition (EMT) program has been shown to underlie the variability in response to *KRAS* inhibitors^13^. The transcriptional context may play deterministic roles in shaping the response to inhibitors and should be included in the prediction model. Third, the measured drug response data from cell lines are typically noisy and sometimes biased for certain drugs. Notably, cancer cell lines have their own lineage origins, but many existing methods train their models without distinguishing specific types of cell lines. For example, for the drugs whose targets are predominantly mutated in solid tumors, training a prediction model using both hematopoietic cell lines and cell lines with solid tumor origins may reduce the power, when compared to the models trained using only the latter type of cell lines. There are many factors that may influence the accuracy of prediction models for drug response. It is important to use the input from a number of informative molecules with potential contributing roles rather than a few targeted genes.

In this work, we developed a deep-learning approach for drug response prediction based on deep regenerative models. By applying the variational autoencoder (VAE) framework, we generated representative models for >1000 cell lines using their baseline expression profile and trained prediction models for drug response based on the latent representation of the baseline expression profiles. Rigorous quality assessment and validation were implemented, including cross-validation, multiple replications, and cross-panel evaluation. Because the models were built on a baseline gene expression profile, they are widely applicable. We thus applied the models to large cohorts such as the TCGA pan-cancer samples with gene expression profiles and conducted association tests to infer various drug–molecule associations, including somatic mutations, copy number variants (CNVs), gene expression, and drug-pathway associations. The results revealed the landscape of molecular features in association with drug response in 33 cancer types that were beyond the information obtained by using the typical cell line models.

## Results

**Fig. 1:**
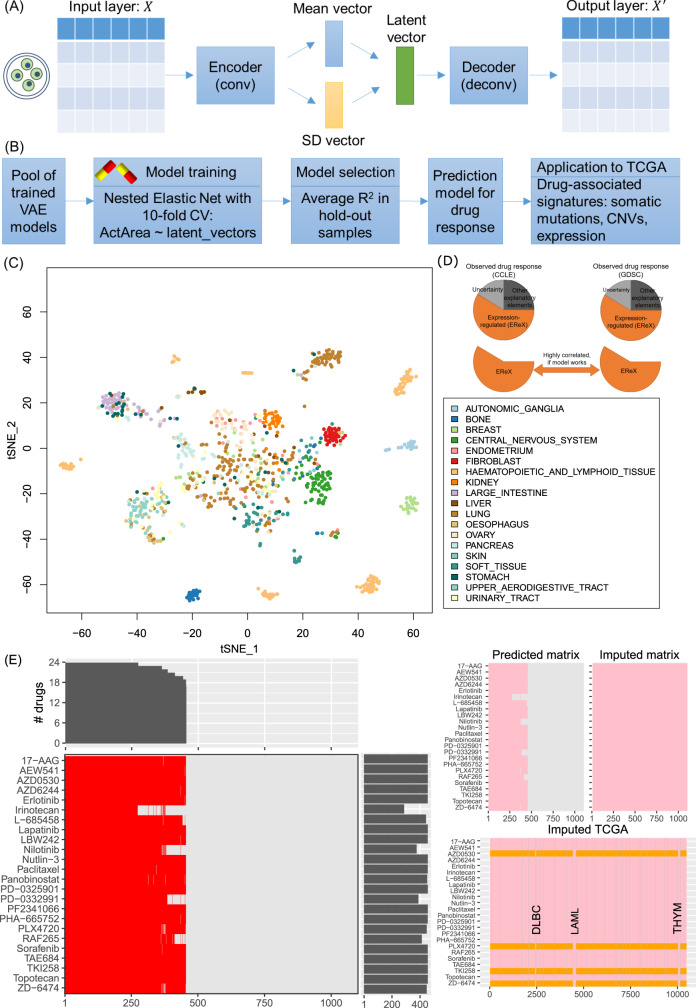
Illustration of the workflow. **A** The variational autoencoder (VAE) models. Conv convolution, SD standard deviation. **B** The pipeline to train regression models for drug response. CV cross-validation. **C** t-SNE plot showing the distribution of cell lines with origins using their transcriptome profiles. **D** Decomposition of the observed (measured) drug response. EReX expression-regulated component. **E** Illustration of the cell lines and cancer types with measured and imputed drug response using CCLE. *x* axis indicates cell line index or TCGA cancer sample index. The red bars indicated the cell lines (set 1) that had measured drug response for each of the 24 compounds. The predicted matrix indicated the cell lines with imputed drug response (set 2, pink). The imputed matrix indicated all cell lines had imputed drug response (set 3, pink). For the TCGA samples, pink bars indicated that the samples had predicted drug response based on the A-model and the orange bards indicated that the samples had predicted drug response based on the S-model. The vertical gray lines split the samples into 33 cancer types. The order of these cancer types is as below: ACC adrenocortical carcinoma, BLCA bladder urothelial carcinoma, BRCA breast invasive carcinoma, CESC cervical and endocervical cancers, CHOL cholangiocarcinoma, COAD colon adenocarcinoma, DLBC lymphoid neoplasm diffuse large B-cell lymphoma, ESCA esophageal carcinoma, GBM glioblastoma multiforme, HNSC head and neck squamous cell carcinoma, KICH kidney chromophobe, KIRC kidney renal clear cell carcinoma, KIRP kidney renal papillary cell carcinoma, LAML acute myeloid leukemia, LGG brain lower grade glioma, LIHC liver hepatocellular carcinoma, LUAD lung adenocarcinoma,LUSC lung squamous cell carcinoma, MESO mesothelioma, OV ovarian serous cystadenocarcinoma, PAAD pancreatic adenocarcinoma, PCPG pheochromocytoma and paraganglioma, PRAD prostate adenocarcinoma, READ rectum adenocarcinoma, SARC sarcoma, SKCM skin cutaneous melanoma, STAD stomach adenocarcinoma, TGCT testicular germ cell tumors, THCA thyroid carcinoma, THYM thymoma, UCEC uterine corpus endometrial carcinoma, UCS uterine carcinosarcoma, UVM uveal melanoma. LAML and THCA had all their response predicted based on the A-model (pink).

Our imputation pipeline and analysis consist of three major components (Fig. 1): a deep regenerative model for the representation of the baseline expression of cell lines used in CCLE and GDSC projects (Fig. 1A, C), a regression model to impute drug response using the measured IC_50_ data from CCLE and GDSC (Fig. 1B), and validations and applications of our approach to TCGA and other clinical datasets (Fig. 1E). First, we used the baseline gene expression data to build the VAE models, resulting in a compressed representation of the samples in the low-dimensional latent space, which consisted of latent vectors. Second, we utilized the latent vectors as the exposure variables and trained regression models to predict drug response using the observed data from CCLE and GDSC. These predictive models were achieved by employing an Elastic Net strategy (VAE model followed by Elastic Net, or VAEN) and we did it for each measured compound. Conceptually, the observed (measured) drug response can be decomposed into the expression-regulated component (EReX, following the naming system of a previous work^14^), the component by other explanatory elements (e.g., genetics variants, methylation, microRNA, and lncRNA), and the component due to uncertainty (e.g., batch effect and experiment conditions) (Fig. 1D). The component that VAEN and its peers can estimate is EReX, and theoretically EReX remains the same for each drug regardless of the measurement platforms or prediction methods.

### VAE model resembles cell line lineages

The original cell lines from the CCLE and GDSC projects represented a wide spectrum of cell line lineages, such as the epithelial, mesenchymal, and hematopoietic background. In our VAE model, we included 1100 cell lines from 19 tissues of origin, with each tissue being required to have at least 20 cell lines. We also filtered for genes that were most variably expressed across cell lines to build the VAE models (*n* = 6163). A parameter sweep was conducted to determine several key parameters that would affect the resultant models, including the latent size, learning rate, batch effect, and the number of epochs. We used the loss between the original data and reconstructed data to evaluate the fitted model. As shown in Supplementary Fig. 1, we selected a latent size 100, learning rate 0.0005, batch size 100, and epoch times 100 to build our models, as the loss by these parameter settings was among the minimum. The resultant latent vectors were on a dimension of 100 latent vectors × 1100 cell lines. We applied t-Distributed Stochastic Neighbor Embedding (t-SNE)^15^ to represent the data and observed major clusters organized by cell lineages. As shown in Fig. 1C, cell lines of hematopoietic and lymphoid tissue origin, fibroblast, skin, stomach, and breast could form distinct clusters. However, we did not observe a cluster significantly associated with any drug.

### VAEN prediction models

The VAE model itself was fitted to achieve a compressed representation of the input data but it was not tailored toward a better prediction of drug response. Unlike the traditional principal component analysis (PCA), the compressed representation from VAE is not unique. With the same input matrix, there could be numerous matrices to faithfully represent the input data, each of which might be slightly different from others and individually serve better to one or some drugs than the other drugs. Hence, we generated 100 VAE models to build a pool for the following drug response imputation. For each drug, we applied an Elastic Net (EN) strategy using the latent vectors from each VAE model to train the VAEN prediction models. We used a fixed alpha of 0.5 and conducted fivefold cross-validation to select lambda. We then evaluated the selected latent vectors (i.e., those with nonzero coefficients from the EN model) using tenfold cross-validation by a standard multivariate linear regression. The average coefficient of determination *R*^2^ in the holdout samples was used to measure the model efficiency. Among the 100 VAE models (i.e., 100 latent matrices), the one with the highest average holdout *R*^2^ was selected for each drug in the following analyses.

**Fig. 2:**
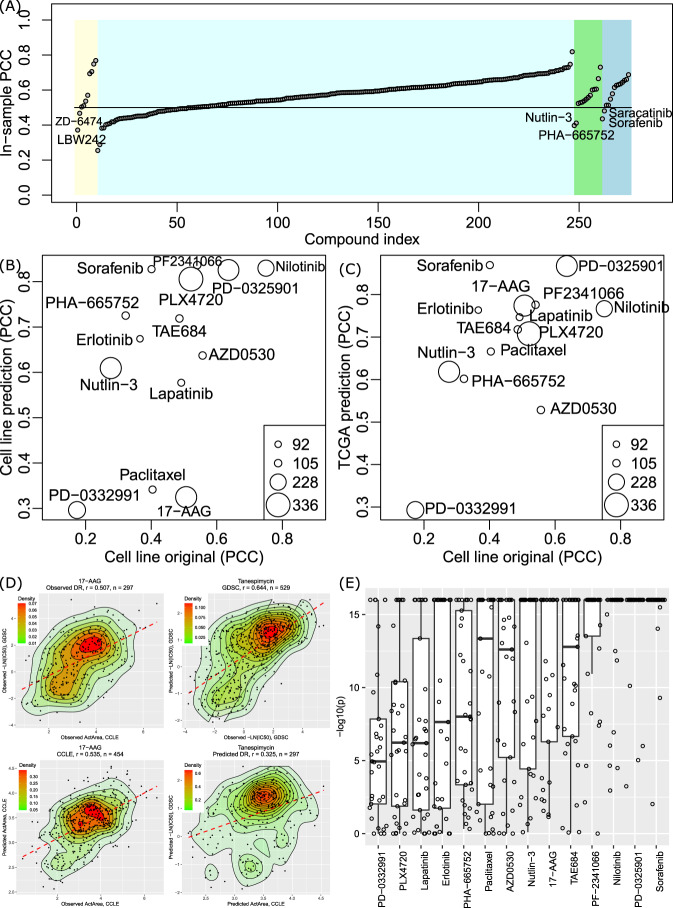
Evaluation of the model efficiency. **A** Distribution of in-sample Pearson correlation coefficient (PCC) for all drugs. The four panels presented the performance of drugs that are unique to CCLE (*n* = 10, yellow panel), unique to GDSC (*n* = 237, light-blue panel), shared drugs in CCLE (*n* = 14, green panel), and shared drugs in GDSC (*n* = 14, blue panel). **B** Evaluation of the 14 drugs shared between the CCLE and GDSC panels. *x* axis: PCC values of the observed drug response between CCLE and GDSC. *y* axis: PCC of the predicted drug response between CCLE and GDSC (using set 2, see main text). **C** Evaluation of the 14 shared drugs using the TCGA cancer data. *x* axis: PCC of the observed drug response between CCLE and GDSC. *y* axis: PCC of the drug response of TCGA samples (*n* = 10,459) predicted using the CCLE models and the GDSC models. **D** Demonstration of the observed and predicted drug response using an example of a shared compound 17-AAG (also called tanespimycin in GDSC). DR drug response, *r* Pearson correlation coefficient. **E** Evaluation of the predicted drug response in TCGA using the CCLE models and GDSC models. For each of the 14 shared drugs (*x* axis), we calculated the PCC and the *P* value of the predicted drug response using CCLE data and GDSC data in each of the 33 cancer types. The *P* value corresponds to the significance levels of correlations. *y* axis shows the –log10(*P*) of the PCC in 33 cancer types. On *x* axis, compounds were ordered by the average –log10(*P*) of the 33 cancer types. Each box shows the interquartile range (IQR between Q1 and Q3) for the corresponding set. The central mark (horizontal line) shows the median. The upper whisker extends from the hinge to the largest value no further than Q3 + 1.5 × IQR and the lower whisker extends from the hinge to the smallest value at most Q1−1.5 × IQR. For each box, all 33 values are shown as dots.

We tested several strategies to preprocess the original gene expression data before inputting them into the VAE models. Specifically, we tested (1) z-score normalization of each feature across samples and shrinking each feature to the [0,1] range (hereafter shortened as Z01); (2) z-score normalization of all genes for each sample (ZS); and (3) rank-based inverse normal transformation (Rank). We also tested using the sigmoid activation function or the ReLU activation function in VAE, resulting in six types of VAE models. We applied tSNE to visualize the compressed latent matrices. As shown in Supplementary Fig. 2, all six types of VAE models could resemble the original cell lineage groups. For each type of VAE compression, we then trained Elastic Net regression models to predict drug response. Because we generated 100 VAENs for each type of VAE compression and each drug, we next used the top ten VAENs for each drug with the highest average holdout *R*^2^ to evaluate the model stability. As shown in Supplementary Table 1, we found that VAE models using the sigmoid activation had a relatively high correlation, indicating the predictions were stable and robust. We also found that Rank and ZS-based normalization generated high correlation than Z01-based normalization. Consequently, we used the rank-based transformation and the sigmoid activation for the VAE part. Using the final VAEN models, the self-prediction accuracy for 24 CCLE compounds, measured by Pearson correlation coefficient (PCC), ranged between 0.38 (LBW242) and 0.77 (Irinotecan). For the 251 GDSC compounds, the self-prediction accuracy ranged between 0.26 (Avagacestat) and 0.82 (AZ628), with 203/251 (80.88%) compounds having PCC >0.5 (Fig. 2A).

We next used the 14 drugs measured by both CCLE and GDSC to evaluate the prediction results. We found that the correlation between CCLE IC_50_ and GDSC IC_50_ (PCC ranged between 0.10 in erlotinib and 0.58 in PF2341066) was not as strong as that between CCLE ActArea and GDSC LN_IC_50_ (PCC ranged between 0.17 in PD-0332991 and 0.75 in nilotinib). Therefore, we used CCLE ActArea to represent the drug response. In the comparison of the predicted drug response among the cell lines, we found a positive correlation between the observed and predicted drug response (Fig. 2B). When a drug showed a high consistent drug response between CCLE and GDSC (e.g., PCC (nilotinib) = 0.75 between the observed drug response in CCLE and in GDSC among the 227 cell lines), it also displayed a trend toward a high consistency in the predicted data (PCC (nilotinib) = 0.8 for predicted drug response). Notably, for each drug, the VAE models selected for response imputation differed in the CCLE prediction model and in the GDSC prediction model. Because different VAE models are projected in different spaces with inconsistent conformation, it is mathematically challenging to obtain a highly correlated prediction. The results in Fig. 2B indicated that our models are reproducible across study panels. Strikingly, the predicted response to each drug using the CCLE model and the GDSC model had an even higher correlation than the observed response. As aforementioned (Fig. 1D), EReX is the component that can be explained by the transcriptome. Thus, the results in Fig. 2B indicated that our models based on the low-dimensional representation of gene expression achieved a high performance to estimate EReX. A similar positive trend was observed when we applied the models to TCGA data. The drugs having high consistency in the original CCLE and GDSC projects tended to have high consistency among the predicted drug response (using the models trained by CCLE and GDSC, respectively) in cancer samples (Fig. 2C). An example of such correlations (CCLE name: 17-AAG; GDSC name: tanespimycin) is shown in Fig. 2D, while results for all drugs are provided in Supplementary Fig. 3. Specifically, when we compared the predicted drug response in each cancer type using the fitted CCLE and GDSC models, we found 50.65% (234/462) of cancer–drug pairs had a correlation >0.5. All drugs showed a significant correlation (*P* < 0.05/462 = 1.08 × 10^−4^ following the Bonferroni method) in ≥20 cancer types (Fig. 2E). The drugs that had low consistency in TCGA, such as AZD0530, erlotinib, lapatinib, and PD-0332991, were among those having the weakest consistency in the originally observed drug response.

To compare with standard methods, we also used the raw expression of the 6163 genes, instead of VAE compression, to train prediction models following the same Elastic Net strategy (hereafter named the gene + EN model). Similar strategies have been reported previously^5,16^, representing a generalized signature-based method. In addition, we employed the strategy to use the canonical PCA to compress genes (i.e., linear compression) followed by the same Elastic Net-based regression models (named PCA + EN). As shown in Supplementary Fig. 4, although the in-sample PCC was higher for both PCA + EN and gene + EN than those from our VAEN models, the cross-panel validation showed much weaker correlations in the TCGA samples. This indicated that the gene + EN model suffered from severe overfitting rates. The PCA + EN models showed better cross-panel correlations but they were still worse than VAEN models for most drugs.

### Imputed drug response in CCLE cell lines and TCGA cancer samples

As aforementioned, the cell lines in the VAE models represented groups of epithelial, mesenchymal, and hematopoietic origins. We found that for some drugs, e.g., BRAF inhibitors, cell lines with solid tumor origin showed different responses from hematopoietic cell lines. Therefore, for each drug, we trained two models, one using all cell lines (named A-model) and the other using all but hematopoietic cell lines (named S-model, S denotes solid tumor). Among the 24 CCLE drugs, we found four drugs (AZD0530, RAF265, TKI258, and ZD-6474) showed improved average holdout *R*^2^ when they were trained by using solid cell lines only, while PLX4720 received both increased self PCC and average holdout *R*^2^ (Supplementary Fig. 5). Accordingly, when imputing drug response in TCGA samples, we examined both models for these drugs in 30 out of the 33 cancer types (excluding three immune-related cancer: LAML, DLBC, and THYM) (Fig. 1E). The full names of all cancer types are available in Fig. 1.

**Fig. 3:**
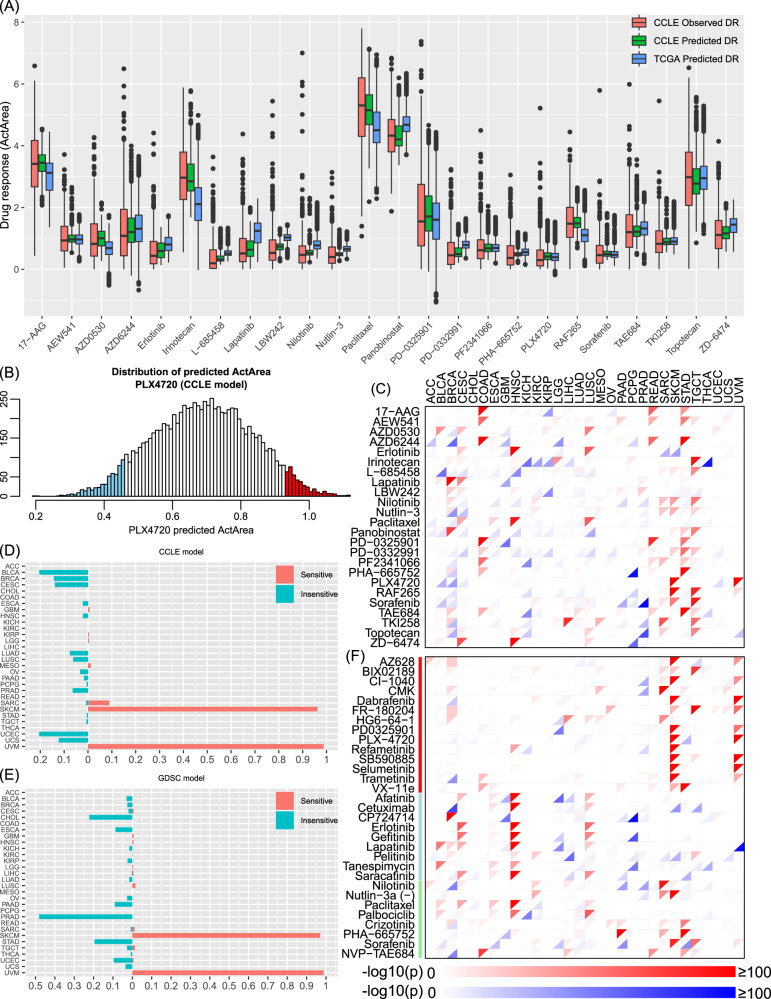
Distribution of predicted drug response across 33 cancer types. **A** Distribution of the observed and predicted drug response in CCLE. For each drug, the sample size for CCLE observed drug response (DR) and CCLE predicted DR is the same: *n* (17-AAG) = 454, *n* (AEW541) = 454, *n* (AZD0530) = 455, *n* (AZD6244) = 454, *n* (erlotinib) = 454, *n* (irinotecan) = 286, *n* (L-685458) = 442, *n* (lapatinib) = 455, *n* (LBW242) = 454, *n* (nilotinib) = 375, *n* (Nutlin-3) = 455, *n* (paclitaxel) = 454, *n* (panobinostat) = 451, *n* (PD-0325901) = 455, *n* (PD-0332991) = 388, *n* (PF2341066) = 455, *n* (PHA665752) = 454, *n* (PLX4720) = 447, *n* (RAF265) = 412, *n* (sorafenib) = 454, *n* (TAE684) = 455, *n* (TKI258) = 455, *n* (topotecan) = 455, *n* (ZD-6474) = 447. The number of samples for TCGA predicted DR is *n* = 10,459 for all drugs. Each box shows the interquartile range (IQR between Q1 and Q3) for the corresponding set. The central mark (horizontal line) shows the median and the whiskers show the rest of the distribution based on IQR (Q1−1.5 × IQR, Q3 + 1.5 × IQR). Data outside of this range are considered outliers and represented by dark dots. **B** Definition of sensitive and insensitive samples for each drug, using PLX4720 as an example. The *x* axis is the predicted drug response, and the *y* axis is the number of samples. **C** Enrichment test results of the sensitive or insensitive samples in each of the 30 cancer types (excluding three immune-related cancer types: DLBC, LAML, and THYM) using the CCLE drugs. For each cell, the top-left triangle shows the sensitive trend (in red), and the bottom right triangle shows the insensitive trend (in blue), with the color proportional to the *P* value (the legend is the same as in F and is shown at the bottom of **F**). In each cell, a *P* value for the enrichment of sensitive samples was obtained by a Fisher’s exact test by building a 2 × 2 table using two categorical variables: variable 1: whether a sample belongs to the corresponding cancer type and variable 2: whether a sample is sensitive to the corresponding drug (defined as the top 5% samples of all cancer types ordered by decreasing response to the corresponding drug). The *P* value is shown on the top-left corner if it is <0.05 (nominal significant threshold). Similarly, a *P* value for the enrichment of insensitive samples was also obtained by a Fisher’s exact test by building a 2 × 2 table using the following categorical variables: variable 1: whether a sample belongs to the corresponding cancer type and variable 2: whether a sample is insensitive to the corresponding drug (defined as the bottom 5% samples of all cancer types ordered by decreasing response to the corresponding drug). The *P* value is shown on the right-bottom corner if it is <0.05. **D**, **E** Demonstration of the proportion of sensitive samples (on the right, red) and insensitive samples (on the left, blue) in response to each drug (PLX4720 as the example on the plot) using the CCLE samples (**D**) and the GDSC samples (**E**). In the back-to-back plots in both **D** and **E**, the x axis is the proportion of the samples being sensitive (the right part) or insensitive (the left part). **F** Enrichment test results of the sensitive or insensitive samples in each of the 30 cancer types (excluding three immune-related cancer types: DLBC, LAML, and THYM) using representative GDSC drugs. The *P* values for sensitive samples and insensitive samples were similarly calculated as in (**C**) for CCLE drugs. Due to space limitation, we showed only those drugs that target on the ERK MAPK signaling pathway and EGFR signaling pathway. Drugs labeled by the vertical red bar were annotated to the ERK MAPK signaling pathway; drugs labeled by the vertical cyan bar annotated to the EGFR signaling pathway; drugs labeled by the vertical green bar included the remaining drugs of the 14 shared drugs between CCLE and GDSC after excluding those already being labeled in the ERK/MAPK or EGFR signaling pathways.

We applied our well-trained and evaluated VAEN models to all CCLE cell lines (*n* = 1100) and TCGA samples (33 cancer types, *n* = 10,459). We predicted the drug response for each sample for each of the 24 CCLE compounds and each of the 251 GDSC compounds. Here, we used CCLE to demonstrate the model performance. There were three drug-response profile sets for each compound: the observed response in a limited number of cell lines (~500, set 1, red bars in Fig. 1E), the predicted response in the same cell lines as in set 1 (set 2, pink bars with the same occupancy as the red bars in Fig. 1E), and the imputed response in all cell lines (set 3, *n* = 1100, pink bars spanning all cell lines, Fig. 1E). As shown in Fig. 3A, the predicted drug response is similar to the original data. Some compounds, such as 17-AAG, irinotecan, paclitaxel, PD-0325901, and topotecan, had a relatively wider range of response, whereas other compounds (e.g., AEW541, erlotinib, Nutlin-3, and PLX4720) had a narrow distribution. Associations that were previously reported between genomic signatures and drug response using the observed data (set 1) were well-reserved in the predicted drug response in both sets 2 and 3. For example, *BRAF* was significantly sensitive to two BRAF inhibitors and two MEK inhibitors in all three drug-response profiling sets: AZD6244 (*P*(set 1) = 9.81 × 10^−9^, *P*(set 2) = 6.88 × 10^−10^, *P*(set 3) = 1.08 × 10^−14^), PD-0325901 (*P*(set 1) = 2.41 × 10^−7^, *P*(set 2) = 2.61 × 10^−8^, *P*(set 3) = 2.07 × 10^−16^), PLX4720 (*P*(set 1) = 8.55 × 10^−6^, *P*(set 2) = 1.36 × 10^−8^, *P*(set 3) = 1.08 × 10^−11^), and RAF265 (*P*(set 1) = 6.00 × 10^−5^, *P*(set 2) = 3.82 × 10^−6^, *P*(set 3) = 1.21 × 10^−4^) (Supplementary Fig. 6). A previously reported interaction effect between *BRAF* mutations and *EGFR* expression^17^ was also replicated. We fitted regression functions following **Y**_response_ ~ **X**_*BRAF*_ + **X**_*EGFR*_ + **X**_*BRAF*_ × **X**_*EGFR*_, where **X**_*BRAF*_ indicated whether a sample harbored a *BRAF* mutation and **X**_*EGFR*_ was a factor with three levels indicating group information of a sample defined by *EGFR* gene expression (the lower quarter (Q25), the middle half (Q25-75), and the higher quarter of samples (Q75) ordered by increasing *EGFR* expression). We found an increased *EGFR* expression led to a reduced response to BRAF inhibitors in *BRAF* mutant samples but not in *BRAF* wild-type samples, consistent with previous studies that *EGFR* activation enhances resistance to BRAF inhibitors^18^. Such a conditional association was confirmed in all three sets of drug responses to all four compounds with which *BRAF* showed association (Supplementary Fig. 7).

We defined the top 5% TCGA samples that had the strongest predicted drug response using the CCLE model as the sensitive group and the 5% samples that had the weakest predicted drug response as the insensitive group (Fig. 3B). Of note, in CCLE, we used ActArea as the measurement of drug response. ActArea had a negative relationship with IC_50_ (i.e., a low IC_50_ means a high ActArea and high sensitivity). In GDSC, we used -LN(IC_50_) as the measurement of drug response, and thus, the predicted values had the same trend as ActArea, i.e., a high predicted value indicates a high sensitivity. We then conducted an enrichment analysis of the samples from each cancer type in the sensitive or the insensitive groups (Fisher’s exact test). As shown in Fig. 3C, enrichment patterns with both sensitive and insensitive groups were observed for each cancer type. For example, HNSC samples were enriched in the erlotinib-sensitive group, COAD in AZD6244 and PD-0325901, and SKCM in PLX4720 and RAF265 groups. In GDSC, we observed that SKCM was enriched with sensitive samples to multiple MAPK inhibitors, including dabrafenib, PD-0325901, PLX4720, refametinib, selumetinib, and trametinib. Due to space limitation, we only showed inhibitors to ERK MAPK signaling and EGFR signaling, and the 14 shared compounds between CCLE and GDSC in Fig. 3F, while the full list is provided in Supplementary Fig. 8. We took PLX4720 (a Raf kinase B inhibitor) as an example here. As shown in Fig. 3D (predicted ActArea of TCGA samples using the CCLE model) and 3E (predicted -LN(IC_50_) of TCGA samples using the GDSC model), there was a high proportion of samples with sensitivity to PLX4720 in SKCM (CCLE: 96.20%; GDSC: 97.01%) and UVM (CCLE: 98.75%; GDSC: 98.75%). All these enrichment patterns were replicated by both CCLE and GDSC models. Further investigation of cell lineage and cancer types versus drug response also revealed patterns that were consistent with previous reports^19^ (Supplementary Information and Supplementary Fig. 9).

### Replication of associations between compounds and their targets in TCGA

**Fig. 4:**
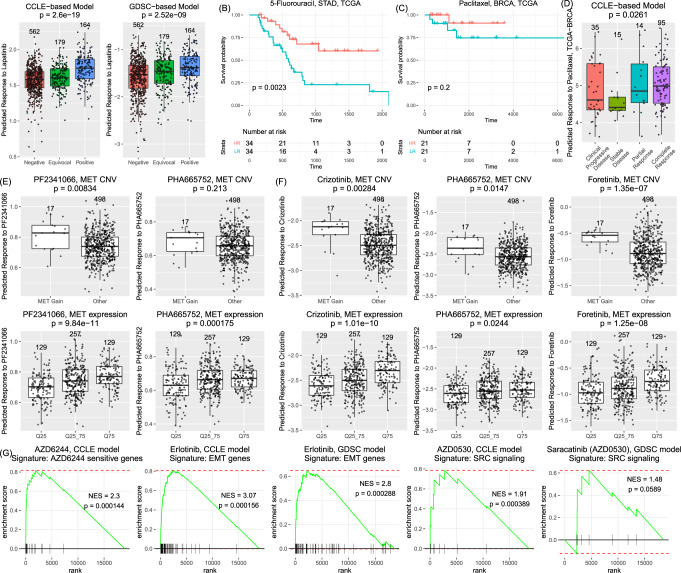
Validation using TCGA data. **A** Comparison of predicted response to lapatinib in different subtypes of TCGA-BRCA as defined using HER2 immunohistochemistry status (*n*_Negative_ = 562, *n*_Equivocal_ = 179, *n*_Positive_ = 164). The *P* value was obtained by fitting a linear regression model with the predicted response as the outcome variable and the sample status as the predictive variable (negative samples labeled as 0, equivocal samples labeled as 1, and positive samples labeled as 2). The predictive variable is considered as numerical to measure the increasing trend among the three groups. **B** Survival analysis of TCGA-STAD samples treated with 5-fluorouracil. **C** Survival analysis of TCGA-BRCA samples treated with paclitaxel. The *P* values in (**B**) and (**C**) were from a log-rank test comparing two groups of samples defined by the predicted response to the corresponding drug (HR: high response, greater than the median; LR: low response). **D** Comparison of predicted response to Paclitaxel in TCGA-BRCA samples stratified by response status. The *P* value was from a two-sided *t* test by comparing the disease group *(n* = 50, including samples annotated as clinical progressive disease (*n* = 35) and samples as stable disease (*n* = 15)) and the non-disease group *(n* = 109, including samples annotated as partial response (*n* = 14) and samples as complete response (*n* = 95)). **E** Association of predicted response to two MET inhibitors (PF2341066 and PHA665752) with MET amplification (top panel) or MET expression (bottom panel) using CCLE models. In the top panel, a two-sided *t* test was conducted to compare the predicted response in LUAD samples with MET gain (*n* = 17, for both the cases of PF2341066 and PHA665752) and other LUAD samples (*n* = 498, for both the cases of PF2341066 and PHA665752). In the bottom panel, samples were grouped into three groups based on *MET* expression: Q25 (*n* = 129, the lower quartile of all samples ordered by increasing MET expression), Q25_75 (*n* = 257, the middle half of samples), and Q75 (*n* = 129, the upper quartile). **F** Association of predicted response to three MET inhibitors (crizotinib (also known as PF2341066), PHA665752, and foretinib (only tested in GDSC)], with MET amplification (top panel) or MET expression (bottom panel) using GDSC models. In the top panel, a two-sided *t* test was conducted for LUAD samples with MET gain (*n* = 17) and other LUAD samples (*n* = 498) and in the bottom panel, a linear regression model was fit for three groups *n*_Q25_ = 129, *n*_Q25_75_ = 257, and *n*_Q75_ = 129, as in (**E**). **G** Validation using previously reported gene signatures to AZD6244, erlotinib, and AZD0530. The Normalized Enrichment Score (NES) and *P* values were obtained from Gene Set Enrichment Analysis (GSEA) implemented by the R package *fgsea* (1.16.0). In all boxplots (**A**, **D**, **E**), each box shows the interquartile range (IQR between Q1 and Q3) for the corresponding set. The central mark (horizontal line) shows the median and the whiskers show the rest of the distribution based on IQR (Q1−1.5 × IQR, Q3 + 1.5 × IQR).

Further validation using the drugs with well-annotated targets confirmed the high quality of the predicted drug response. These included lapatinib, MET inhibitors (crizotinib/PF2341066, foretinib, and PHA665752), BRAF inhibitors (PLX4720 and RAF265), and several MEK inhibitors. As shown in Fig. 4A, the predicted response to lapatinib (ERBB2 inhibitor) had a strongly significant association with HER2 immunohistochemistry status in TCGA-BRCA using the CCLE model (*P* = 2.60 × 10^−19^) as well as using the GDSC model (*P* = 2.52 × 10^−9^). In CCLE, two MET inhibitors were measured (PF2341066 and PHA665752), both of which showed significant association with increasing *MET* expression (*P* = 9.84 × 10^−11^ for PF2341066 and *P* = 1.75 × 10^−4^ for PHA665752, Fig. 4E) in TCGA-LUAD. The predicted response to these two compounds was also significantly higher in LUAD samples with *MET* amplification than other LUAD samples. In GDSC, three MET inhibitors were measured (PF2341066, known as crizotinib in GDSC, foretinib, and PHA665752). The predicted response to each of these compounds was also significantly associated with *MET* expression or *MET* amplification (Fig. 4F). For MEK and BRAF inhibitors, we examined the mutation status of *EGFR*, *KRAS*, *HRAS*, *NRAS*, and *BRAF* with these compounds (Supplementary Fig. 10). Overall, samples with mutant *EGFR/KRAS/HRAS/NRAS/BRAF* tended to have increased sensitivity to the corresponding kinase inhibitors in most cancer types, although some of the association did not reach significance (e.g., *EGFR* with erlotinib in LUAD, *P* = 0.16 by A-model and *P* = 0.046 by S-model). This is likely due to the complex tumor microenvironment and confounding factors such as co-occurrence of mutations, CNVs, and other omics-level complications.

In addition, some TCGA samples have recorded treatment history, such as paclitaxel in BRCA and 5-fluorouracil in STAD^20^. We conducted survival analyses of these samples by stratifying the samples using the predicted response. As shown in Fig. 4B, for STAD samples who were treated with 5-fluorouracil, the subgroup with a high predicted response to the drug had a significantly better survival outcome (*P* = 0.0023) than that with low predicted response. BRCA samples treated with paclitaxel showed marginal significance (*P* = 0.20, Fig. 4C), although the group annotated with complete or partial response had significantly higher response than the group with stable or clinical progressive disease (*P* = 0.03, Fig. 4D).

### Validation using gene expression signatures

To further validate the imputed drug response, we collected previously reported gene signatures for drugs. To examine the association between the gene expression of these signature genes and our imputed drug response, we fitted linear regression models. For each gene in each cancer type, we split samples into three groups according to the gene expression: samples with gene expression in the lower quarter of the expression range (Q25), samples in the middle half of the expression range (Q25–Q75), and samples in the higher quarter of the expression range (Q75). We fitted linear regression models by taking the group variable as a quantitative type such that the model evaluated the trend of the drug response changing across the three groups of samples (similar to a trend test instead of ANOVA). *t*-values were obtained from the model and were combined across all cancer types by $t = \mathop {\sum}\nolimits_{i = 0}^K {t_i/\sqrt K }$, following Stouffer’s Z-score method, where *K* is the total number of cancer types. With these *t*-values, we conducted GSEA to validate previously reported signatures. For the example of AZD6244, a previous study reported an 18-gene signature with sensitivity to AZD6244^12^. In our results, these genes were significantly associated with the response to AZD6244 (*P* = 1.44 × 10^−4^, Normalized Enrichment Score (NES) = 2.30, Fig. 4G, Gene Set Enrichment Analysis (GSEA)). Specifically, member genes such as *ETV4*, *ETV5*, *DUSP6*, and *SPRY2* were all among the 50 most positively associated genes with AZD6244 sensitivity. EMT genes were previously reported as predictors for erlotinib response^21^. In our results, we found a significant association between EMT genes with the predicted erlotinib response (*P* = 1.56 × 10^−4^ and NES = 3.07 for the CCLE model; *P* = 2.88 × 10^−4^ and NES = 2.80 for GDSC model). For AZD0530 (also known as saracatinib, a Src and Abl inhibitor), we found a previously reported gene set for SRC signaling^22^, which consisted of eight genes, was significantly associated with the drug in CCLE (*P* = 3.89 × 10^−4^, NES = 1.91) and marginally significant by the GDSC model (*P* = 0.059, NES = 1.48). It is worth noting that, although the drug response profiles were initially imputed using gene expression data at the low-dimensional space (i.e., latent vectors), there were several steps during the data encoding and decoding processes, including nonlinear transformations. Thus, there were no data circulation problems.

### Validation using independent data

**Fig. 5:**
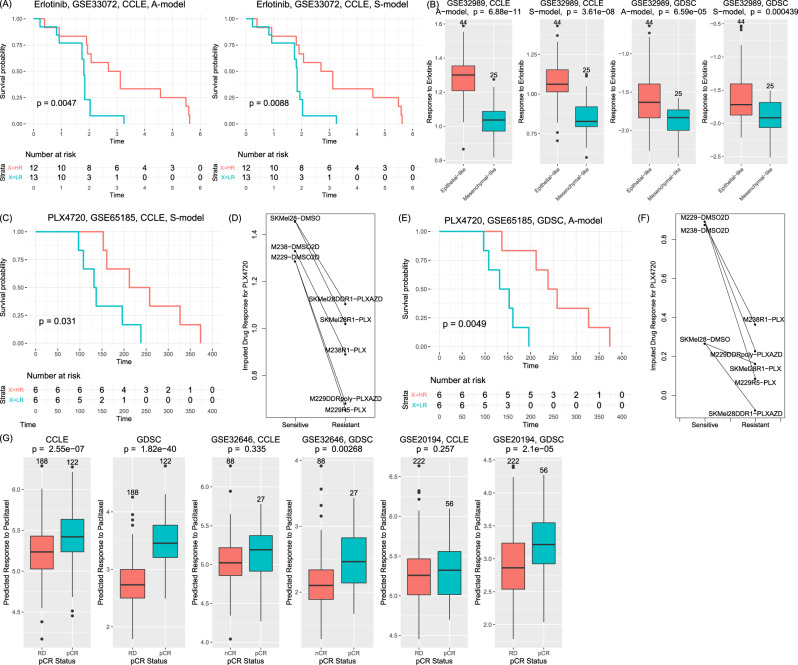
Validation using independent datasets. **A** Survival analysis of samples from GSE33072 who were treated with erlotinib. The *P* value was from a log-rank test comparing two groups of samples defined by the predicted response to erlotinib (HR: high response, greater than the median; LR: low response). **B** Comparison of predicted response to erlotinib in cell lines stratified into epithelial-like (*n* = 44) or mesenchymal-like (*n* = 25) group using the dataset GSE32989. The *P* value was obtained by using a two-sided *t* test. **C**, **E** Survival analysis of melanoma samples treated with vemurafenib (data from GSE65185). These samples were carriers with *BRAF* V600 mutations. The *P* value was from a log-rank test comparing two groups of samples defined by the predicted response to PLX4720 (HR: high response, greater than the median; LR: low response). **D**, **F** Comparison of predicted response to PLX4720 in parental cell lines and in derived resistant cell lines. **G** Comparison of predicted response to paclitaxel between BRCA subgroups with pCR (pathological complete response, *n*_pCR_ = 122 in GSE25055, *n*_pCR_ = 27 in GSE32646, and npCR = 56 in GSE20194) and RD (residual disease, also called nCR or non-pCR in GSE32646 (*n*_RD_ = 188 in GSE25055, *n*_nCR_ = 88 in GSE32646, *n*_RD_ = 222 in GSE20194). In all panels, the *P* value was obtained by using a two-sided *t* test. In the boxplots (**B**, **G**), each box shows the interquartile range (IQR between Q1 and Q3) for the corresponding set. The central mark (horizontal line) shows the median and the dots show the rest of the distribution based on IQR [Q1−1.5 × IQR, Q3 + 1.5 × IQR].

To validate the VAEN models, we collected six datasets with treatment and survival annotations. The first dataset (GSE33072) was part of the Biomarker-integrated Approaches of Targeted Therapy for Lung Cancer Elimination (BATTLE) trial^21^. We used the subset of samples treated with erlotinib and predicted their response to the drug using our VAEN models. As shown in Fig. 5A, by stratifying the samples using the median value of imputed drug response, we found a significant difference between the group with high response and the group with low response. Note that erlotinib was profiled by both the CCLE and GDSC models. This result was confirmed by using both the CCLE A-model (*P* = 0.0047) and the CCLE S-model (*P* = 0.0088). Using GDSC models, the high-response group showed marginally significant improvement in GDSC A-model (*P* = 0.058) and GDSC S-model (*P* = 0.056). As a comparison, the PCA + EN models failed to distinguish the samples. The gene + EN model trained using GDSC showed nominal significance but the model using CCLE failed to show the difference (Supplementary Fig. 11). The second dataset (GSE32989) included 68 non-small cell lung cancer (NSCLC) cell lines plus one normal lung cell line, all of which were profiled using the Illumina HumanWG-6 v2.0 expression beadchip^21^. Note that this dataset was generated by the microarray platform. However, because our model used rank-based normalization, we could conveniently transform the data and made predictions using the trained VAEN models. These cell lines have been studied to develop a 74-gene EMT signature. We repeated the original study and separated these cell lines into an epithelial-like subgroup (*n* = 44) and a mesenchymal-like subgroup (*n* = 25). As shown in Fig. 5B, the predicted response to erlotinib from all four VAEN models (CCLE A-model: *P* = 6.88 × 10^−11^, CCLE S-model: *P* = 3.61 × 10^−8^, GDSC A-model: *P* = 6.59 × 10^−5^, and GDSC S-model: *P* = 4.39 × 10^−4^) showed a significant difference between the two subgroups and the epithelial-like group had favored response, which was consistent with literature reports. The third dataset (GSE65185) included 24 melanoma patients carrying BRAF V600 who were treated with vemurafenib^23^. We used the predicted response to PLX4720 to distinguish the samples by the median value. As shown in Fig. 5, those samples with higher response to PLX4720 had better survival status (*P* = 0.031 using CCLE S-model and *P* = 0.0049 using GDSC A-model, Fig. 5C, E). Note that in CCLE, the S-model had both higher in-sample PCC and average holdout *R*^2^ and was selected for the prediction, whereas in GDSC, the A-model showed better performance and was selected. In contrast, both the PCA + EN model and the gene + EN model failed to distinguish samples with different survival statuses (Supplementary Fig. 12). In addition, this dataset included three parental cell lines (melanoma) and their derived resistant cell lines after treated with PLX or AZD compounds. Our results showed the parental cell lines had significantly higher response than the paired resistant lines (*P* = 8.032 × 10^−4^ by CCLE S-model, *P* = 0.017 by GDSC A-model, paired *t* test, Fig. 5D, F). We also collected three datasets for breast cancer patients with annotated pathological complete response (pCR) status^24–26^. pCR is defined as the status with the disappearance of all invasive cancer in breast cancer patients after completion of neoadjuvant chemotherapy. Achievement of pCR had been reported with favorable survival^27^. Our predicted response to paclitaxel was significantly higher in pCR group compared to the group with residual disease (RD) in all three datasets (Fig. 5G), indicating that the pCR group indeed had a better response to the chemotherapy compound. Collectively, these results proved that our predicted drug response was reliable in clinical data.

### Replication of drug similarity

**Fig. 6:**
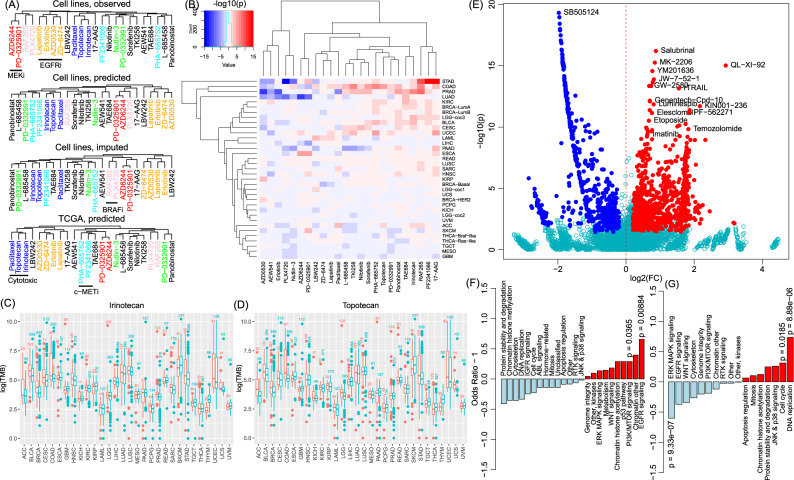
Drug similarity. **A** Hierarchy clustering results of the 24 CCLE drugs using four sets of samples: the observed drug response (set 1), the predicted drug response in cell lines with observed data (set 2), the predicted drug response in all 1100 cell lines (set 3), and the predicted drug response in all TCGA cancer samples. **B** Heatmap showing the association patterns between each drug and the tumor mutation burden (TMB) in CCLE. In each cell, red indicates a positive association (i.e., responders of the cancer type tended to have a high TMB), while blue indicates a negative association (i.e., responder tended to have a low TMB). For each cell, a two-sided *t* test was conducted by comparing the log10(TMB) of samples from the group with high sensitivity (i.e., “responders”, defined as the top 25% samples ordered by decreasing predicted response to the corresponding drug) and the log10(TMB) of the remaining 75% samples. The color was determined by whether the sensitive group had a higher average TMB than the other group. **C**, **D** Demonstration of TMB distribution in responders (the 25% samples with the highest response) and non-responders (the remaining 75% samples) using two cytotoxic drugs: irinotecan (**C**) and topotecan (**D**). Each box shows the interquartile range (IQR between Q1 and Q3) for the corresponding set. The central mark (horizontal line) shows the median and the dots show the rest of the distribution based on IQR (Q1−1.5 × IQR, Q3 + 1.5 × IQR). **E** Distribution of the association between GDSC drugs and TMB. Because TMB varied dramatically across cancer types, we conducted the test within each cancer type. Each dot represents a GDSC drug in a cancer type. The *P* value was similarly calculated as in (**B**). *x* axis is the log2 form of fold change (FC) defined as the average TMB of the responder group over the average TMB of the nonresponder group. Y axis indicates –log10 form of the unadjusted *P* value (for plotting only). Red and blue dots indicate significant associations (*P*_BH_ < 0.05 and log2FC > 0.2). **F**, **G** Enrichment of drug classes in the group of drug-cancer type associations with negative association patterns (blue dots in **E**) and with positive association patterns (red dots in **E**). The *P* values were obtained from a two-sided Fisher’s exact test to assess whether a drug class was overrepresented with the negative/positive associations.

For the 24 CCLE compounds, we did a hierarchical clustering using the three sets of drug response (ActArea) in CCLE and the predicted drug response in TCGA samples. Figure 6A shows consistent patterns of clusters for four EGFR inhibitors (AZD0530, erlotinib, lapatinib, and ZD-6474/vandetanib), two MEK inhibitors (AZD6244 and PD-0325901), two Raf kinase B inhibitors (PLX4720 and RAF265), and three cytotoxic compounds (paclitaxel, irinotecan, and topotecan) in four clustering analyses.

Drugs with cytotoxicity were found associated with a higher tumor mutational burden (TMB). In CCLE, the cytotoxic compounds (irinotecan and topotecan) had similar drug response profiles and were often clustered together. We examined the TMB, measured as a log-transformed number of mutations per sample, in TCGA samples. For each drug in each cancer type, we defined the top quantile (25%) most responsive samples as responders to the drug and the remaining samples as non-responders. By comparing TMB in responders with non-responders in each cancer type (Wilcoxon rank-sum test, two-sided), we found that in multiple cancer types, responders to cytotoxic compounds (irinotecan and topotecan) tended to have an increased TMB (Fig. 6B). This trend was observed in seven cancer types (BLCA, BRCA, COAD, ESCA, PAAD, and UCS) (Fig. 6C, D).

For GDSC compounds, there were 22 major groups plus unclassified ones. Compounds that were associated with abnormal TMB were found from different groups (Fig. 6E). The compounds associated with an increased TMB (nominal *P* < 0.01 and difference of TMB > 0.2) were statistically enriched with DNA replication (*P* = 8.88 × 10^−6^) and cell cycle (*P* = 0.019) (Fig. 6G). On the contrary, the compounds associated with a decreased TMB (nominal *P* < 0.01 and difference < −0.2) were statistically enriched with EGFR signaling (*P* = 8.84 × 10^−3^) and PI3K/mTOR signaling (*P* = 0.037) (Fig. 6F).

### Somatic mutations associated with imputed drug response

**Fig. 7:**
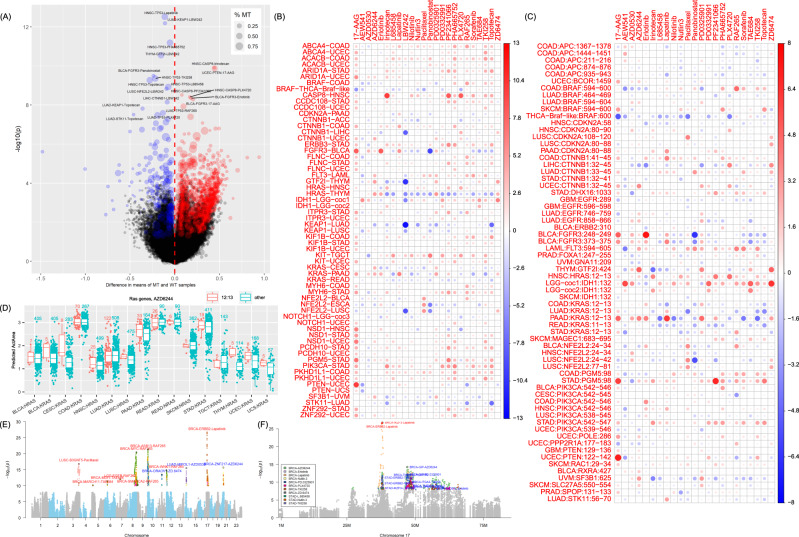
Association of somatic mutations with drug response. **A** Volcano plot of drug–gene association. The genomic signature was defined using the mutation status of each gene. Each dot represents the statistics of the association status of a gene in a cancer type with a drug. The *P* value was obtained from a two-sided Wilcoxon test comparing the predicted response in samples with the mutant gene (i.e., those with deleterious missense SNVs or nonsense SNVs) (MT) and samples with wild-type gene (WT). The former group was required to have at least ten samples. *x* axis: the difference of the average drug response in the samples harboring the mutated gene from that in the wild samples, *y* axis: −log10(*P*) where the unadjusted *p*-value was used for plotting. Red (difference >0) and blue (difference <0) dots indicate significant associations (BH-adjusted *P* < 0.05). **B** Heatmap showing the significant drug–gene associations in each cancer type. Due to space limitation, only associations with adjusted *P*_BH_ < 0.005 for positive association or *P*_BH_ < 0.01 for negative association were plotted. Associations involving the gene *TP53* were also excluded from plotting. The color and size of the circle in each cell is proportional to -log10(*p*) (note that we used the unadjusted *P* value for plotting). **C** Heatmap showing the significant drug–gene associations determined by mutation clusters. Each cell represents a mutation cluster of a specific gene in association with a specific drug. The *P* value was obtained from a two-sided Wilcoxon test comparing the predicted response in samples with mutations in a mutation cluster (i.e., only deleterious missense SNVs or nonsense SNVs considered) and samples with wild-type gene. Similarly, the former group was required to have at least ten samples to allow more tested clusters. **D** Distribution of the response to drug AZD6244 in the samples with two mutation clusters of Ras genes. Only the cases that had five or more mutated samples are shown. Each box shows the interquartile range (IQR between Q1 and Q3) for the corresponding set. The central mark (horizontal line) shows the median. **E** Association of drug response with copy number gain. **F** Association of drug response with copy number gain, focusing on chromosome 17 where *ERBB2* was located.

To identify genomic signatures that were associated with the imputed drug response, we conducted association analyses using multiple omics data in TCGA. Through this analysis, we aimed to validate the imputed drug response using the drugs with known targets (e.g., kinase inhibitors) and also to reveal novel drug–gene associations. For somatic mutations, we examined their potential association with each drug in each cancer type using deleterious single-nucleotide variants (SNVs). Moreover, considering that some targeted drugs are quite specific on particular mutations in kinase domains, we conducted the association test for mutation clusters according to their locations in the protein sequence. We split three cancer types into subtypes to avoid strong associations due to subtype stratification: BRCA to four subtypes (basal-like, Her2, luminal A, and luminal B)^28^, LGG to three subtypes (coc1, coc2, and coc3)^29^, and THCA to two subtypes (Braf-like and Ras-like)^30^ following the original marker publications of TCGA. A total of ~3000 candidate genes from 33 cancer types (39 if considering subtypes: 30 main cancer types plus 4 BRCA subtypes, 3 LGG subtypes, and 2 THCA subtypes) were investigated for the gene–drug association (required to have ≥10 mutations in at least one cancer type). Notably, this analysis was conducted using deleterious nonsynonymous SNVs. Benign nonsynonymous SNVs and small insertions and deletions (indels) were excluded. To control the false discovery rate, we conducted multiple test corrections for each drug in each cancer. As a result, we observed 2343 sensitive gene–drug associations and 288 insensitive associations for the 24 CCLE compounds (Benjamini–Hochberg (BH) adjusted *P*, or *P*_BH_, <0.05) (Fig. 7A). Among the genes whose somatic mutations showed significant association with increased drug response, we found several genes from the phosphoinositide 3-kinase (PI3K)/protein kinase B (AKT)/mammalian target of rapamycin (mTOR), i.e., the PAM pathway. They were *FGFR3*, *HRAS*, *KRAS*, *BRAF*, *NRAS*, *TP53*, *KIT*, and *NFE2L2* (Fig. 7B). We also observed drug–gene associations that were consistent with their known targets or pathways. For example, *ARID1A*, which is part of the ATP-dependent chromatin remodeling complex SNF/SWI, was associated with sensitivity to topotecan, a DNA topoisomerase I inhibitor, in STAD (*P*_BH_ = 1.68 × 10^−3^) and nominally significant in UCEC (raw *P* = 0.016). *FLT3* was significantly associated with sorafenib in LAML (*P*_BH_ = 2.03 × 10^−5^), a multikinase inhibitor, including *FLT3*. Association test of somatic mutations with GDSC compounds was also conducted (Supplementary Fig. 13).

We examined the associations between mutation clusters and drug response. We clustered mutations that were located within five amino acids in the protein sequence of a transcript and applied the Wilcoxon test on the predicted drug response in samples with the mutations in a cluster versus the samples without any mutations (wild type). The samples with mutations outside of the investigated cluster were not included in this test, so the wild-type samples were free of mutations in the same gene. In total, we identified 723 cluster–cancer–drug associations (*P*_BH_ < 0.2, after excluding associations involved in long genes, defined as those with >200 k bases), involving 116 clusters from 32 unique genes in 24 cancer types (Fig. 7C). For example, the aforementioned gene *FLT3* had a mutation cluster at 594–605 that was associated with Sorafenib (*P*_BH_ = 5.18 × 10^−3^). Other clusters were also observed in *EGFR*, *FGFR3*, *IDH1* (R132), *PIK3CA*, *SF3B1*, *SPOP*, and *VHL* (Fig. 7C).

Mutations on the G12/G13 positions in Ras proteins, including *HRAS*, *KRAS*, and *NRAS*, showed increased sensitivity to MEK inhibitors AZD6244 and PD-0325901 in multiple cancer types (BLCA, CESC, HNSC, PAAD, THYM, UCEC, and UCS) (Fig. 7D). However, we also observed some indifferent distributions, such as KRAS in COAD, LUAD, and READ. This might be due to cancer sample heterogeneity such as subtypes, the co-occurrence of mutations, and confounders such as CNVs.

The gene–drug association due to copy number changes occurred in several chromosomes, especially chromosomes 3, 7, 8, 10, 12, and 17 (Fig. 7E). Amplification of a region on chromosome 7, where *EGFR* was located, was mainly associated with kinase inhibitors in LGG, such as 17-AAG (*P* = 1.10 × 10^−8^) and RAF265 (*P* = 9.76 × 10^−13^). Chromosome 8, where the proto-oncogene *MYC* was located, was mainly amplified in BRCA and was associated with multiple drugs (*P*(17-AAG) = 1.76 ×10^−9^, *P*(AZD0530) = 4.62 × 10^−11^, *P*(irinotecan) = 3.26 × 10^−13^, *P*(TAE684) = 1.80 × 10^−16^, *P*(RAF265) = 3.67 × 10^−21^). Notably, the EGFR inhibitor, lapatinib, was significantly associated with *ERBB2* amplification (chr17, *P* = 2.30 × 10^−27^), which further confirmed the accuracy of our VAEN models. As for CNV loss, deletion of *CDKN2A/CDKN2B* on chromosome 9 was associated with multiple drugs in multiple cancer types. On chromosome 16, a region with the gene *WWOX* deletion in ESCA was significantly associated with multiple drugs (*P*(AZD6244) = 5.89 × 10^−18^, *P*(paclitaxel) = 3.31 × 10^−17^, *P*(PD-0325901) = 4.85 × 10^−18^, and *P*(TAE684) = 1.43 × 10^−15^). Notably, this region represents one of the most common chromosomal fragile sites and deletion of *WWOX* frequently occurs in multiple cancer types^31^. Hence, the driver event that leads to the strong association with drug response remains unclear.

### Drug-response profile associated with tumor microenvironment

Drug response in cancer patients is much more complicated than in cell lines due to the complex tumor microenvironment, tissue composition (e.g., purity and ploidy), and co-occurrence and interaction among different levels of alterations. Tumor tissues are surrounded and infiltrated with cells that are collectively known as tumor microenvironment (TME). TME consists of extracellular matrix (ECM), fibroblast, neuroendocrine cells, adipose cells, and immune and inflammatory cells and is involved in nearly every step of tumorigenesis^32^. Interestingly, we found that several of these TME cells were associated with drug response. First, cancer-associated fibroblast (CAF) genes and extracellular matrix (ECM) genes were negatively associated with lapatinib sensitivity, i.e., increased CAF/ECM gene expression was associated with insensitivity to lapatinib. Although lapatinib is a HER2 inhibitor and mainly used in the BRCA HER2 + subtype, this negative correlation trend was observed in many cancer types (23 out of the 39 cancer types (30 main cancer types plus 4 BRCA subtypes, 3 LGG subtypes, and 2 THCA subtypes), *P* < 0.05 and NES < 0, GSEA). It stays in line with previous observations that response to the HER2 inhibitor is dependent on the microenvironment of tumor samples^33^, where stroma genes^34^ and CAF genes played roles in resistance to both chemo- and targeted therapies^35^. In our work, we found that many stroma genes were among the most negatively associated genes with lapatinib sensitivity. The two EMT-related genes, *ZEB1* (*t* = −16.71) and *SNAI2* (*t* = −13.94), were both negatively associated with the response to lapatinib.

**Fig. 8:**
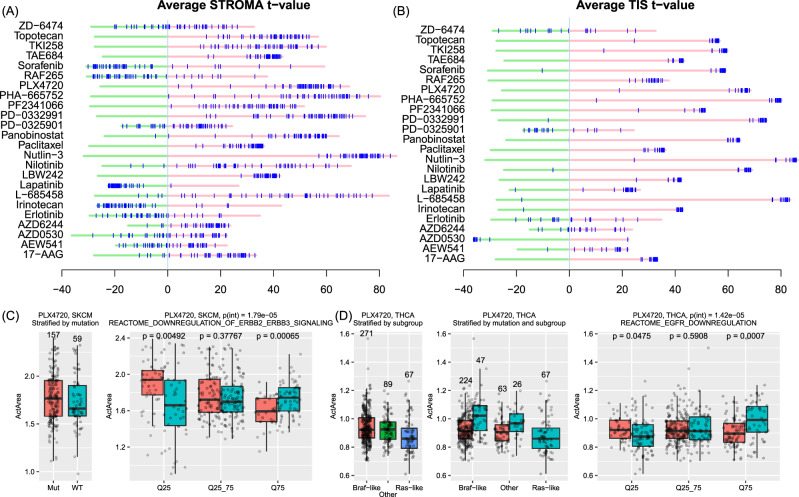
Drug response with tumor heterogeneity. **A** Distribution of gene t-scores for all drugs, with stroma genes, highlighted. Stroma genes were found to be negatively associated with several EGFR inhibitors, such as AZD6244, lapatinib, and PD-0325901. **B** Distribution of gene t-scores with Tumor Inflammation Score (TIS) signatures. **C** Investigation of the association between *BRAF* mutations and PLX4720 response in SKCM. SKCM samples with BRAF V600 mutations (labeled as “Mut” on the left panel, *n*_Mut_ = 157) did not show a significantly high response to PLX4720 compared to other SKCM samples that had no mutations in *BRAF*, *HRAS*, *KRAS*, *NRAS*, or *EGFR* (labeled as “WT” on the left panel, *n*_WT_ = 59). However, this was explained using the “downregulation of ERBB2 ERBB3 signaling” pathway activity. In the subgroup where this pathway showed low activity (the lower quartile of all samples ordered by increasing pathway activity, labeled Q25, *n*_Q25_ = 91, including *n*_Mut_ = 48 for samples with BRAF V600 and *n*_WT_ = 43 for samples without BRAF V600), samples with BRAF mutations had a higher response, implying that the pathway had a confounding effect on the response. In the middle subgroup (Q25_75), there are *n*_Q25_75_ = 180 samples with *n*_Mut_ = 80 samples harboring BRAF V600 mutations and *n*_WT_ = 100 samples harboring not. In the higher quantile subgroup (Q75), there are *n*_Q75_ = 91, including *n*_Mut_ = 29 for samples with BRAF V600 and *n*_WT_ = 62. A two-sided *t* test was conducted in each subgroup to compare the predicted response between the samples with the BRAF V600 mutation (*n*_Mut_) and samples without BRAF V600 mutations (*n*_WT_). **D** Investigation of the association between *BRAF* mutations and PLX4720 response in THCA. THCA samples were grouped into three subtypes according to the original publication: Braf-like (*n* = 271, where *n* = 224 harboring BRAF V600 mutations and *n* = 47 not), Ras-like (*n* = 67, where all having not the BRAF V600 mutation), and others (*n* = 89, where 63 harboring BRAF V600 mutations and *n* = 26 not). When stratifying the samples by the EGFR downregulation pathway activity, the samples in each subgroup were as below: *n*_Mut_ = 42 and *n*_WT_ = 81 in the Q25 subgroup, *n*_Mut_ = 170 and *n*_WT_ = 74 in the Q25_75 subgroup, and *n*_Mut_ = 75 and *n*_WT_ = 48 in the Q75 subgroup. In each subgroup, a two-sided *t* test was conducted to compare the response between the samples with BRAF V600 mutations (*n*_Mut_) and samples without (*n*_WT_). In the boxplots (**C**, **D**), each box shows the interquartile range (IQR between Q1 and Q3) for the corresponding set. The central mark (horizontal line) shows the median.

Second, we explored 18 genes that were previously reported with the predictive power of T-cell inflammation^36^ (referred as TIS genes). As shown in Fig. 8, several drugs showed strong positive associations with TIS genes (irinotecan, nilotinib, PHA665752, PLX4720, and RAF265), while other drugs (e.g., AZD6244 and PD-0325901) showed no association with TIS genes. Because the expression of TIS genes is highly indicative of immune-hot or immune-cold status, these results suggested that the samples with a high level of T-cell inflammation (e.g., immune-hot) might be more sensitive to PLX4720 and RAF265. This is also in line with the previous reports^17^. Notably, these results for studying tumor microenvironment are hypothetical without validation and future work is needed to warrant the validity of the results.

In addition to the tumor microenvironment, tumor heterogeneity is also a factor to influences drug response. In examining the mutation–drug association, we noticed in some cases, the results were inconsistent with a priori knowledge of drug targets. For example, *BRAF* mutations showed no association with BRAF inhibitor PLX4720 in SKCM (*P* = 0.11, two-sided *t* test between the BRAF V600E carriers and the samples without mutations in *BRAF/HRAS/KRAS/NRAS*) or THCA (*P* = 0.94, same as above). As reported previously^17^, in cell lines, response to BRAF inhibitors was found influenced by *EGFR* expression (Supplementary Fig. 5). In cancer samples, we found the confounding effect of *EGFR* itself was not significant. However, when we repeated the analysis using pathway activities, we found that the activities of the related EGFR signaling pathways, instead of *EGFR* expression, were better predictors for explaining the association between *BRAF* mutations and response to BRAF inhibitors. To this end, we calculated the activity of 1054 pathways from MSigDB^37^ using ssGSEA^38^ and fit regression models with an interaction factor between *BRAF* mutation status and categorized pathway activities: **Y**_response_ ~ **X**_mut_ + **X**_pathway_ + **X**_mut_ × **X**_pathway_, where **X**_mut_ is a categorical variable indicating whether a sample harboring the *BRAF* V600 mutations and **X**_pathway_ is a categorical variable with three levels of pathway activity (Q25, Q25-75, and Q75, the same definition as for *EGFR* expression). For example, in SKCM, when the activity of “downregulation of ERBB2 ERBB3 signaling pathway” was included in the model, the interaction item was statistically significant (*P* = 1.79 × 10^−5^) and only in the Q25 subgroup, where the pathway showed low activity, that group with mutant *BRAF* was sensitive to BRAF inhibitors (*P* = 4.92 × 10^−3^) (Fig. 8C). In THCA, response to PLX4720 was driven by the cancer subtypes—the Braf-like subtype had a significantly higher response than the Ras-like subtype (A-model: *p* = 2.72 × 10^−16^; S-model: *P* = 8.31 × 10^−4^; two-sided *t* test, Fig. 8D). The Braf-like subtype included THCA samples that harbored BRAF V600 mutations, other *BRAF* mutations, and RET fusion, while the Ras-like subtype included the samples with mutations in H/K/NRAS genes. Thus, the predicted response is consistent with the *BRAF* mutation status. Nonetheless, EGFR related pathways still played roles in the response, such as EGFR downregulation (*P* = 1.42 × 10^−5^). In the Q25 subgroup where the pathway EGFR downregulation had low activity, the samples with mutant *BRAF* were significantly sensitive to BRAF inhibitors (*P* = 0.048). These results revealed that drug response in cancer samples is much more complex than in cell line models and many confounding factors could impact the response.

## Discussion

In this study, we have built a variational autoencoder model to regenerate gene expression profiles in > 1000 cell lines. The latent vectors, the internal principal variables, were used to build prediction models for a total of 261 compounds from two pharmacogenomics projects (24 CCLE and 251 GDSC compounds). The imputed drug response profiles in 33 cancer types, augmented with comprehensive multi-omics data from TCGA, provided a useful resource to further explore genomic signatures that are associated with drug response.

Our methods have many advantages and the results have made it possible to explore the drug response of cancer samples on a much fine scale. First, although the previous research has made great efforts to reveal genomic signatures associated with drug sensitivity and resistance, these results were mainly based on cell line models. An accurate prediction of drug response profile in cancer samples would enable the recapitalization of known signatures and novel signatures, whereas the latter ones have been often missed due to cell line models or sample size limitation. Second, our analysis of the classification of compounds based on their response profiles revealed unique groups and signatures. Although it has been previously noticed that targeting compounds designed for particular genes or mutations and compounds with general cytotoxicity belong to two generally different groups of drugs, our examination of TCGA data identified the association between drugs and TMB, which was infeasible from cell line models. Third, we explored many aspects to identify genomic signatures in a pan-cancer fashion, including somatic mutations, somatic CNVs, and gene expression. The positive associations between AZD6244 and the previously reported 18-gene signature proved the robustness and accuracy of our results. The negative associations between CAF genes were insightful for breast cancer therapeutic selection. In addition, we found several drugs were positively associated with increased tumor inflammation scores. This is promising, indicating that cancer samples with higher TIS (i.e., tend to be immune-hot) are more sensitive to those drugs, especially those BRAF inhibitors.

Our method also has several technical advantages. First, we trained 100 autoencoders to represent the original cell lines. This process provided a pool of latent vectors (i.e., predictors) for drug response. Notably, overfitting has been a widely recognized challenge in many machine learning and deep-learning applications. To overcome the potential overfitting problem, we used the out-sample PCC to select prediction models. We actually attempted to use the canonical measurements such as accuracy and F1 score. We found that using the out-sample *R*^2^ had the least overfitting, as evaluated by using the 14 shared drugs in TCGA samples that were predicted by CCLE models and by GDSC models (data now shown). Notably, we trained the model using the data within one panel only (e.g., CCLE), and then evaluated the performance (e.g., tenfold cross-validation) using the data in the same panel. Thus, a model trained on one panel is independent of that trained on the other panel, even for the 14 drugs that were profiled by both the CCLE and GDSC panels. Second, we explored different normalization methods and activation functions to identify the model with the best performance yet with high generalizability. With rank-based normalization of gene expression data, our method can be applied to a wide variety of datasets, including microarray-based data, as shown in Fig. 5. Third, we have applied several approaches to identify genomic signatures that are associated with each drug.

There were several limitations of our work. For some drugs, we were unable to improve the prediction accuracy no matter how the models were fit, such as LBW242 (Fig. 2). For some other drugs, VAE based models could not compete with PCA-based models regarding the model-fitting parameters (in-sample PCC and holdout *R*^2^). Two example drugs are 17-AAG and Paclitaxel. However, with limited data for validation, the PCA-based model for paclitaxel did not perform well to separate the pCR group from the non-pCR group. Therefore, future validation would be required to assess these prediction models. Furthermore, for some drugs, although we observed high prediction accuracy in the cell line models, their response in cancer samples was heterogeneous. We could explain the association between BRAF mutations and BRAF inhibitor PLX4720. However, for other drugs, such as erlotinib, we only observed a marginally significant association between *EGFR* and erlotinib, and we were not able to figure out the potential confounding factors. This could be due to mutations in other genes or other forms of mutations (e.g., CNV, methylation, or post-transcriptional regulation), which could complicate the response to erlotinib. Therefore, investigation of drug response in cancer samples is much more complicated and requires consideration of many contexture factors and covariates.

## Methods

### Data collection and preprocessing

#### Baseline data

The Cancer Cell Line Encyclopedia (CCLE)^1^ project has assessed gene expression in 1156 cell lines (version July 18, 2018). We downloaded RPKM values from RNA-sequencing (RNA-seq) data. Each cell line has its original cell lineage. The pool of tested cell lines could be matched to a wide range of cancer types, including both solid cancers and hematopoietic and lymphoid tissues. We excluded the lineages that had less than 20 samples. Our working dataset included 1100 cell lines from 19 cell lineages, which were used to build the VAE models. We selected the most variably expressed genes to construct the VAE models. Note that the same gene expression data in those cell lines were used for drug response prediction for CCLE and GDSC drugs.

#### Drug-response data

The CCLE project assessed the pharmacological profiles of 24 anticancer compounds in 504 cell lines^1^. We downloaded data from the CCLE (https://portals.broadinstitute.org/ccle) data portal, including mutations and drug response measured by the activity area (named “ActArea”). The GDSC project assessed drug response for 251 compounds using the same pool of cell lines. We downloaded the fitted dose-response file from the GDSC website (https://www.cancerrxgene.org/, Release 7.0, version 17.3, access date: August 29, 2018). In this study, we used the log-transformed IC50 (LN_IC50) in the Elastic Net models, although AUC can also be used for the same purpose.

#### TCGA multi-omics data

All data were downloaded from the UCSC Cancer Genome Browser Xena^39^. We retrieved the TCGA samples that had somatic mutations, CNVs, and mRNA expression data. The cancer type abbreviations are listed in Fig. 1. For SNVs and indels, we downloaded the Unified Ensemble “MC3” gene-level mutation calls from Xena. We defined deleterious missense SNVs as those annotated as deleterious by SIFT and damaging by PolyPhen, which was provided by the MC3 annotations. CNV data were obtained from the Affymetrix 6.0 platform and had 5 levels to represent different CNV status: deep deletion (CN = −2), copy loss (CN = −1), neutral (CN = 0), copy gain (CN = 1), and amplification (CN = 2). mRNA expression data from RNA-seq were downloaded in the form of log2(RPKM + 1) value.

#### Preprocess of gene expression data

All RNA-seq data were transformed using the log2(RPKM + 1) format. Transformation based on rank-reversed percentile was used to preprocess the RNA-seq and microarray data.

### VAEN model training

We implemented a three-layer VAE model, with the input layer, encoder, and latent layer, decoder, and output layer (Fig. 1A). The python library for deep learning, Keras (version 2.1.6)^40^ with a TensorFlow backend (version 1.0.1)^41^, was used to implement the VAE. The encoder is a process to encode the input vector with a mean vector and a standard deviation vector, respectively, followed by a nonlinear transformation, e.g., the rectified linear units (ReLU) or the Sigmoid activation (see main text). We defined the loss function as the mean squared error plus the KL loss. As for VAEN, a regression model was trained for each drug following a Elastic Net^42^ strategy with 5-fold cross-validation to select lambda. We used the average *R*^2^ in the holdout samples to select the model. CCLE models were trained using CCLE drug response and CCLE genomics data. GDSC models were trained using GDSC drug response and CCLE genomics data.

### Association test

Drug–gene associations based on somatic mutations were assessed using Wilcoxon rank-sum test by comparing the predicted drug response in the mutant samples versus the wild-type samples for each gene. Drug–gene associations based on gene expression were assessed using the Pearson correlation coefficient.

### Reporting summary

Further information on research design is available in the Nature Research Reporting Summary linked to this article.

## Supplementary information

Supplementary Information

Reporting Summary

## Data Availability

The original CCLE, GDSC, and TCGA data are publicly available datasets. CCLE data were downloaded from https://portals.broadinstitute.org/ccle. GDSC data were downloaded from the GDSC Website https://www.cancerrxgene.org/. TCGA data were downloaded from UCSC Cancer Genome Browser Xena^39^. The intermediate files as well as the result files are available at https://github.com/bsml320/VAEN/ and https://bioinfo.uth.edu/VAEN/. All relevant data are available from the authors. Source data are provided with this paper.

## Source data

Source Data

## Code availability

All source codes are available at https://github.com/bsml320/VAEN/.
