# Supplementary material for: Deep generative neural network for accurate drug response imputation
Source: Nat Commun. 2021 Mar 19;12:1740. doi: 10.1038/s41467-021-21997-5 (PMC7979803; doi:10.1038/s41467-021-21997-5)
Supplement: Supplementary file 1 — Supplementary Information [file 41467_2021_21997_MOESM1_ESM.pdf]

Supplementary Information for “Deep generative neural network for accurate drug response imputation”

Impact of cell lineage and cancer types on drug response

Some drugs were previously shown to have tissue-specific effect. We tested for the potential association between drug response and cell line lineage or cancer type. Using Fisher’s Exact Test, we found that the haematopoietic and lymphoid cell lines were particularly sensitive to many drugs using both the observed and predicted ActArea in CCLE (Supplementary Figure 9A**,** the red pies). Such a trend was similarly observed in GDSC data (Supplementary Figure 9B). Several other pairs of tissue/cell lines and MEK inhibitors were observed for sensitivity, including large intestine cell lines with AZD6244 and PD-0325901 and pancreas to PD-0325901. In the cancer data (Supplementary Figure 9C and 9D), HNSC, LGG, and LUSC showed strong sensitivity to many compounds (red boxes), while BRCA, KIRC, KIRP, and LIHC were insensitive to many compounds (blue boxes). From both the cell line data and cancer data, cell lines of skin origin (CCLE) and skin tumors (TCGA) were found sensitive to MEK and BRAF inhibitors, such as AZD6244, PD−0325901, PLX4720, and RAF265.

Supplementary Table 1. Model stability

	Rank Sigmoid	Rank ReLU	ZS Sigmoid	ZS ReLU	Z01 Sigmoid	Z01 ReLU
**17-AAG**	0.756	0.662	0.701	0.670	0.378	0.324
**AEW541**	0.658	0.623	0.710	0.461	0.166	0.156
**AZD0530**	0.754	0.587	0.788	0.643	0.589	0.292
**AZD6244**	0.838	0.674	0.837	0.648	0.419	0.259
**Erlotinib**	0.881	0.839	0.850	0.756	0.618	0.506
**Irinotecan**	0.776	0.752	0.708	0.592	0.628	0.364
**L-685458**	0.907	0.767	0.872	0.722	0.846	0.284
**Lapatinib**	0.947	0.823	0.950	0.764	0.601	0.593
**LBW242**	0.368	0.491	0.402	0.351	0.937	0.235
**Nilotinib**	0.858	0.637	0.782	0.551	0.378	0.152
**Nutlin-3**	0.829	0.763	0.812	0.784	0.884	0.376
**Paclitaxel**	0.791	0.773	0.730	0.692	0.659	0.492
**Panobinostat**	0.792	0.750	0.826	0.661	0.611	0.516
**PD-0325901**	0.837	0.619	0.854	0.653	0.366	0.223
**PD-0332991**	0.850	0.779	0.825	0.694	0.784	0.424
**PF2341066**	0.771	0.816	0.748	0.761	0.671	0.487
**PHA-665752**	0.833	0.644	0.767	0.529	0.929	0.502
**PLX4720**	0.921	0.866	0.881	0.826	0.656	0.252
**RAF265**	0.838	0.742	0.806	0.645	0.472	0.520
**Sorafenib**	0.904	0.779	0.871	0.786	0.702	0.542
**TAE684**	0.722	0.570	0.678	0.520	0.293	0.413
**TKI258**	0.786	0.717	0.807	0.752	0.431	0.402
**Topotecan**	0.804	0.766	0.811	0.677	0.462	0.532
**ZD-6474**	0.874	0.603	0.844	0.604	0.564	0.249

*The values were the average Pearson correlation coefficient of the predicted response in all TCGA samples by the top 10 best models.

Supplementary Table 2. Model evaluation using TCGA data

	Target	Compound	Rank Sigmoid		ZS Sigmoid		PCA+EN		Gene+EN	
			t	***p*-value**	***t***	***p*-value**	***t***	***p*-value**	***t***	***p*-value**
**CCLE**	ERBB2	Lapatinib	9.19	2.60E-19	4.93	9.71E-07	9.41	4.11E-20	17.48	3.54E-59
**GDSC**	ERBB2	Lapatinib	6.02	2.52E-09	2.77	5.71E-03	5.14	3.42E-07	5.36	1.05E-07
**CCLE**	MET	PF2341066	6.61	9.84E-11	4.02	6.84E-05	6.22	1.02E-09	4.46	9.89E-06
**CCLE**	ALK[#]	PF2341066	2.68	7.68E-03	4.62	4.87E-06	2.60	0.01	3.13	1.88E-03
**CCLE**	MET	PHA.665752	3.78	1.75E-04	2.67	7.89E-03	8.16	2.53E-15	2.13	0.03
**CCLE**	ALK[#]	PHA.665752	2.69	7.38E-03	5.46	7.52E-08	3.26	1.17E-03	1.00	0.32
**GDSC**	MET	Crizotinib	6.60	1.01E-10	-2.81	5.14E-03	1.62	0.106	-0.81	0.42
**GDSC**	ALK[#]	Crizotinib	0.88	0.38	-5.17	3.39E-07	-1.76	0.078	-2.11	0.04
**GDSC**	MET	Foretinib	5.79	1.25E-08	4.95	1.01E-06	4.73	2.86E-06	2.47	0.01
**GDSC**	ALK	Foretinib	2.54	1.14E-02	1.73	8.49E-02	-1.37	0.17	-1.72	0.09
**GDSC**	MET	PHA.665752	2.26	0.024	1.51	0.131	-2.32	0.021	-0.02	0.99
**GDSC**	ALK	PHA.665752	3.85	1.32E-04	-2.21	0.0276	-4.18	3.50E-05	1.27	0.21
**CCLE**	pCR[$]	Paclitaxel	2.26	0.026	0.08	0.469	0.18	0.86	-1.19	0.24
**GDSC**	pCR	Paclitaxel	-0.02	0.99	-0.49	0.687	0.30	0.382	-0.02	0.49

*Grey cells: the direction of the association is opposite, or the association is insignificant.
[#]ALK is not the primary target but could also be targeted by the MET inhibitors. Thus, the association is expected to be moderate.
[$]pCR: pathological complete response.

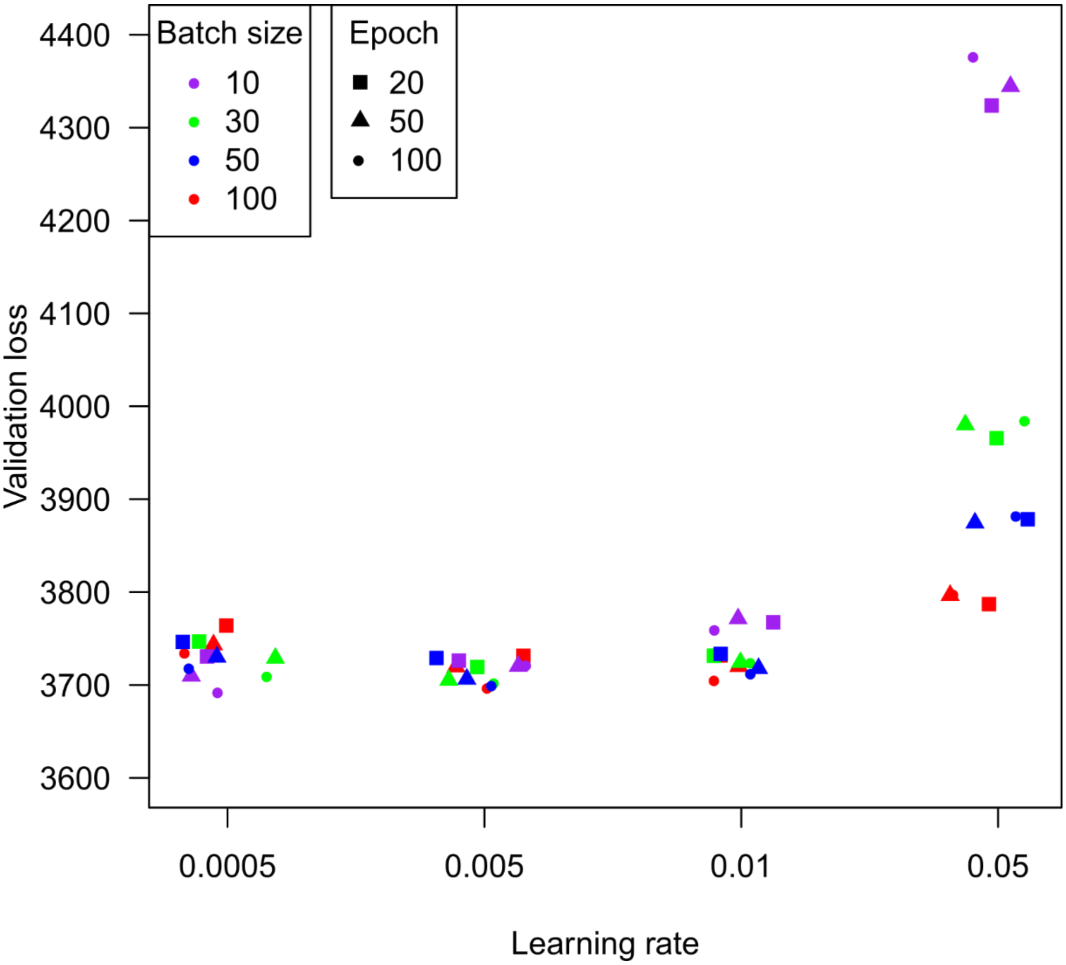

Supplementary Figure 1. The hyperparameter sweep result for different VAE models. The final model was selected with batch size being 100, 100 epochs, and learning rate being 0.0005. See more details in main text.

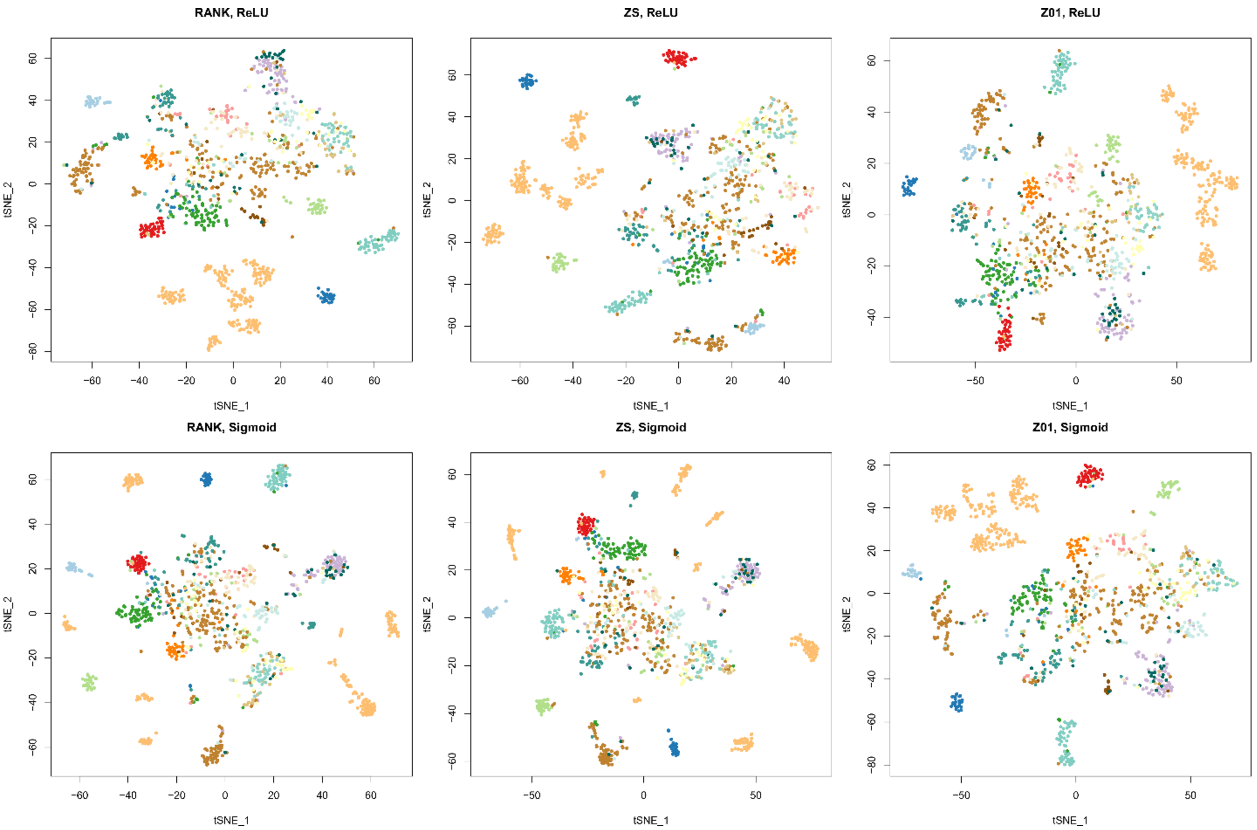

Supplementary Figure 2. tSNE plots of different VAE compression models. tSNE: t-distributed stochastic neighbor embedding. In each panel, a dot represents a cell line. The legend of node color is the same as in Figure 1.

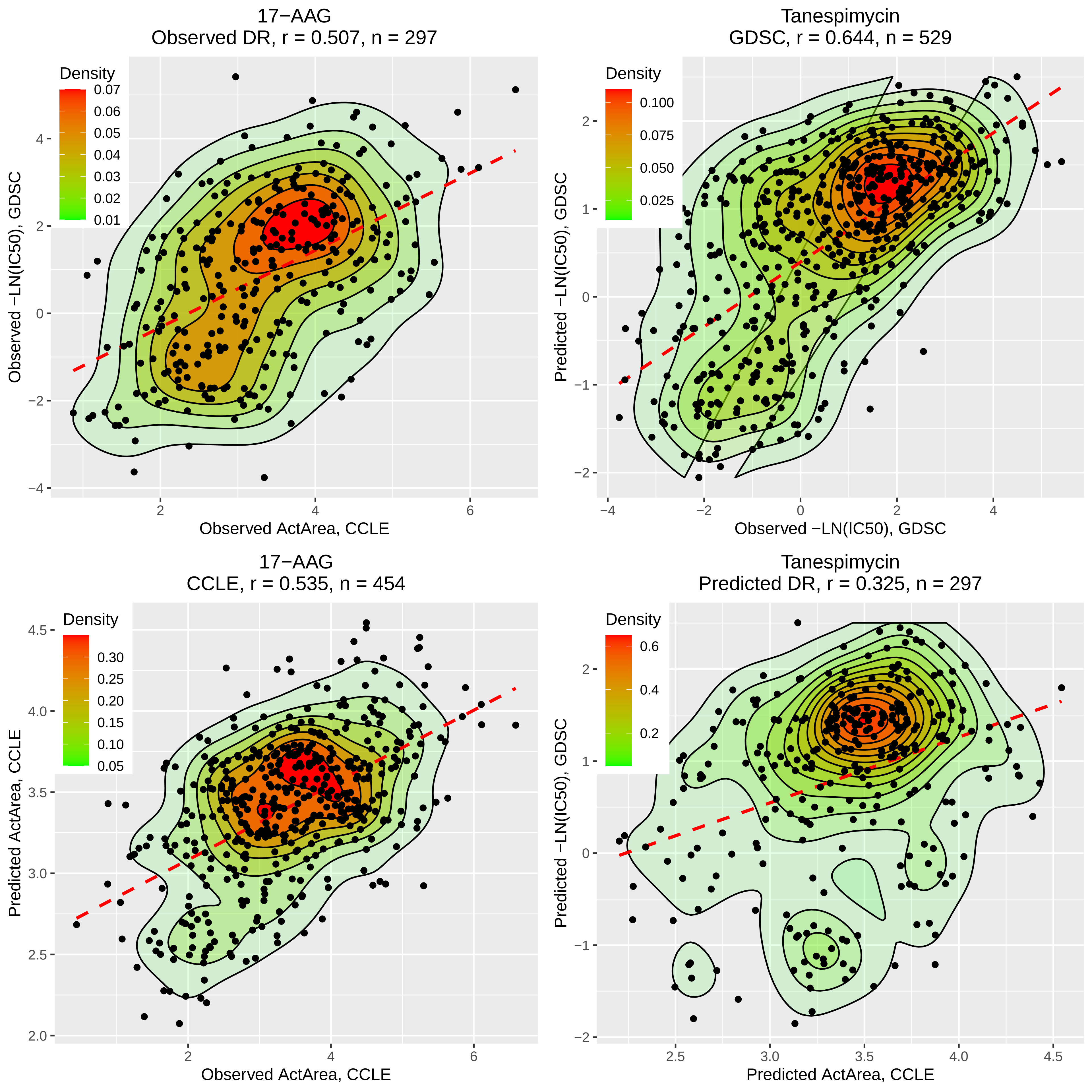

17−AAG
Observed DR, r = 0.507, n = 297
Density
Observed −LN(IC50), GDSC
Observed ActArea, CCLE
Tanespimycin
GDSC, r = 0.644, n = 529
Predicted −LN(IC50), GDSC
Observed −LN(IC50), GDSC
17−AAG
CCLE, r = 0.535, n = 454
Predicted ActArea, CCLE
Observed ActArea, CCLE
Tanespimycin
Predicted DR, r = 0.325, n = 297
Predicted −LN(IC50), GDSC
Predicted ActArea, CCLE

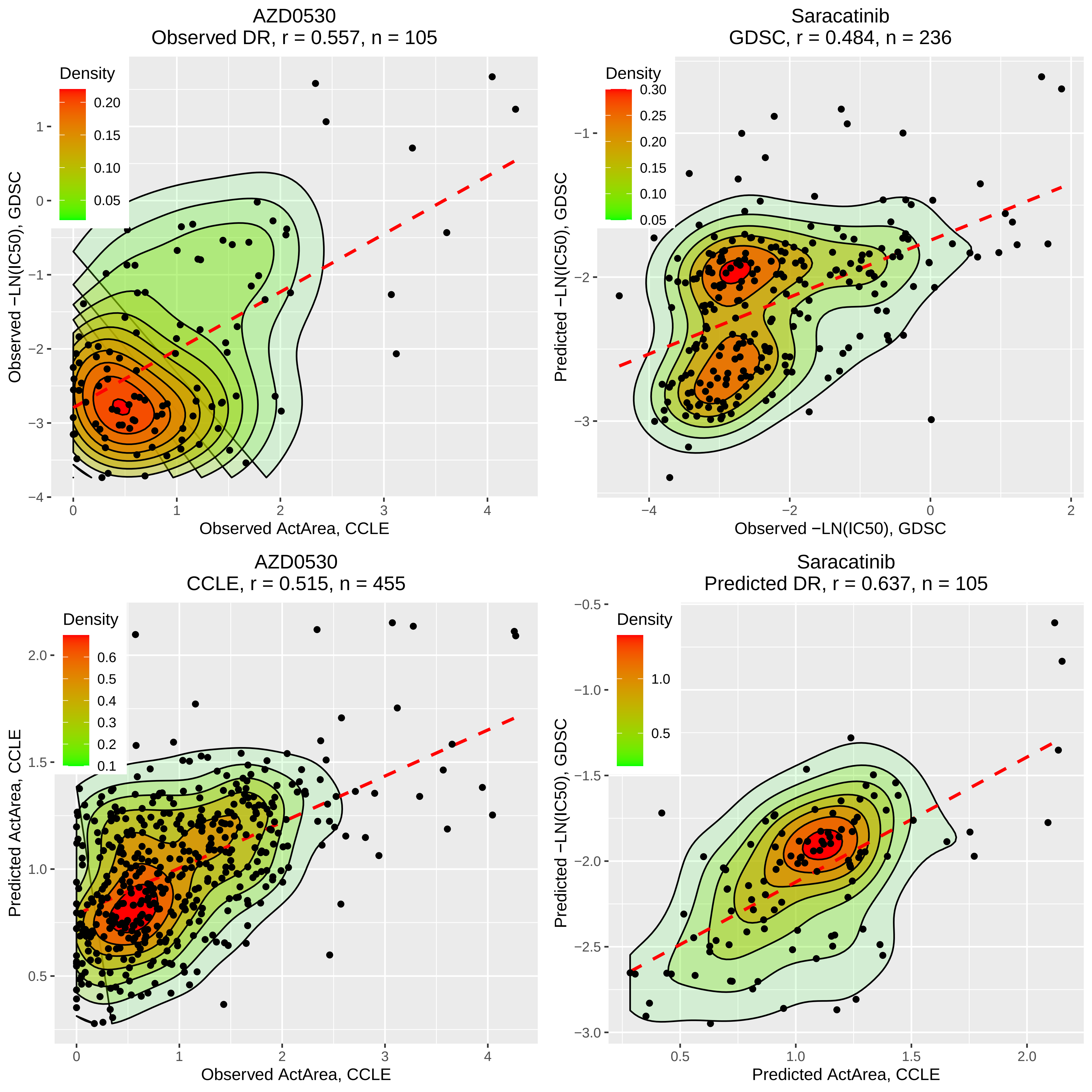

AZD0530
Observed DR, r = 0.557, n = 105
Density
0.20
0.15
0.10
0.05
Observed −LN(IC50), GDSC
Observed ActArea, CCLE
Saracatinib
GDSC, r = 0.484, n = 236
Density
0.30
0.25
0.20
0.15
0.10
0.05
Predicted −LN(IC50), GDSC
Observed −LN(IC50), GDSC
AZD0530
CCLE, r = 0.515, n = 455
Density
0.6
0.5
0.4
0.3
0.2
0.1
Predicted ActArea, CCLE
Observed ActArea, CCLE
Saracatinib
Predicted DR, r = 0.637, n = 105
Density
1.0
0.5
Predicted −LN(IC50), GDSC
Predicted ActArea, CCLE

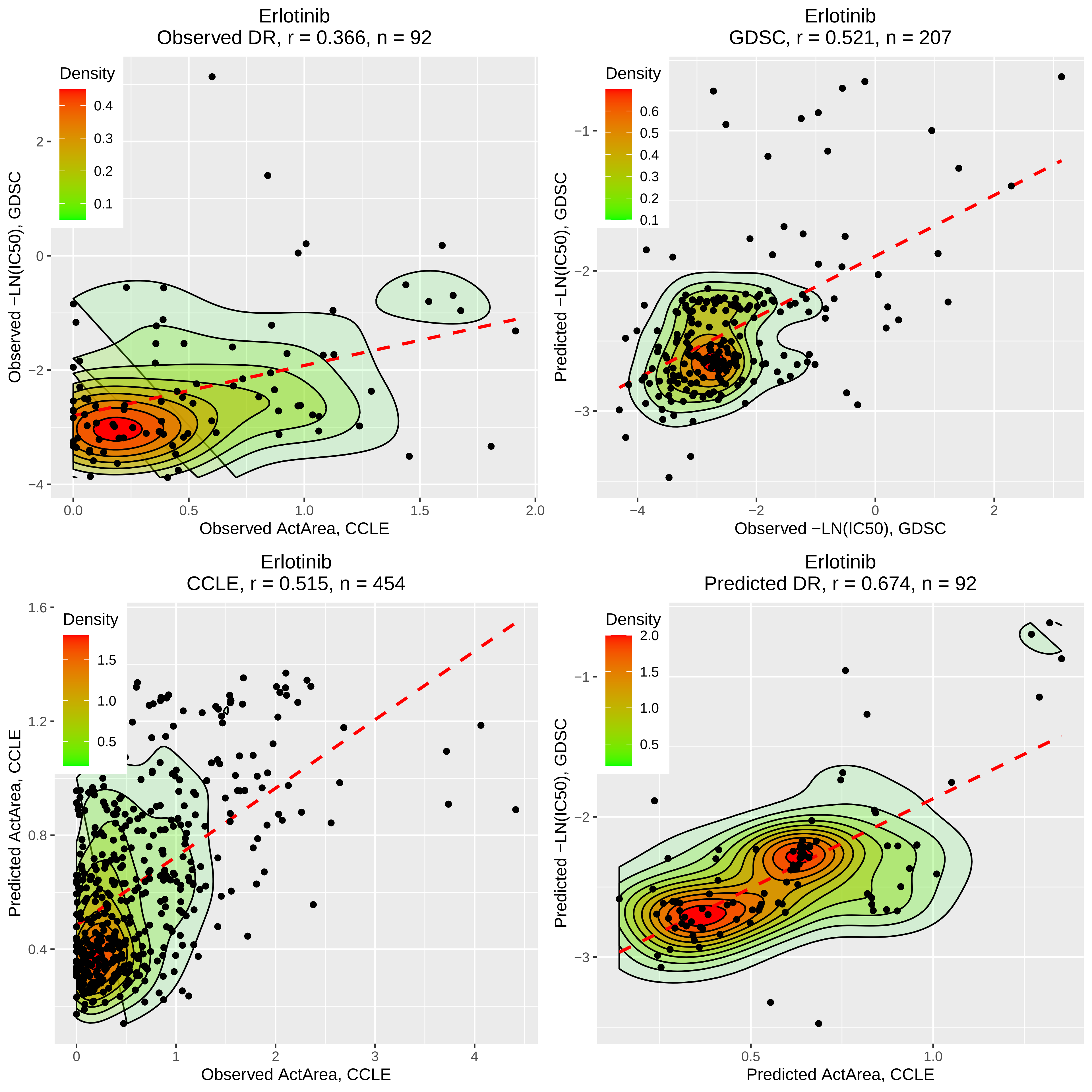

Erlotinib
Observed DR, r = 0.366, n = 92
Density
0.4
0.3
0.2
0.1
Observed −LN(IC50), GDSC
Observed ActArea, CCLE
Erlotinib
GDSC, r = 0.521, n = 207
Density
0.6
0.5
0.4
0.3
0.2
0.1
Predicted −LN(IC50), GDSC
Observed −LN(IC50), GDSC
Erlotinib
CCLE, r = 0.515, n = 454
Density
1.5
1.0
0.5
Predicted ActArea, CCLE
Observed ActArea, CCLE
Erlotinib
Predicted DR, r = 0.674, n = 92
Density
2.0
1.5
1.0
0.5
Predicted −LN(IC50), GDSC
Predicted ActArea, CCLE

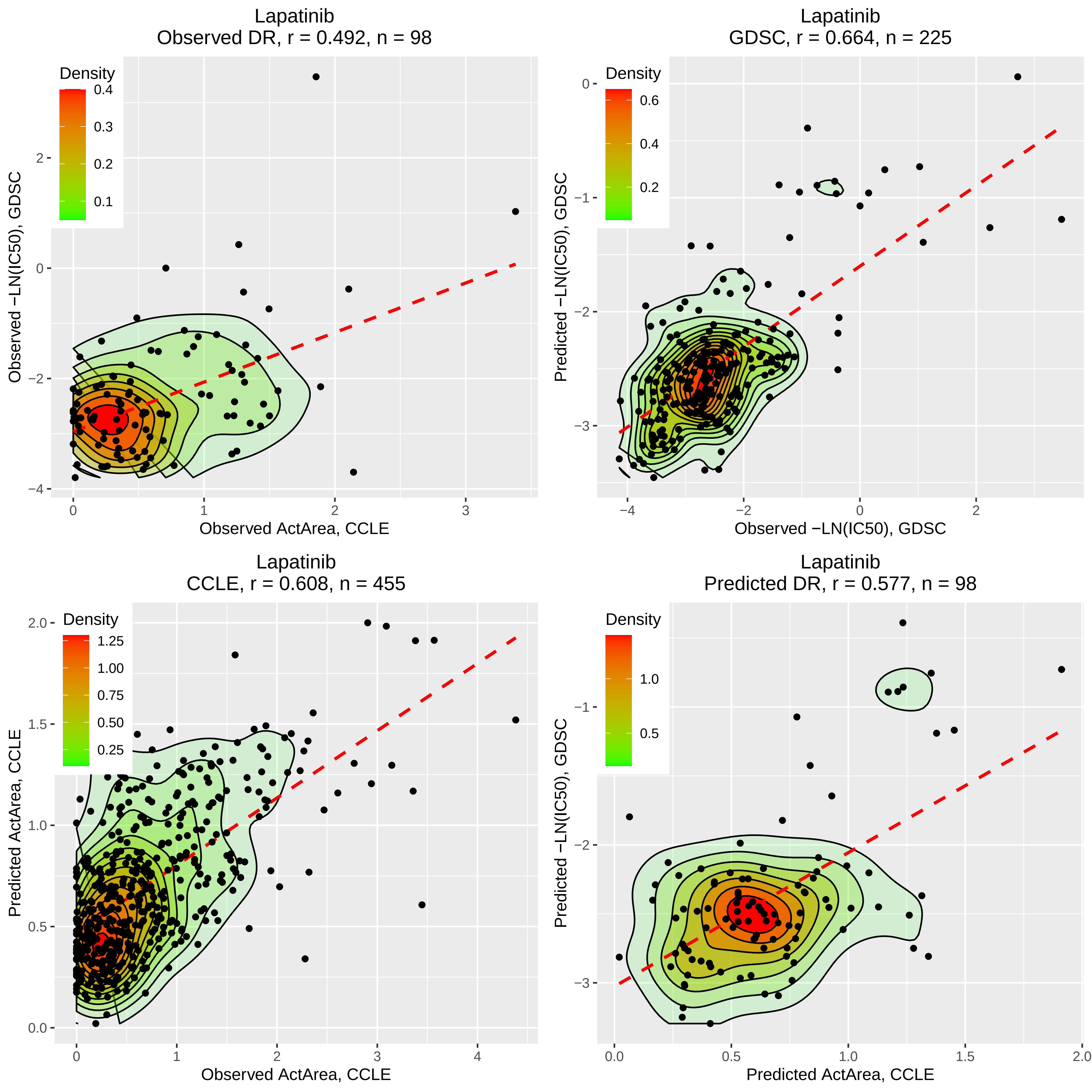

Lapatinib
Observed DR, r = 0.492, n = 98
Density
0.4
0.3
0.2
0.1
Observed −LN(IC50), GDSC
Observed ActArea, CCLE
Lapatinib
GDSC, r = 0.664, n = 225
Density
0.6
0.4
0.2
Predicted −LN(IC50), GDSC
Observed −LN(IC50), GDSC
Lapatinib
CCLE, r = 0.608, n = 455
Density
1.25
1.00
0.75
0.50
0.25
Predicted ActArea, CCLE
Observed ActArea, CCLE
Lapatinib
Predicted DR, r = 0.577, n = 98
Density
1.0
0.5
Predicted −LN(IC50), GDSC
Predicted ActArea, CCLE

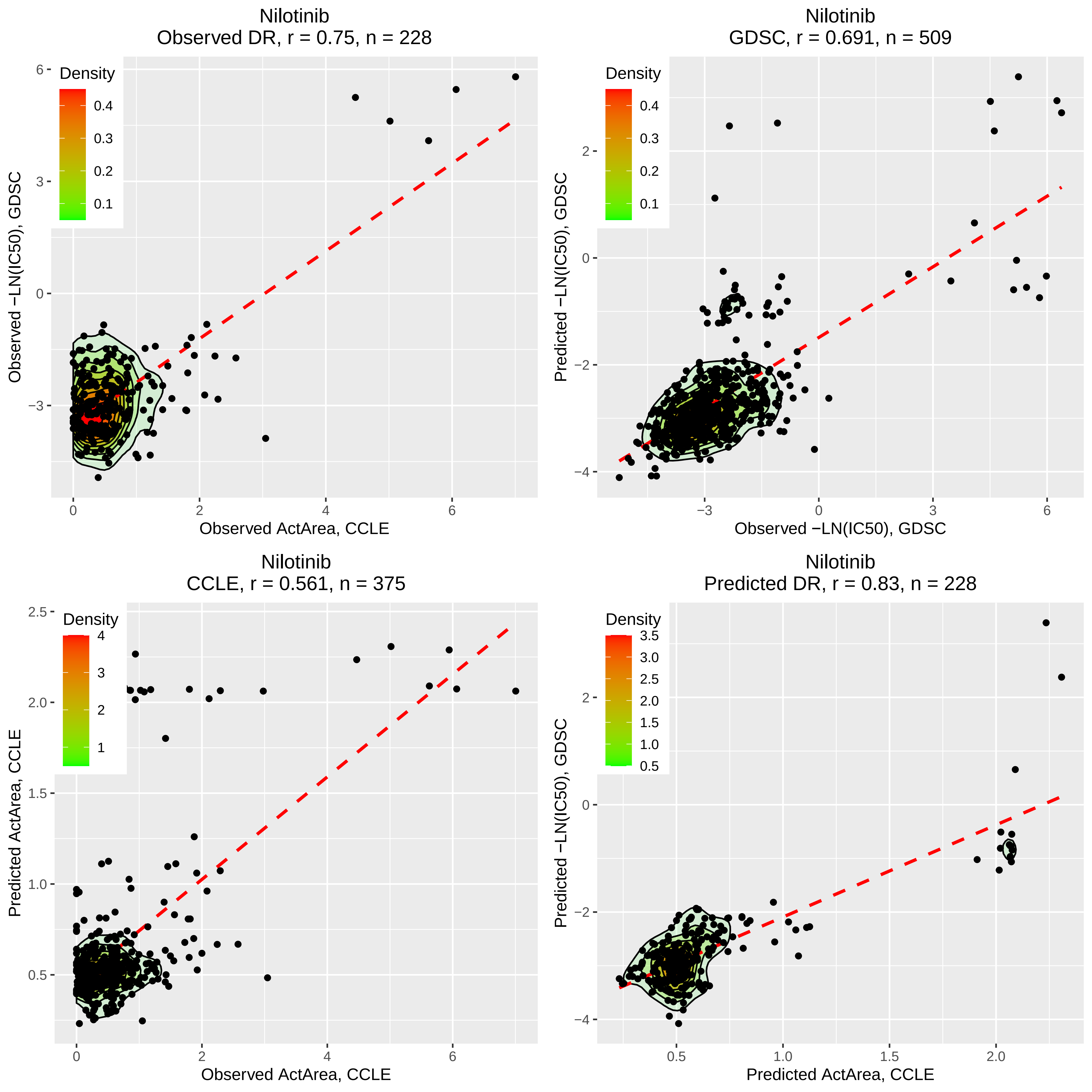

Nilotinib
Observed DR, r = 0.75, n = 228
Density
Observed −LN(IC50), GDSC
Observed ActArea, CCLE
Nilotinib
GDSC, r = 0.691, n = 509
Density
Predicted −LN(IC50), GDSC
Observed −LN(IC50), GDSC
Nilotinib
CCLE, r = 0.561, n = 375
Density
Predicted ActArea, CCLE
Observed ActArea, CCLE
Nilotinib
Predicted DR, r = 0.83, n = 228
Density
Predicted −LN(IC50), GDSC
Predicted ActArea, CCLE

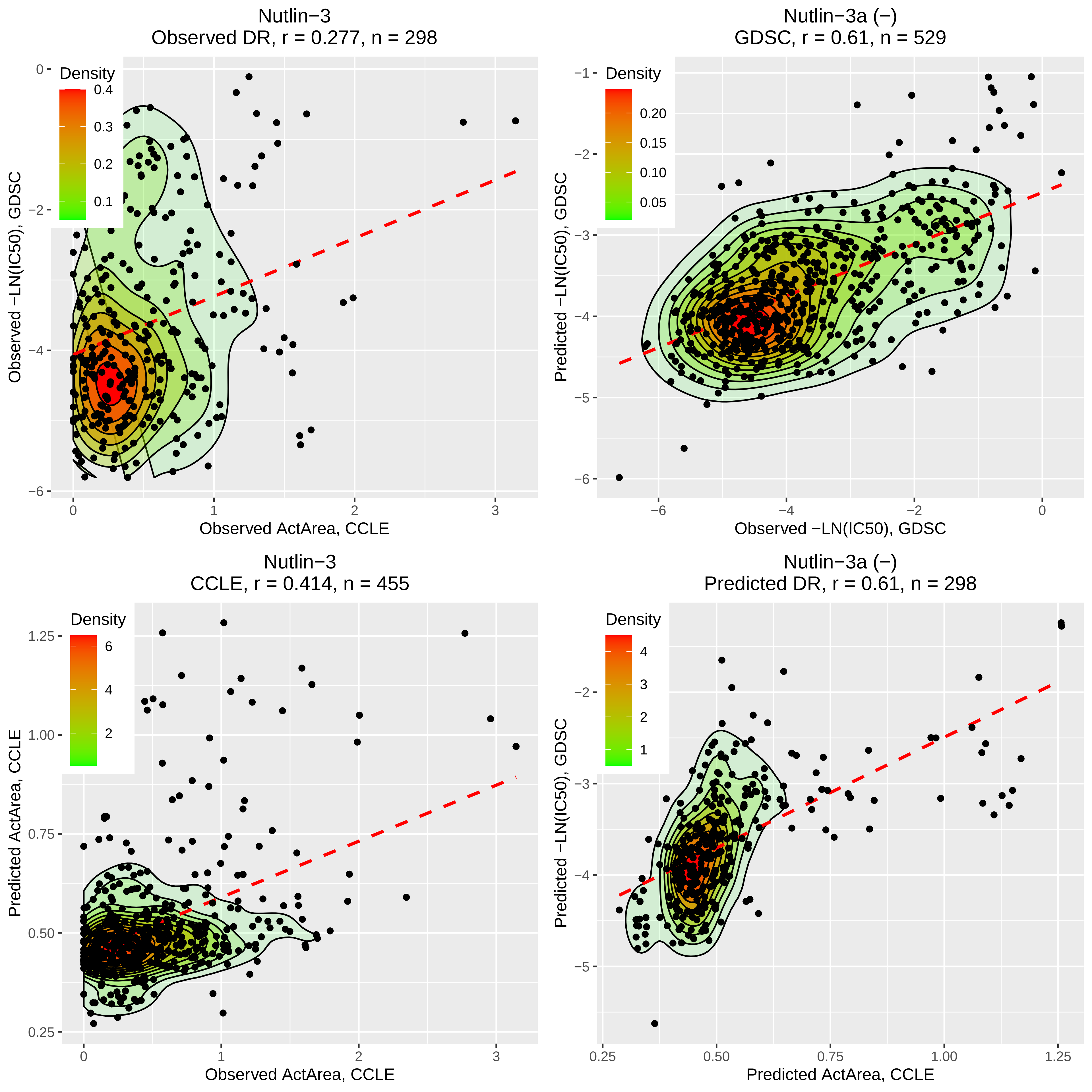

Nutlin−3
Observed DR, r = 0.277, n = 298
Density
0.4
0.3
0.2
0.1
Observed −LN(IC50), GDSC
Observed ActArea, CCLE
Nutlin−3a (−)
GDSC, r = 0.61, n = 529
Density
0.20
0.15
0.10
0.05
Predicted −LN(IC50), GDSC
Observed −LN(IC50), GDSC
Nutlin−3
CCLE, r = 0.414, n = 455
Density
6
4
2
Predicted ActArea, CCLE
Observed ActArea, CCLE
Nutlin−3a (−)
Predicted DR, r = 0.61, n = 298
Density
4
3
2
1
Predicted −LN(IC50), GDSC
Predicted ActArea, CCLE

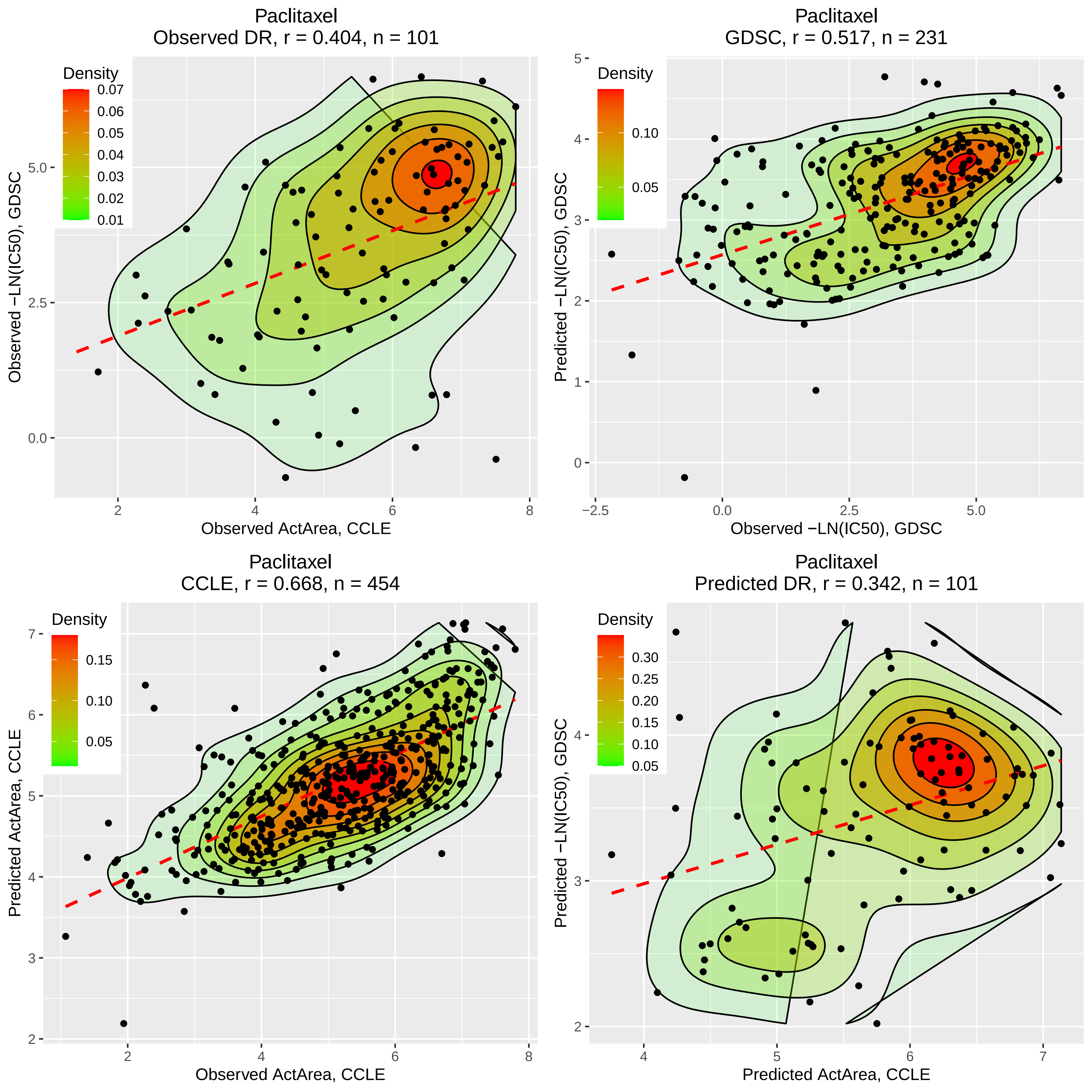

Paclitaxel
Observed DR, r = 0.404, n = 101
Density
0.07
0.06
0.05
0.04
0.03
0.02
0.01
Observed −LN(IC50), GDSC
Observed ActArea, CCLE
Paclitaxel
GDSC, r = 0.517, n = 231
Density
0.10
0.05
Predicted −LN(IC50), GDSC
Observed −LN(IC50), GDSC
Paclitaxel
CCLE, r = 0.668, n = 454
Density
0.15
0.10
0.05
Predicted ActArea, CCLE
Observed ActArea, CCLE
Paclitaxel
Predicted DR, r = 0.342, n = 101
Density
0.30
0.25
0.20
0.15
0.10
0.05
Predicted −LN(IC50), GDSC
Predicted ActArea, CCLE

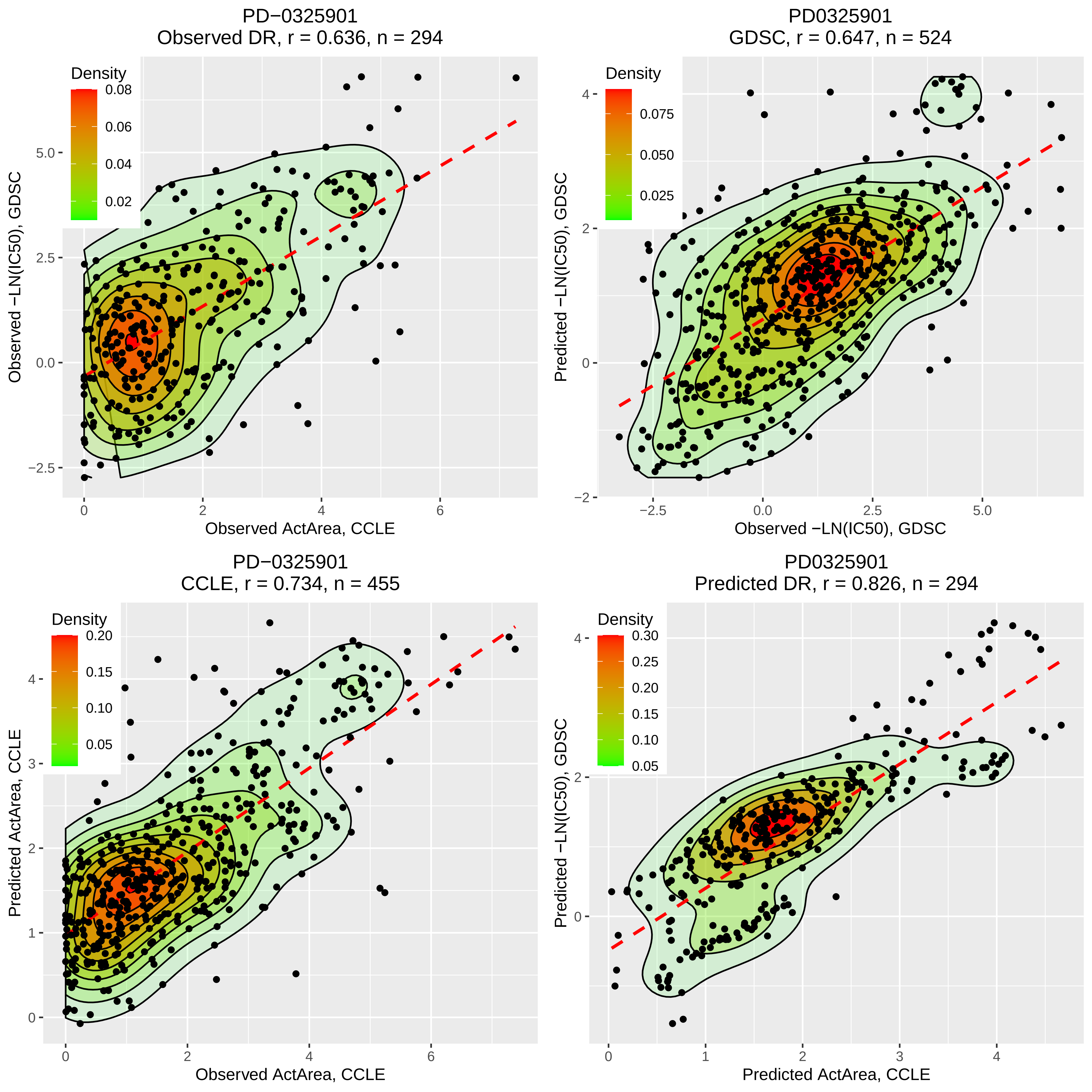
PD−0325901
Observed DR, r = 0.636, n = 294
Density
0.08
0.06
0.04
0.02
Observed −LN(IC50), GDSC
Observed ActArea, CCLE
PD0325901
GDSC, r = 0.647, n = 524
Density
0.075
0.050
0.025
Predicted −LN(IC50), GDSC
Observed −LN(IC50), GDSC
PD−0325901
CCLE, r = 0.734, n = 455
Density
0.20
0.15
0.10
0.05
Predicted ActArea, CCLE
Observed ActArea, CCLE
PD0325901
Predicted DR, r = 0.826, n = 294
Density
0.30
0.25
0.20
0.15
0.10
0.05
Predicted −LN(IC50), GDSC
Predicted ActArea, CCLE

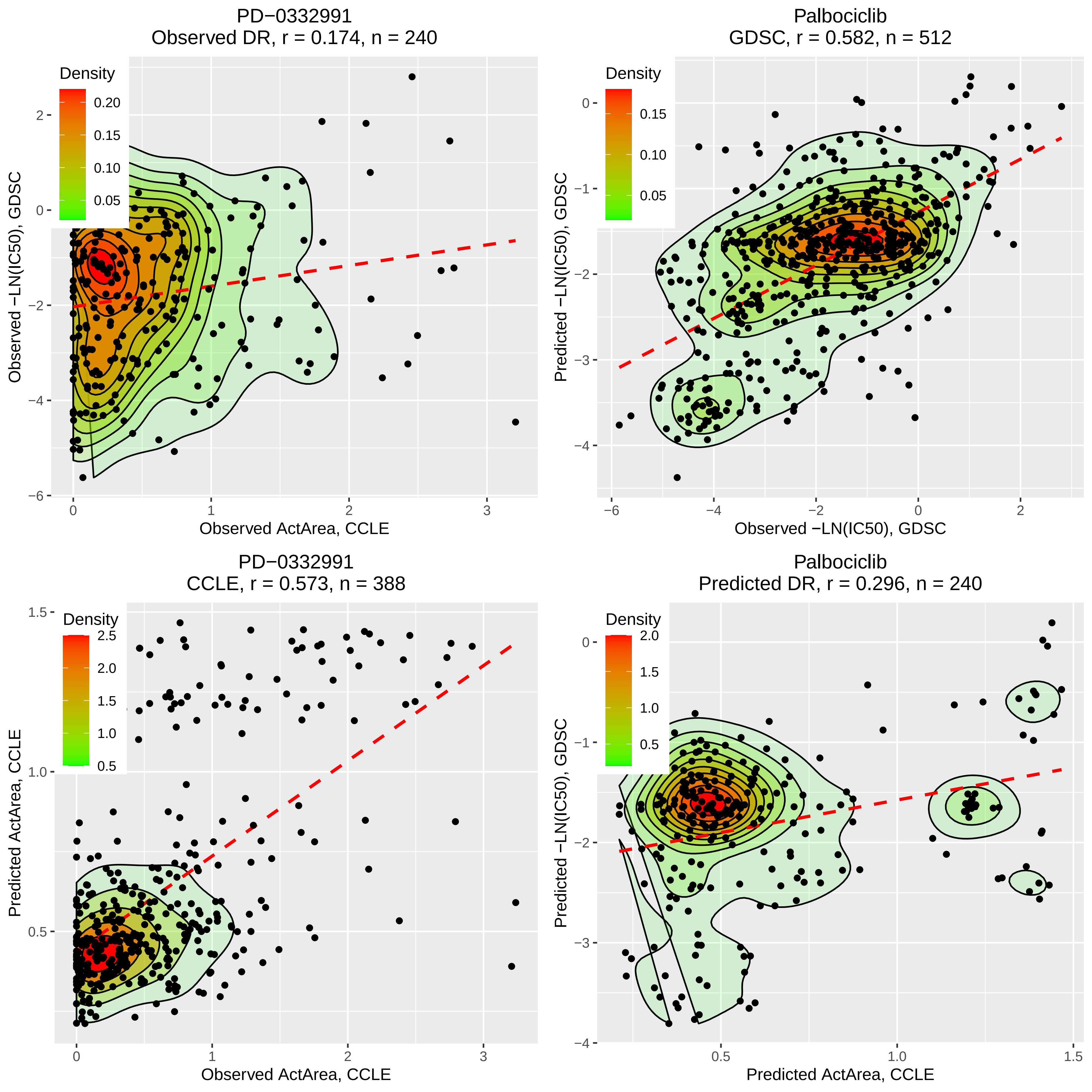

PD−0332991
Observed DR, r = 0.174, n = 240
Density
0.20
0.15
0.10
0.05
Observed −LN(IC50), GDSC
Observed ActArea, CCLE
Palbociclib
GDSC, r = 0.582, n = 512
Density
0.15
0.10
0.05
Predicted −LN(IC50), GDSC
Observed −LN(IC50), GDSC
PD−0332991
CCLE, r = 0.573, n = 388
Density
2.5
2.0
1.5
1.0
0.5
Predicted ActArea, CCLE
Observed ActArea, CCLE
Palbociclib
Predicted DR, r = 0.296, n = 240
Density
2.0
1.5
1.0
0.5
Predicted −LN(IC50), GDSC
Predicted ActArea, CCLE

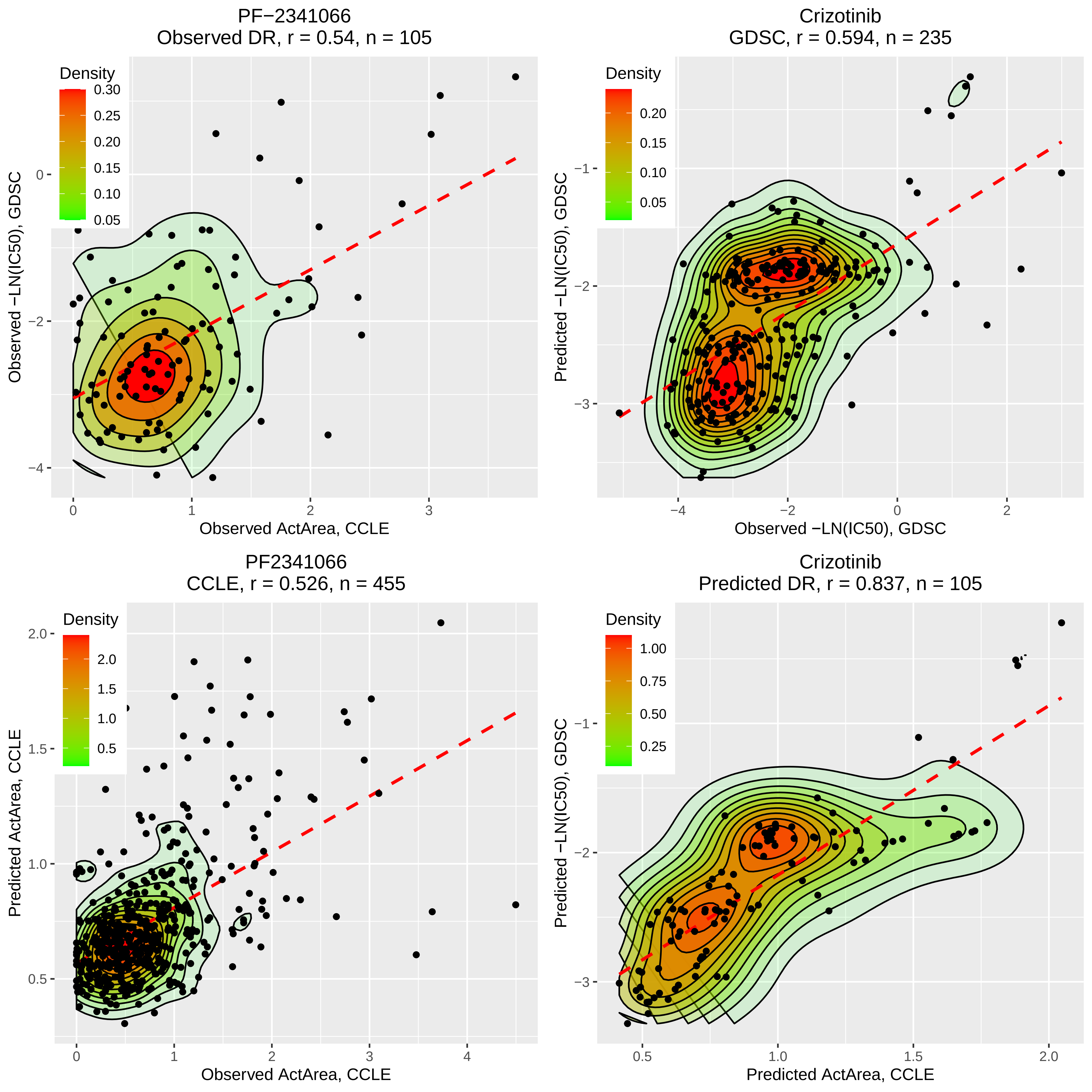

PF−2341066
Observed DR, r = 0.54, n = 105
Density
0.30
0.25
0.20
0.15
0.10
0.05
Observed −LN(IC50), GDSC
Observed ActArea, CCLE
Crizotinib
GDSC, r = 0.594, n = 235
Density
0.20
0.15
0.10
0.05
Predicted −LN(IC50), GDSC
Observed −LN(IC50), GDSC
PF2341066
CCLE, r = 0.526, n = 455
Density
2.0
1.5
1.0
0.5
Predicted ActArea, CCLE
Observed ActArea, CCLE
Crizotinib
Predicted DR, r = 0.837, n = 105
Density
1.00
0.75
0.50
0.25
Predicted −LN(IC50), GDSC
Predicted ActArea, CCLE

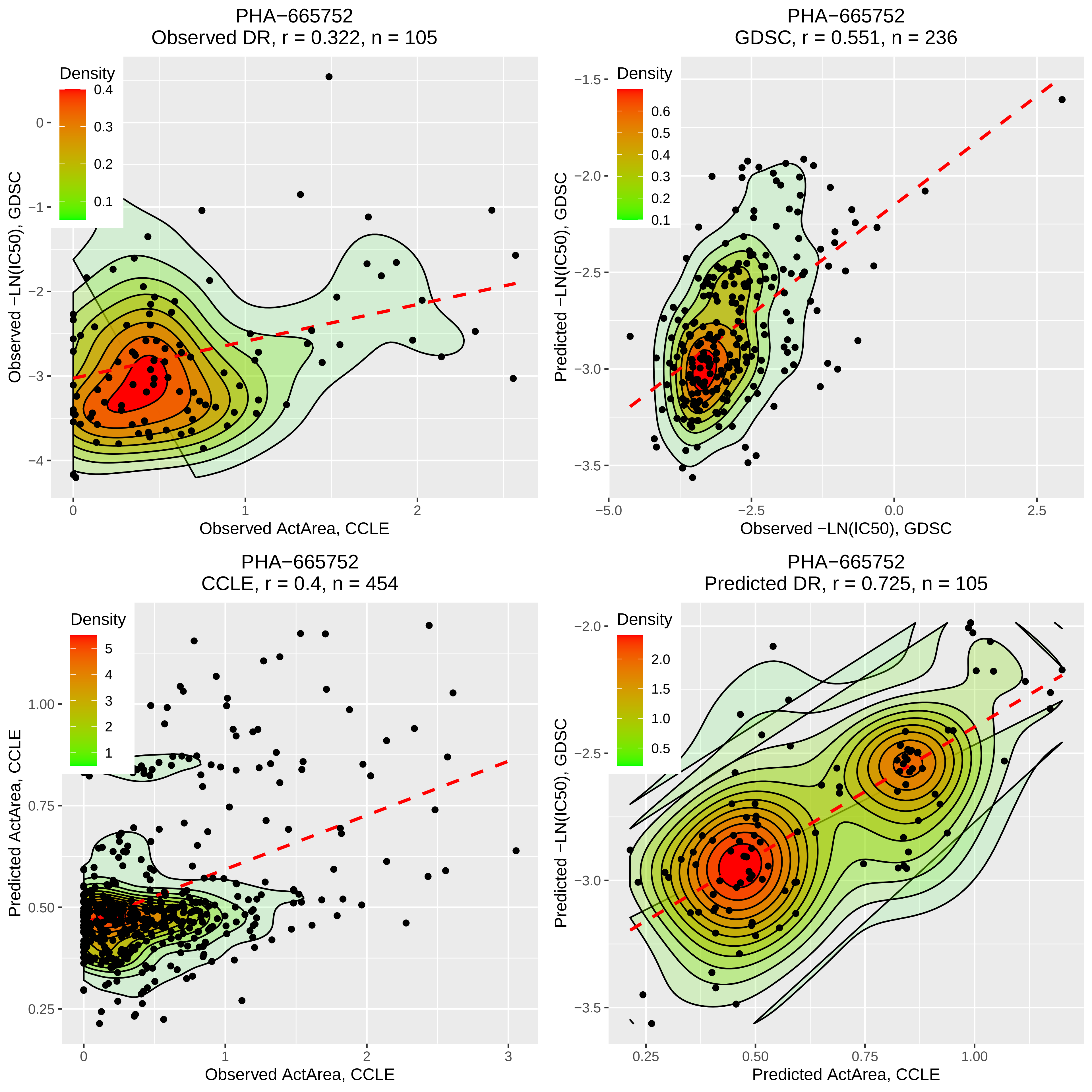

PHA−665752
Observed DR, r = 0.322, n = 105
Density
0.4
0.3
0.2
0.1
0
−1
−2
−3
−4
Observed −LN(IC50), GDSC
0
1
2
Observed ActArea, CCLE
PHA−665752
GDSC, r = 0.551, n = 236
Density
0.6
0.5
0.4
0.3
0.2
0.1
−1.5
−2.0
−2.5
−3.0
−3.5
Predicted −LN(IC50), GDSC
−5.0
−2.5
0.0
2.5
Observed −LN(IC50), GDSC
PHA−665752
CCLE, r = 0.4, n = 454
Density
5
4
3
2
1
1.00
0.75
0.50
0.25
Predicted ActArea, CCLE
0
1
2
3
Observed ActArea, CCLE
PHA−665752
Predicted DR, r = 0.725, n = 105
Density
2.0
1.5
1.0
0.5
−2.0
−2.5
−3.0
−3.5
Predicted −LN(IC50), GDSC
0.25
0.50
0.75
1.00
Predicted ActArea, CCLE

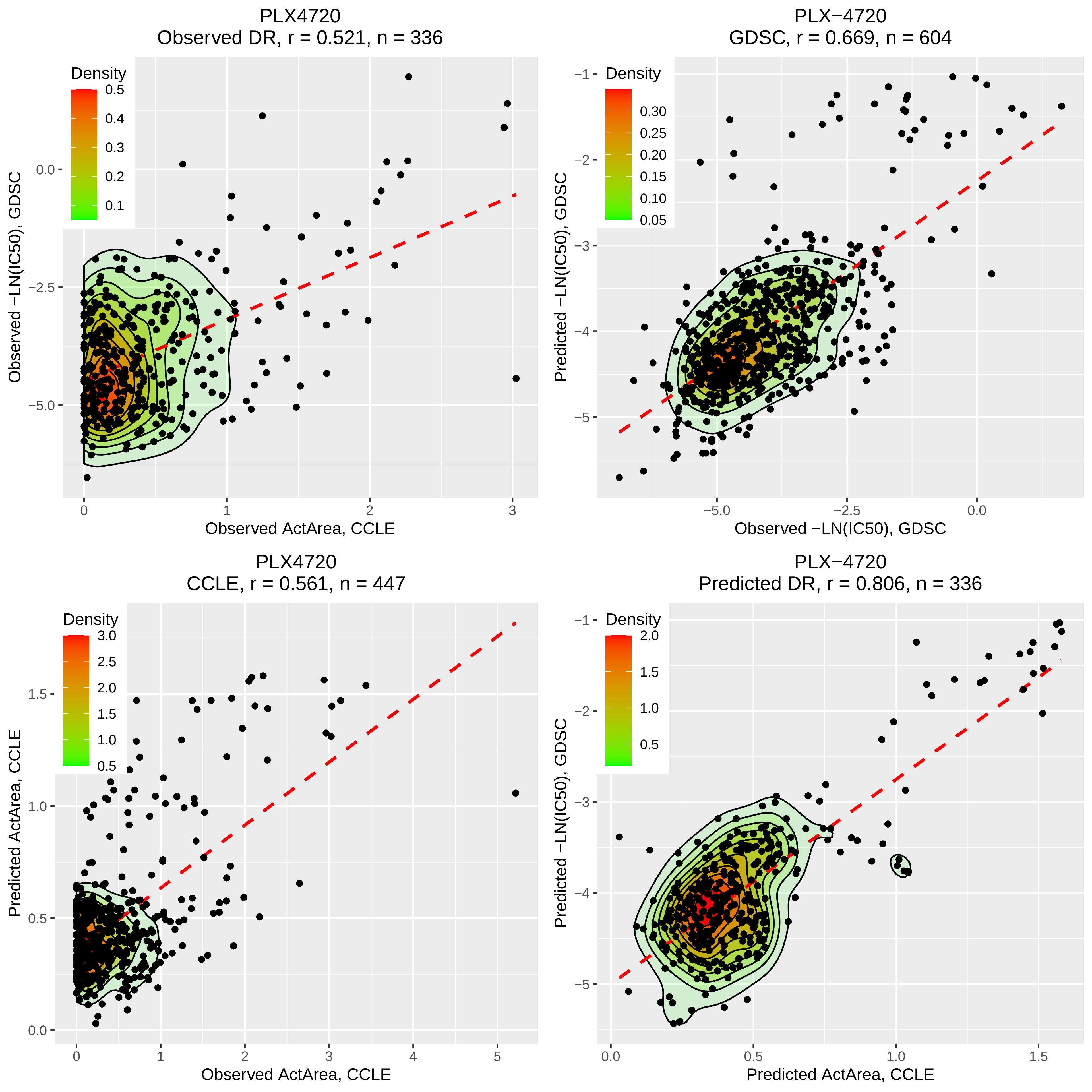
PLX4720
Observed DR, r = 0.521, n = 336
Observed ActArea, CCLE
Observed −LN(IC50), GDSC
Density
PLX−4720
GDSC, r = 0.669, n = 604
Observed −LN(IC50), GDSC
Predicted −LN(IC50), GDSC
PLX4720
CCLE, r = 0.561, n = 447
Observed ActArea, CCLE
Predicted ActArea, CCLE
PLX−4720
Predicted DR, r = 0.806, n = 336
Predicted ActArea, CCLE
Predicted −LN(IC50), GDSC

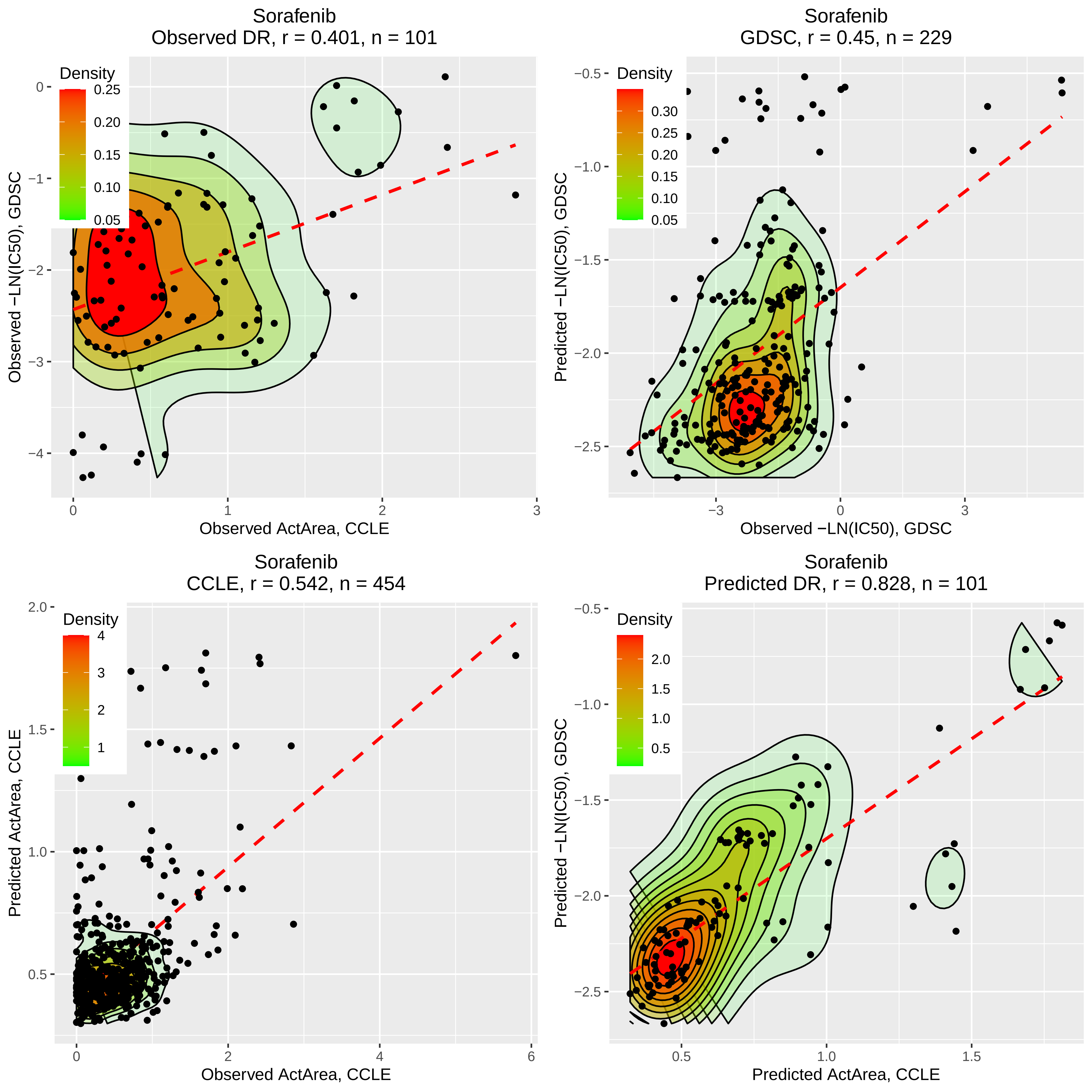

Sorafenib
Observed DR, r = 0.401, n = 101
Density
0.25
0.20
0.15
0.10
0.05
Observed −LN(IC50), GDSC
Observed ActArea, CCLE
Sorafenib
GDSC, r = 0.45, n = 229
Density
0.30
0.25
0.20
0.15
0.10
0.05
Predicted −LN(IC50), GDSC
Observed −LN(IC50), GDSC
Sorafenib
CCLE, r = 0.542, n = 454
Density
4
3
2
1
Predicted ActArea, CCLE
Observed ActArea, CCLE
Sorafenib
Predicted DR, r = 0.828, n = 101
Density
2.0
1.5
1.0
0.5
Predicted −LN(IC50), GDSC
Predicted ActArea, CCLE

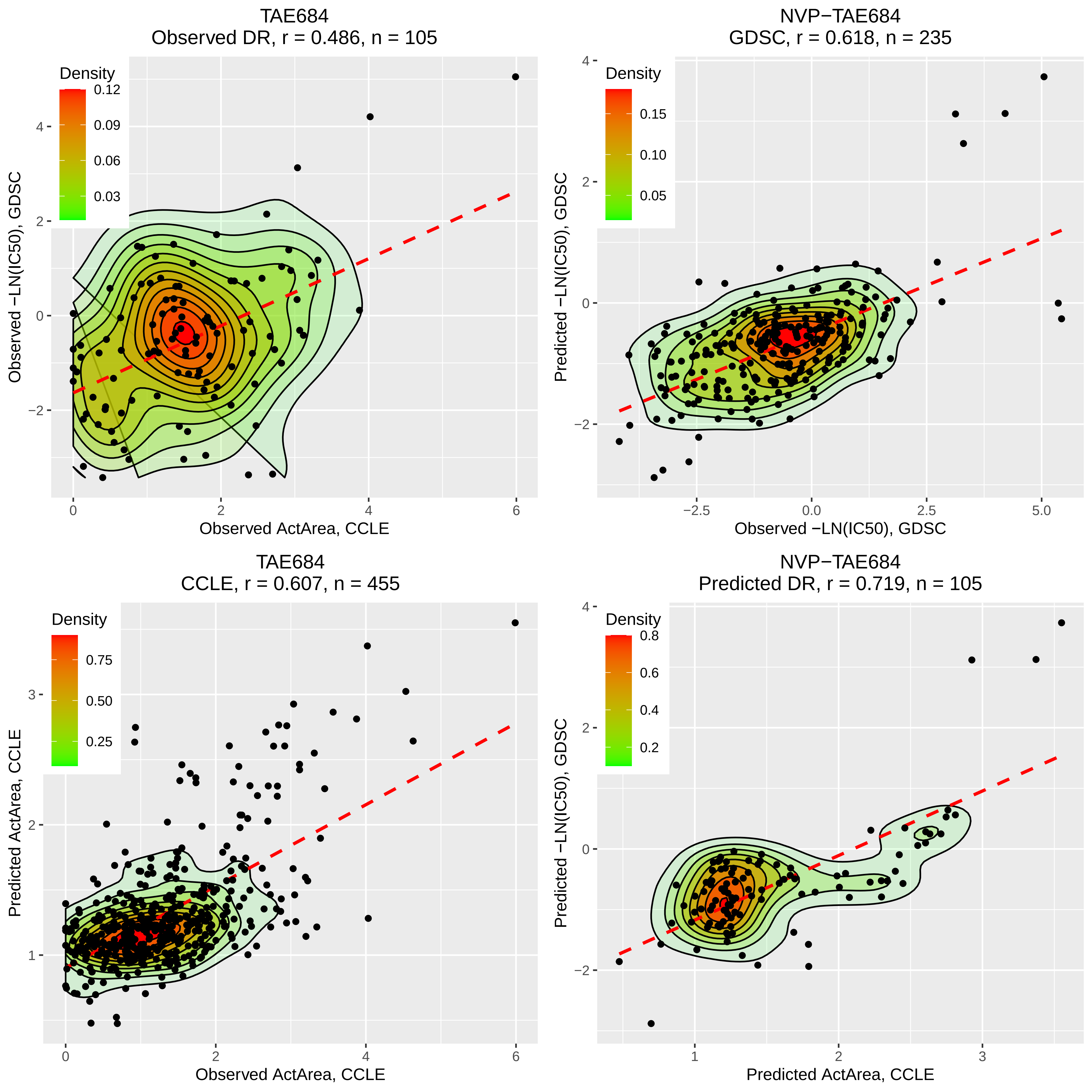

TAE684
Observed DR, r = 0.486, n = 105
Density
Observed −LN(IC50), GDSC
Observed ActArea, CCLE
NVP−TAE684
GDSC, r = 0.618, n = 235
Predicted −LN(IC50), GDSC
Observed −LN(IC50), GDSC
TAE684
CCLE, r = 0.607, n = 455
Predicted ActArea, CCLE
Observed ActArea, CCLE
NVP−TAE684
Predicted DR, r = 0.719, n = 105
Predicted −LN(IC50), GDSC
Predicted ActArea, CCLE

Supplementary Figure 3. Comparison of the observed and predicted drug response for 14 shared drugs between CCLE and GDSC.

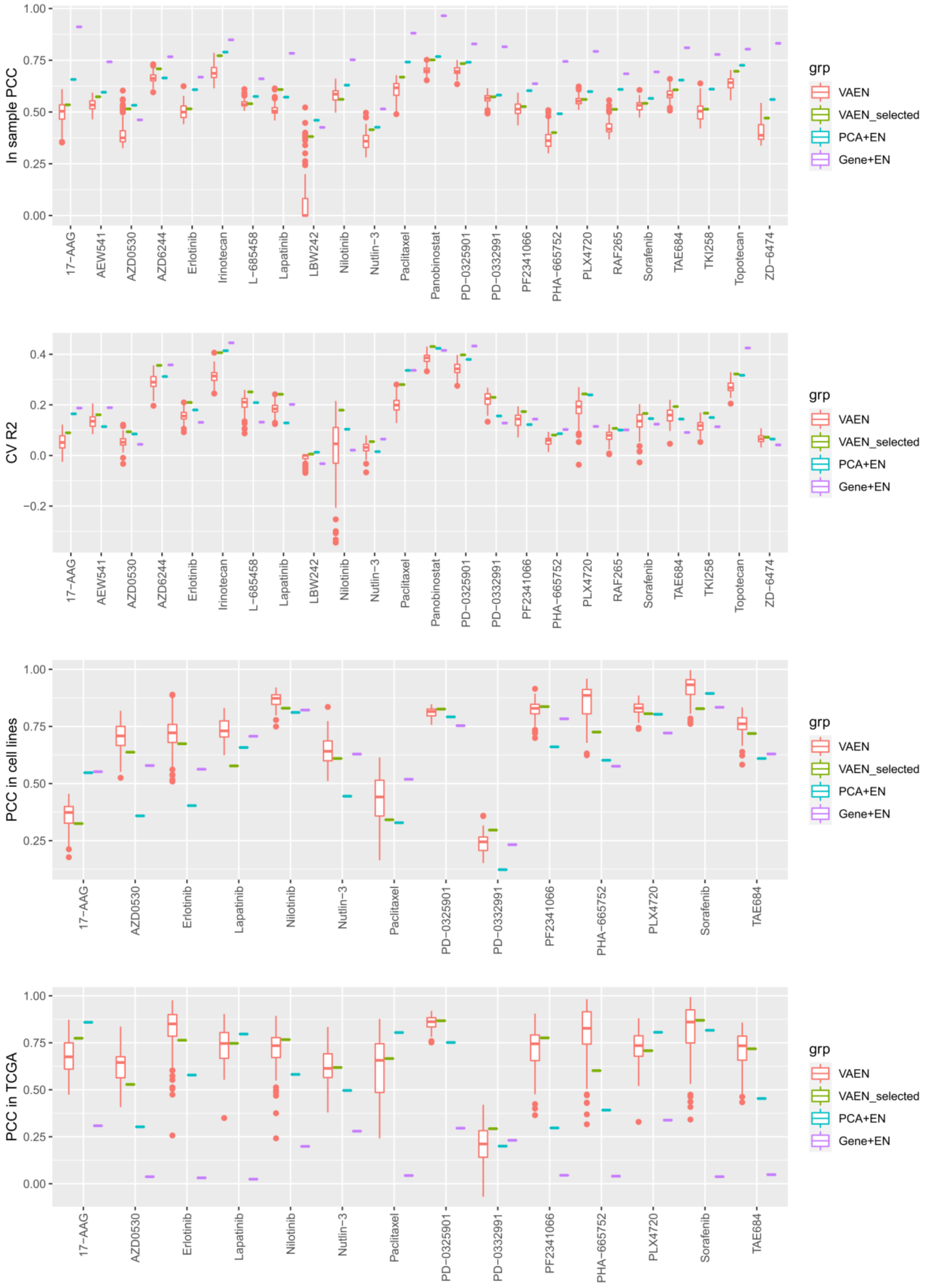

Supplementary Figure 4. Model comparison and evaluation using computational measurements. (A-B) Comparison of models using in-sample PCC (A) and holdout R2 (B). (C-D) Cross-panel model comparison using shared drugs in CCLE cell line data (C) and TCGA (D). Each box shows the inter quartile range (IQR between Q1 and Q3) for the corresponding set. The central mark (horizontal line) shows the median and the whiskers show the rest of the distribution based on IQR [Q1-1.5×IQR, Q3+1.5×IQR]. Data outside of this range are considered outliers and represented by solid dots.

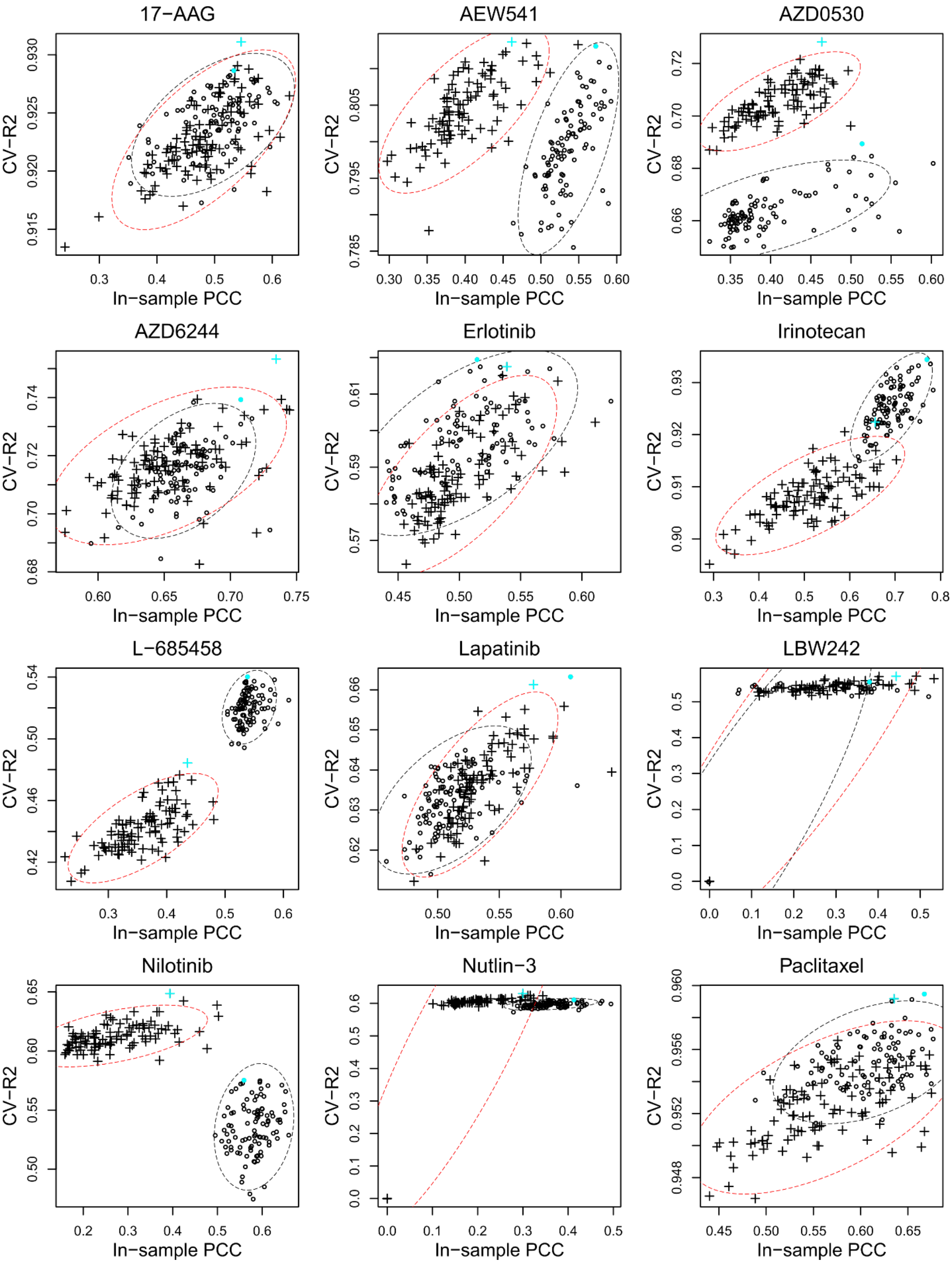
17−AAG
AEW541
AZD0530
AZD6244
Erlotinib
Irinotecan
L−685458
Lapatinib
LBW242
Nilotinib
Nutlin−3
Paclitaxel
CV−R2
In−sample PCC

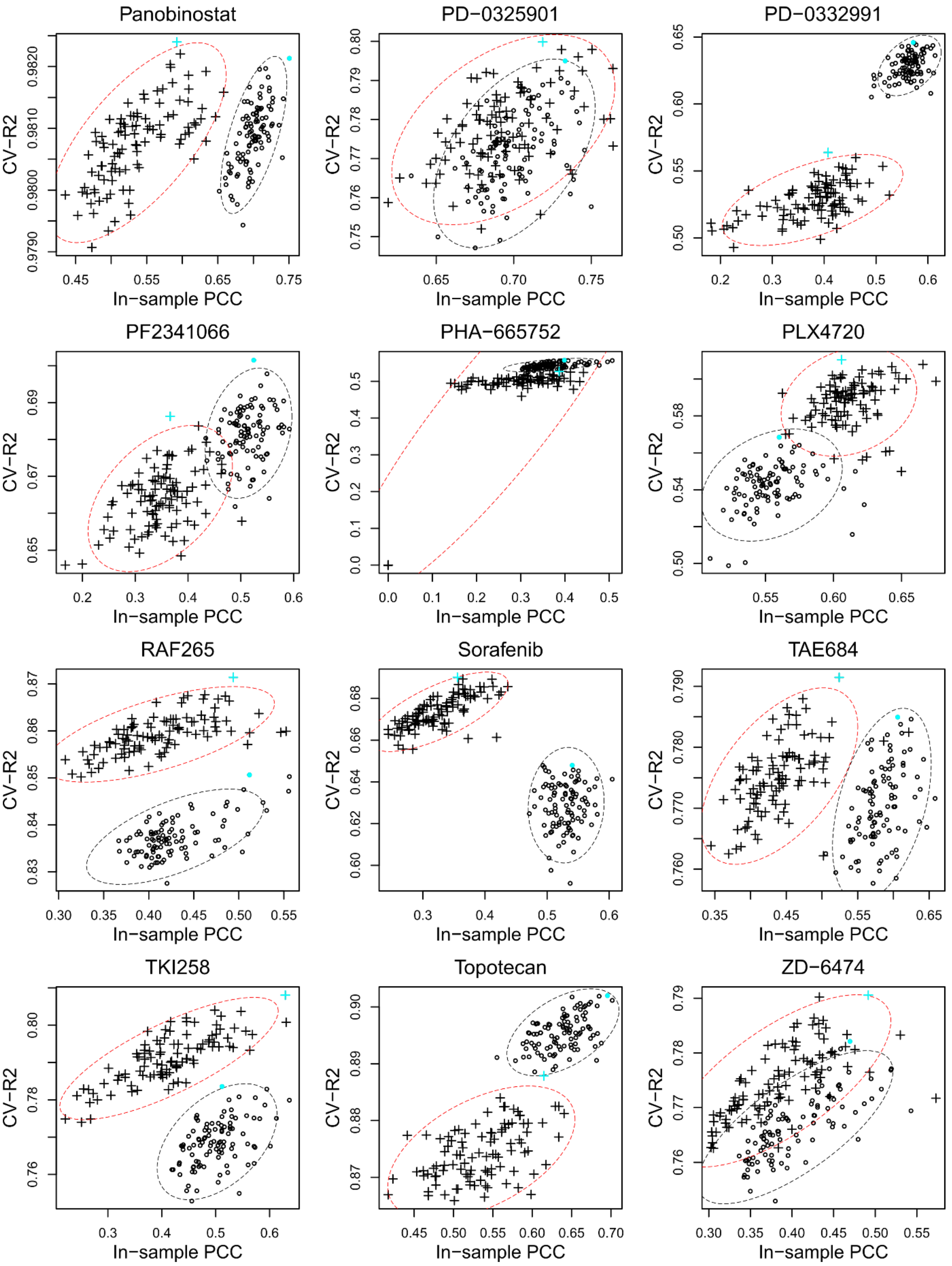

Supplementary Figure 5. Performance comparison for A-models and S-models. Dots in the circle shape: parameters from A-models; dots in the plus (+) shape: parameters from S-models. The dots in cyan: the best models from the A-model (circle) and the S-model (+) respectively. The black (red) dash eclipse: the 95 percent confidence interval of the A-models (S-models).

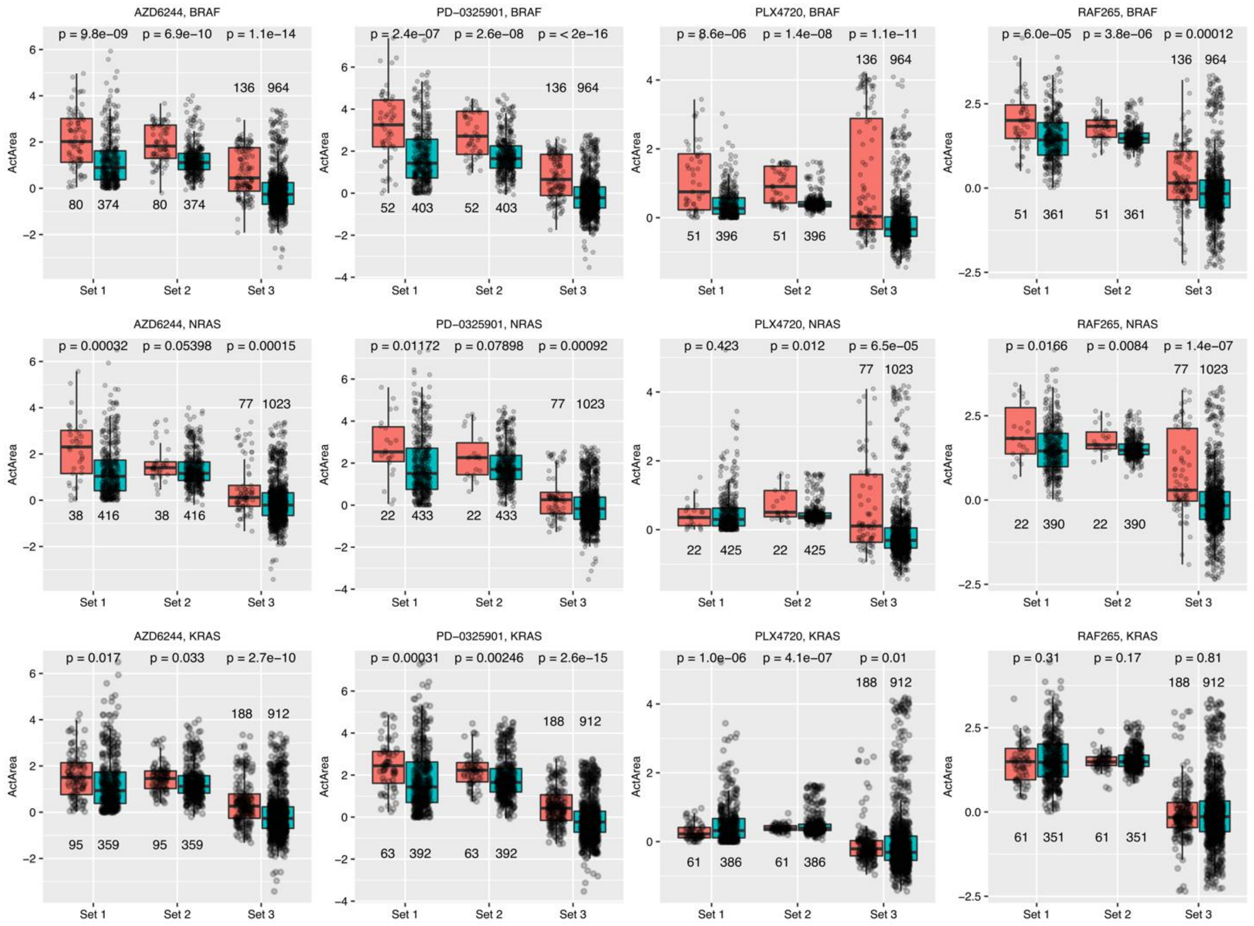

Supplementary Figure 6. Comparison of drug response in mutant (red) samples versus wild type (blue) samples for three genes (*BRAF*, *NRAS*, and *KRAS*) and four drugs (two MEK inhibitors: AZD6244 and PD-0325901; and two BRAF inhibitors: PLX4720 and RAF265). The drug response was from three sets: set 1: the observed response; set 2: the predicted response in the same cell lines as in set 1; and set 3: the imputed response in all 1100 cell lines. The sample size for each group is annotated above or below the box. A two-sided *t*-test was conducted for each set between the mutant samples and wild type samples. Each box shows the inter quartile range (IQR between Q1 and Q3) for the corresponding set. The central mark (horizontal line) shows the median and the whiskers show the rest of the distribution based on IQR [Q1-1.5×IQR, Q3+1.5×IQR].

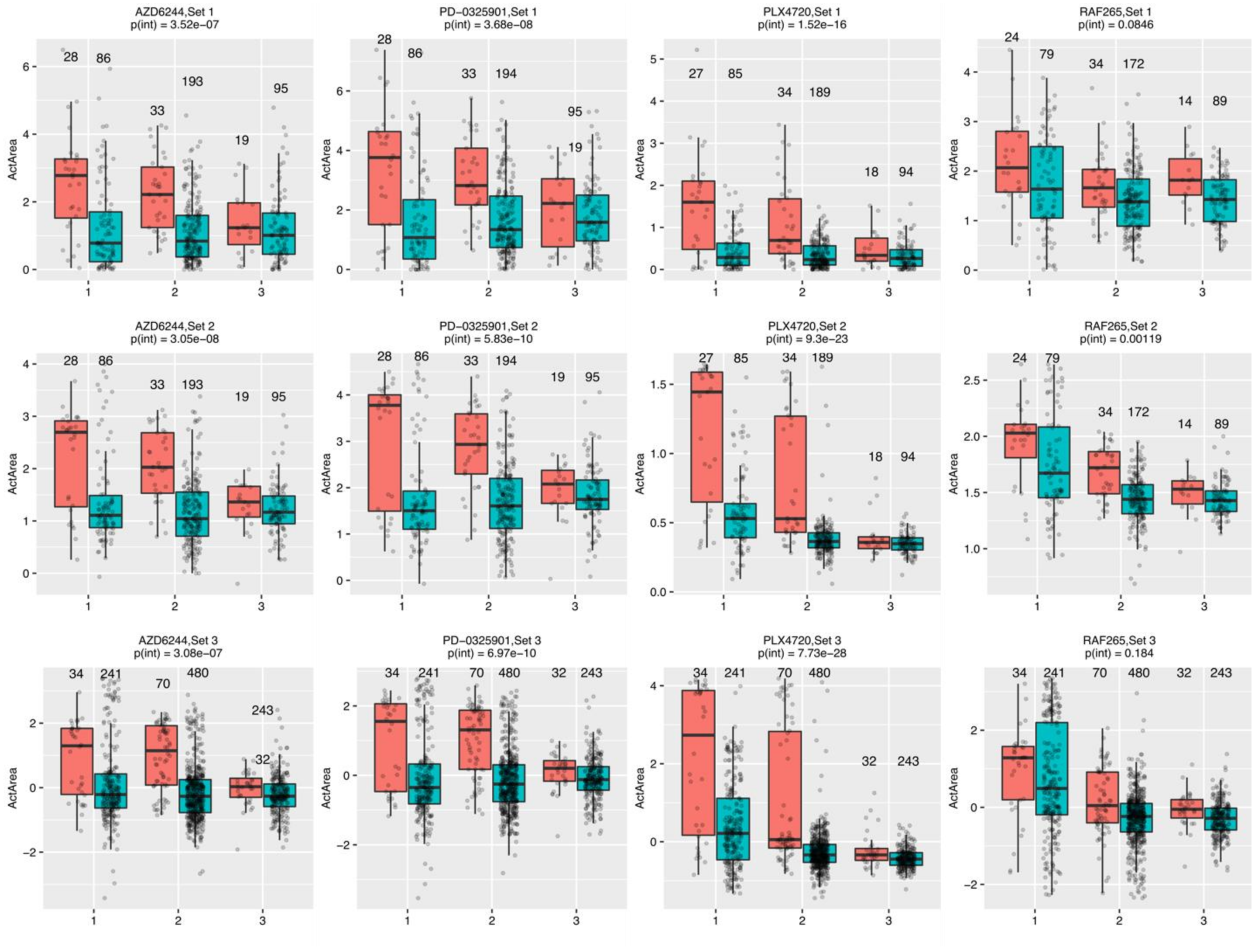

Supplementary Figure 7. Demonstration of the interaction effect between *BRAF* mutations and *EGFR* expression in three sets. In each panel, a regression function was fitted following $\mathbf{Y}_{\text{response}} \sim \mathbf{X}_{BRAF} + \mathbf{X}_{EGFR} + \mathbf{X}_{BRAF} \times \mathbf{X}_{EGFR}$, where $\mathbf{X}_{BRAF}$ indicated whether a sample harbored a *BRAF* mutation (red: mutant; blue: wild type) and X_{EGFR} was a factor with three levels indicating group information of a sample defined by *EGFR* gene expression. We defined three groups according to *EGFR* gene expression (x-axis): the lower quarter (group 1), the middle half (group 2), and the higher quarter of samples (group 3). *P*-value for the interaction factor was shown in the title. The sample size for each group is annotated above the box. The *p*-value was obtained from the fitted regression model for the interaction effect $\mathbf{X}_{BRAF} \times \mathbf{X}_{EGFR}$. Each box shows the inter quartile range (IQR between Q1 and Q3) for the corresponding set. The central mark (horizontal line) shows the median and the whiskers show the rest of the distribution based on IQR [Q1-1.5×IQR, Q3+1.5×IQR].

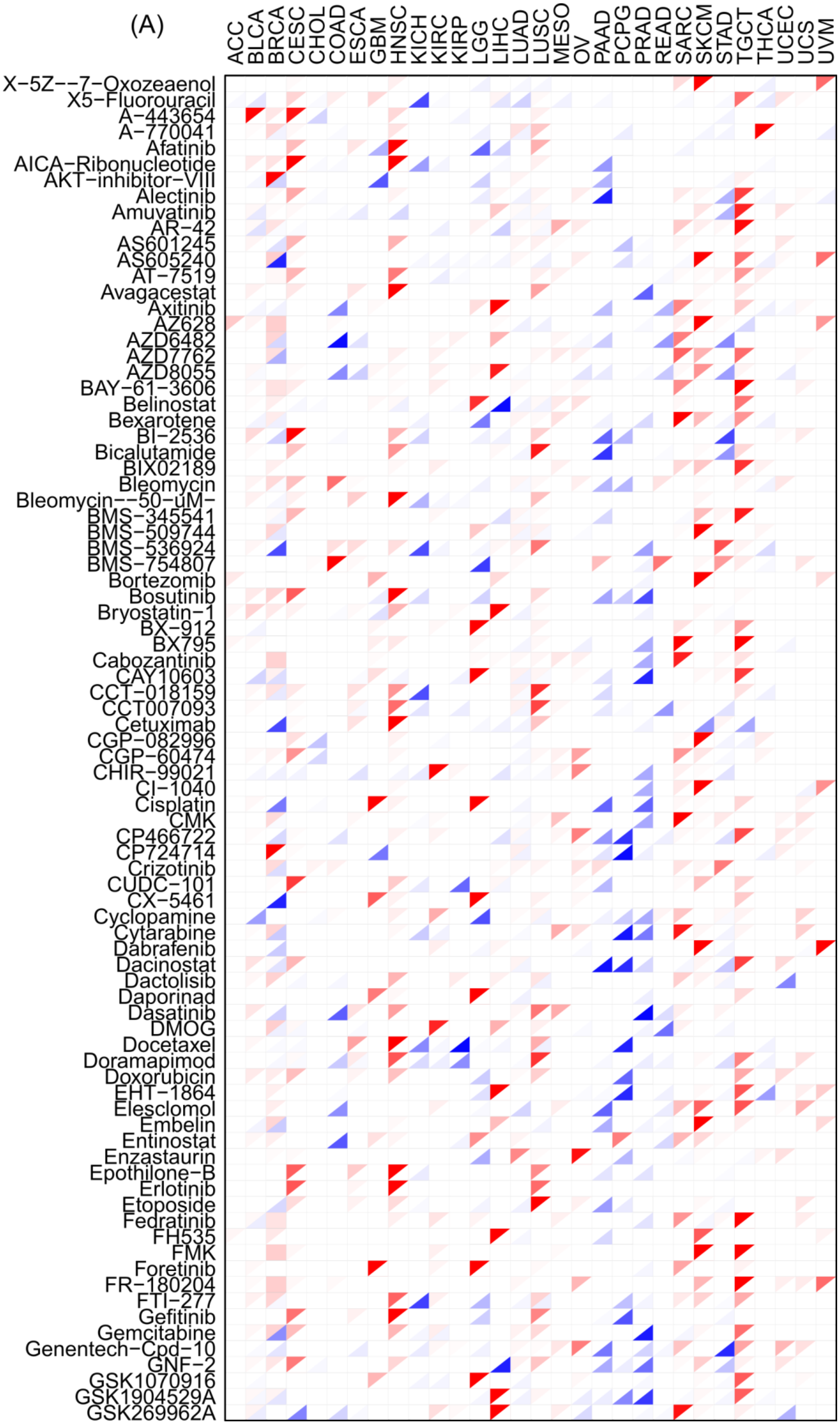
(A)
ACC
BLCA
BRCA
CESC
CHOL
COAD
ESCA
GBM
HNSC
KICH
KIRC
KIRP
LGG
LIHC
LUAD
LUSC
MESO
OV
PAAD
PCPG
PRAD
READ
SARC
SKCM
STAD
TGCT
THCA
UCEC
UCS
UVM
X-5Z--7-Oxozeaenol
X5-Fluorouracil
A-443654
A-770041
Afatinib
AICA-Ribonucleotide
AKT-inhibitor-VIII
Alectinib
Amuvatinib
AR-42
AS601245
AS605240
AT-7519
Avagacestat
Axitinib
AZ628
AZD6482
AZD7762
AZD8055
BAY-61-3606
Belinostat
Bexarotene
BI-2536
Bicalutamide
BIX02189
Bleomycin
Bleomycin--50-uM-
BMS-345541
BMS-509744
BMS-536924
BMS-754807
Bortezomib
Bosutinib
Bryostatin-1
BX-912
BX795
Cabozantinib
CAY10603
CCT-018159
CCT007093
Cetuximab
CGP-082996
CGP-60474
CHIR-99021
CI-1040
Cisplatin
CMK
CP466722
CP724714
Crizotinib
CUDC-101
CX-5461
Cyclopamine
Cytarabine
Dabrafenib
Dacinostat
Dactolisib
Daporinad
Dasatinib
DMOG
Docetaxel
Doramapimod
Doxorubicin
EHT-1864
Elesclomol
Embelin
Entinostat
Enzastaurin
Epothilone-B
Erlotinib
Etoposide
Fedratinib
FH535
FMK
Foretinib
FR-180204
FTI-277
Gefitinib
Gemcitabine
Genentech-Cpd-10
GNF-2
GSK1070916
GSK1904529A
GSK269962A

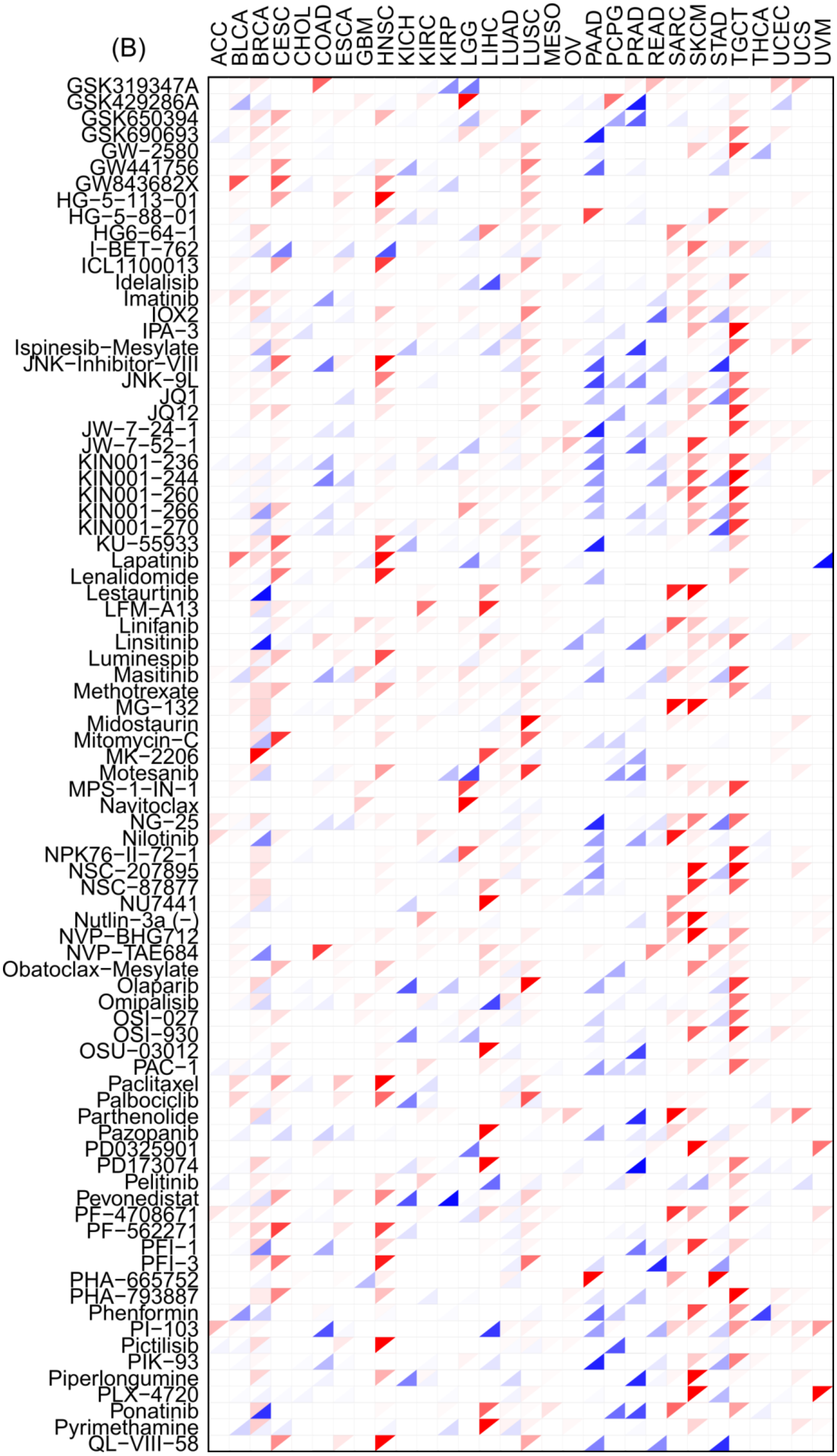
(B)
ACC
BLCA
BRCA
CESC
CHOL
COAD
ESCA
GBM
HNSC
KICH
KIRC
KIRP
LGG
LIHC
LUAD
LUSC
MESO
OV
PAAD
PCPG
PRAD
READ
SARC
SKCM
STAD
TGCT
THCA
UCEC
UCS
UVM
GSK319347A
GSK429286A
GSK650394
GSK690693
GW-2580
GW441756
GW843682X
HG-5-113-01
HG-5-88-01
HG6-64-1
I-BET-762
ICL1100013
Idelalisib
Imatinib
IOX2
IPA-3
Ispinesib-Mesylate
JNK-Inhibitor-VIII
JNK-9L
JQ1
JQ12
JW-7-24-1
JW-7-52-1
KIN001-236
KIN001-244
KIN001-260
KIN001-266
KIN001-270
KU-55933
Lapatinib
Lenalidomide
Lestaurtinib
LFM-A13
Linifanib
Linsitinib
Luminespib
Masitinib
Methotrexate
MG-132
Midostaurin
Mitomycin-C
MK-2206
Motesanib
MPS-1-IN-1
Navitoclax
NG-25
Nilotinib
NPK76-II-72-1
NSC-207895
NSC-87877
NU7441
Nutlin-3a (-)
NVP-BHG712
NVP-TAE684
Obatoclax-Mesylate
Olaparib
Omipalisib
OSI-027
OSI-930
OSU-03012
PAC-1
Paclitaxel
Palbociclib
Parthenolide
Pazopanib
PD0325901
PD173074
Pelitinib
Pevonedistat
PF-4708671
PF-562271
PFI-1
PFI-3
PHA-665752
PHA-793887
Phenformin
PI-103
Pictilisib
PIK-93
Piperlongumine
PLX-4720
Ponatinib
Pyrimethamine
QL-VIII-58

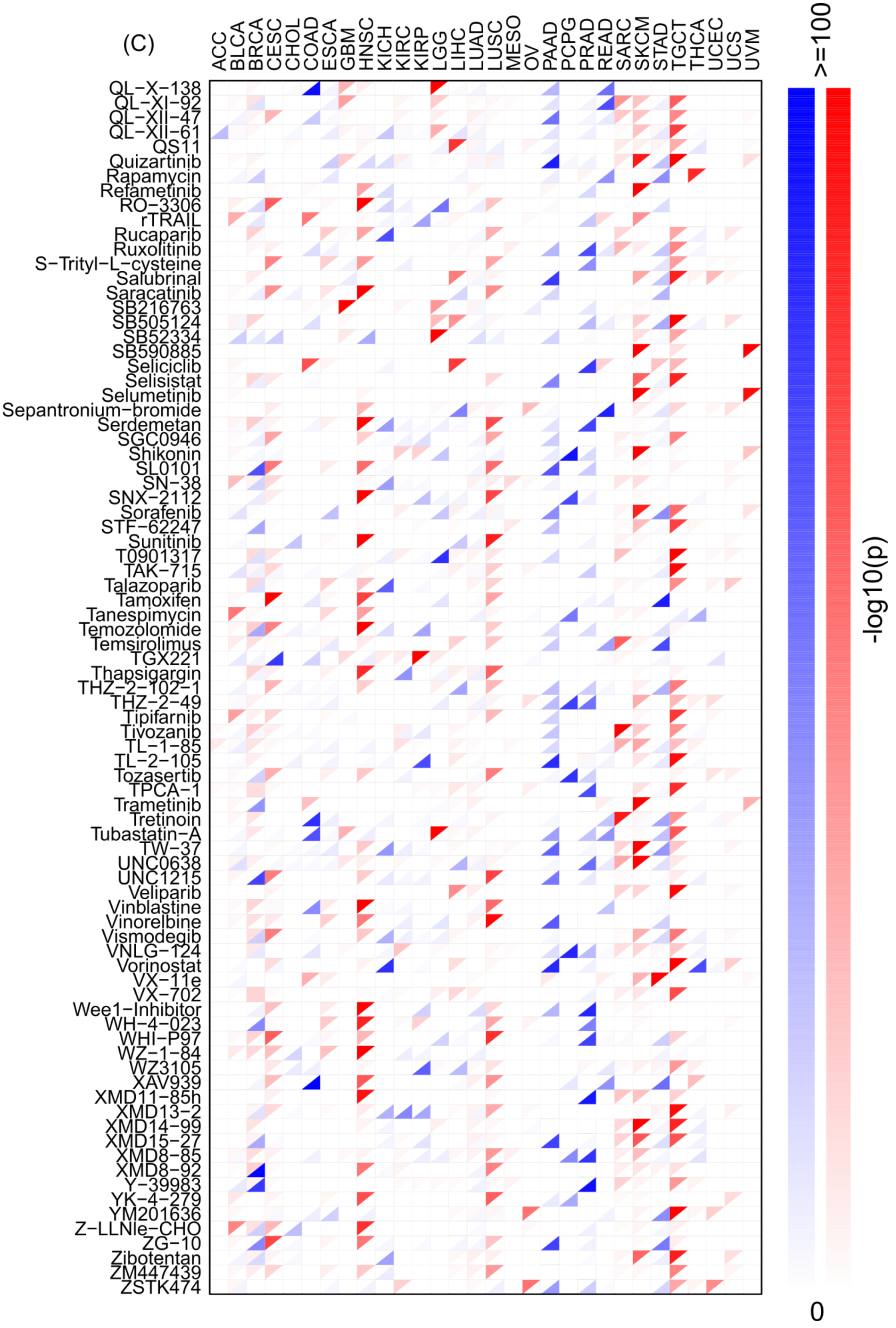
(C)
ACC
BLCA
BRCA
CESC
CHOL
COAD
ESCA
GBM
HNSC
KICH
KIRC
KIRP
LGG
LIHC
LUAD
LUSC
MESO
OV
PAAD
PCPG
PRAD
READ
SARC
SKCM
STAD
TGCT
THCA
UCEC
UCS
UVM
QL−X−138
QL−XI−92
QL−XII−47
QL−XII−61
QS11
Quizartinib
Rapamycin
Refametinib
RO−3306
rTRAIL
Rucaparib
Ruxolitinib
S−Trityl−L−cysteine
Salubrinal
Saracatinib
SB216763
SB505124
SB52334
SB590885
Seliciclib
Selisistat
Selumetinib
Sepantronium−bromide
Serdemetan
SGC0946
Shikonin
SL0101
SN−38
SNX−2112
Sorafenib
STF−62247
Sunitinib
T0901317
TAK−715
Talazoparib
Tamoxifen
Tanespimycin
Temozolomide
Temsirolimus
TGX221
Thapsigargin
THZ−2−102−1
THZ−2−49
Tipifarnib
Tivozanib
TL−1−85
TL−2−105
Tozasertib
TPCA−1
Trametinib
Tretinoin
Tubastatin−A
TW−37
UNC0638
UNC1215
Veliparib
Vinblastine
Vinorelbine
Vismodegib
VNLG−124
Vorinostat
VX−11e
VX−702
Wee1−Inhibitor
WH−4−023
WHI−P97
WZ−1−84
WZ3105
XAV939
XMD11−85h
XMD13−2
XMD14−99
XMD15−27
XMD8−85
XMD8−92
Y−39983
YK−4−279
YM201636
Z−LLNle−CHO
ZG−10
Zibotentan
ZM447439
ZSTK474
>=100
-log10(p)
0

Supplementary Figure 8. Enrichment test results of the sensitive or insensitive samples in each of the 30 cancer types (excluding 3 immune related cancer: DLBC, LAML, and THYM) using 251 GDSC drugs. For each cell, the top left triangle shows the sensitive trend (in red) and the bottom right triangle shows the insensitive trend (in blue), with the color proportional to the *p*-value. The *p*-value was similarly calculated as explained in Figure 3C.

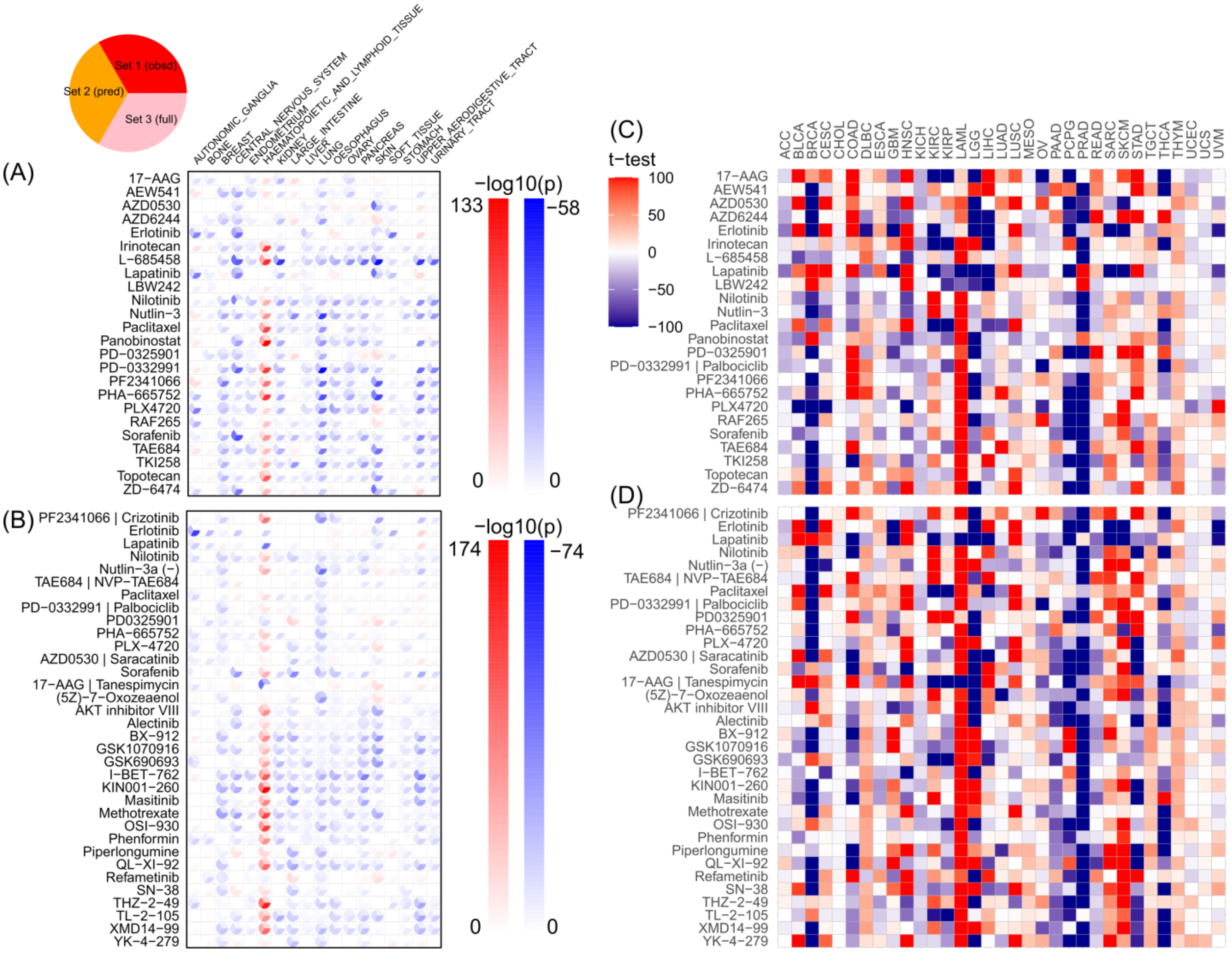

Supplementary Figure 9. Impact of cell lineages or cancer types on drug response. (A) Impact of cell lineages with the 24 CCLE drugs. In each pie, three *p*-values were shown to test the corresponding drug-lineage associations using three sample sets: the observed drug response, the predicted drug response in cell lines with observed data, and the predicted drug response in all 1100 cell lines. Red indicates the enrichment while blue indicates depletion. (B) Association of cancer types with the 24 CCLE drugs based on the predicted drug response. Similarly, red indicates enrichment while blue indicates depletion. (C) Impact of cell lineages using representative drugs in GDSC models. The top 14 drugs were shared with those measured in CCLE. The remaining drugs were those with the strongest variation of the association patterns among the 33 cancer types. (D) Association of cancer types with representative GDSC drugs (same as in C).

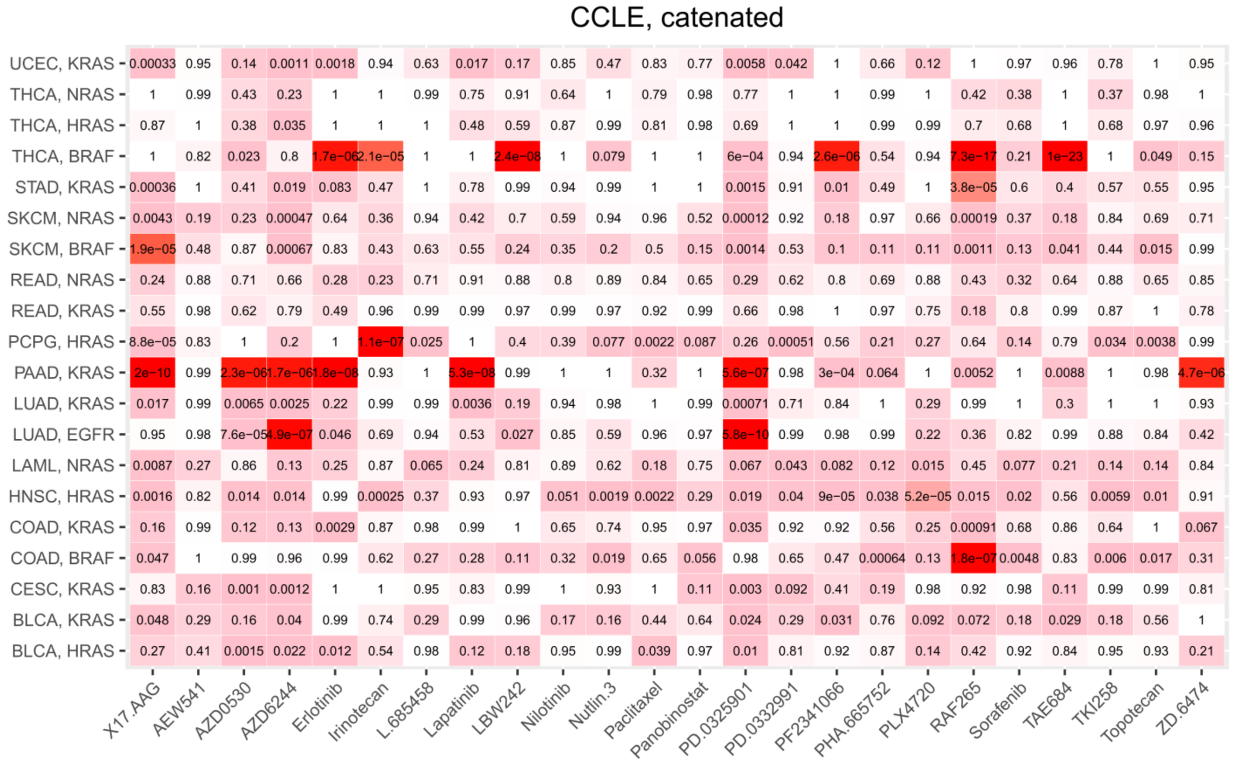

Supplementary Figure 10. Association of *BRAF* (V600E), *EGFR* (positions 719, 746, 858, and 861), and Ras genes (positions 12, 13, and 61) with 24 CCLE drugs. The *p*-value was obtained by a one-sided *t*-test to compare the samples with the corresponding mutations and the samples with pan-negative mutations in all five genes (*BRAF*, *KRAS*, *HRAS*, *NRAS*, and *EGFR*). The alternative hypothesis was that the mutant samples had higher response than the pan-negative samples. The mutant group were required to have more than 10 samples.

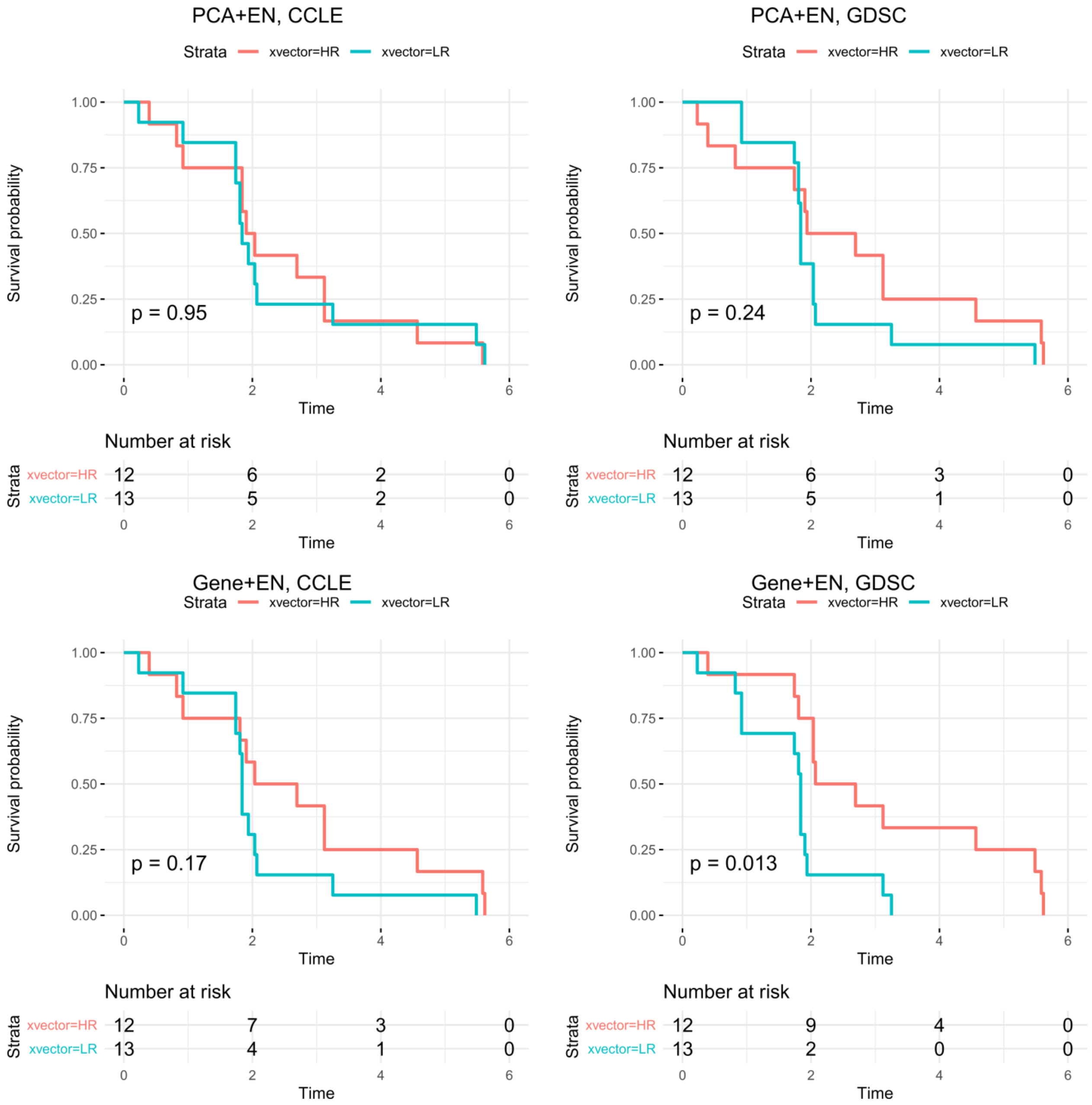

Supplementary Figure 11. Survival analysis results using the PCA+EN or gene+EN models for GSE33072: NSCLC samples were treated with Erlotinib, an EGFR inhibitor. The *p*-values were from a log-rank test comparing two groups of samples defined by the predicted response to Erlotinib (HR: high response, greater than the median; LR: low response).

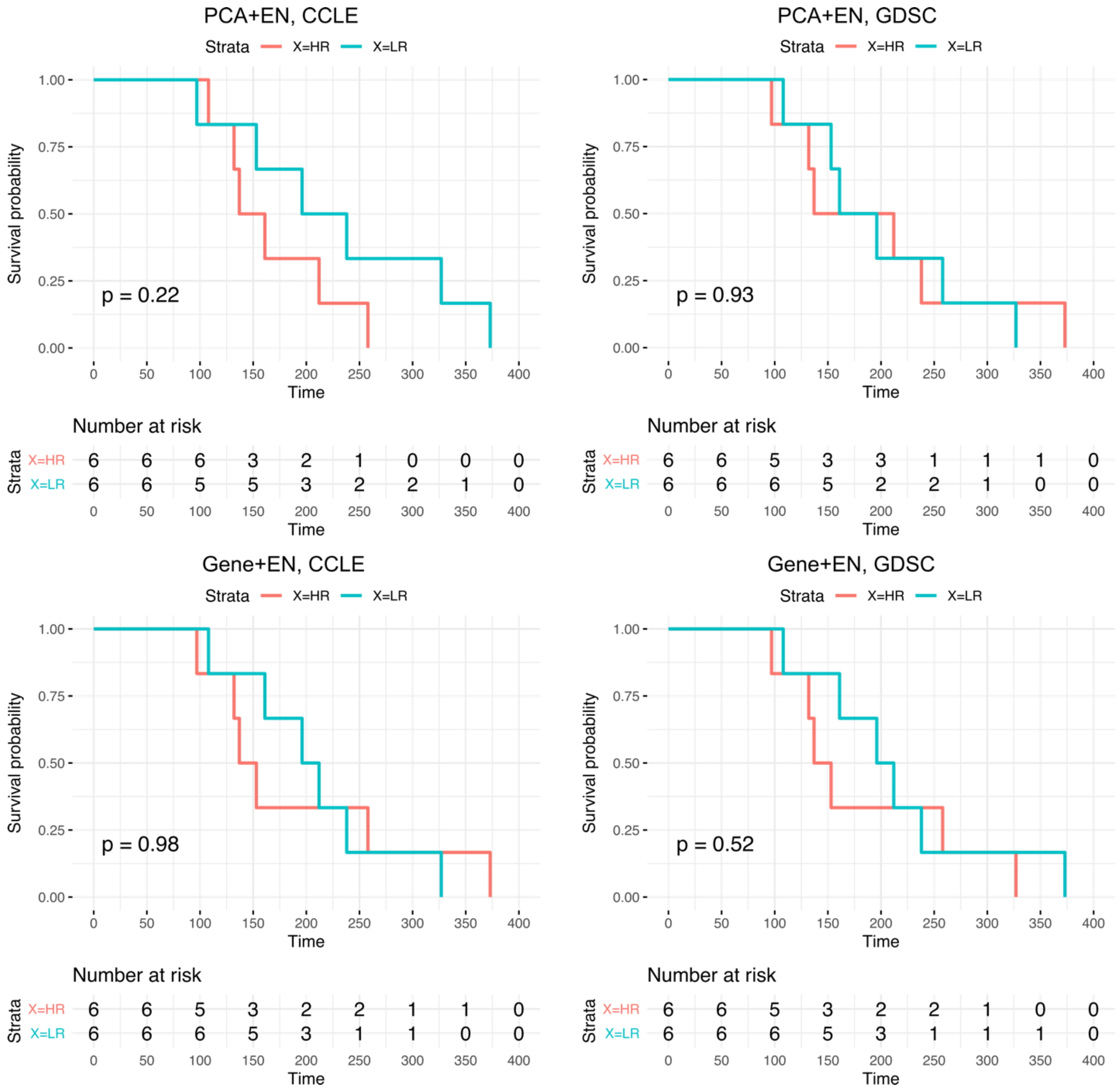

Supplementary Figure 12. Survival analyses using the PCA+EN or gene+EN models for GSE65186 samples who were treated with vemurafenib. We stratified these samples by predicted response to PLX4720, a BRAF inhibitor. HR: high response. LR: low response. The *p*-values were from a log-rank test comparing the HR group and the LR group.

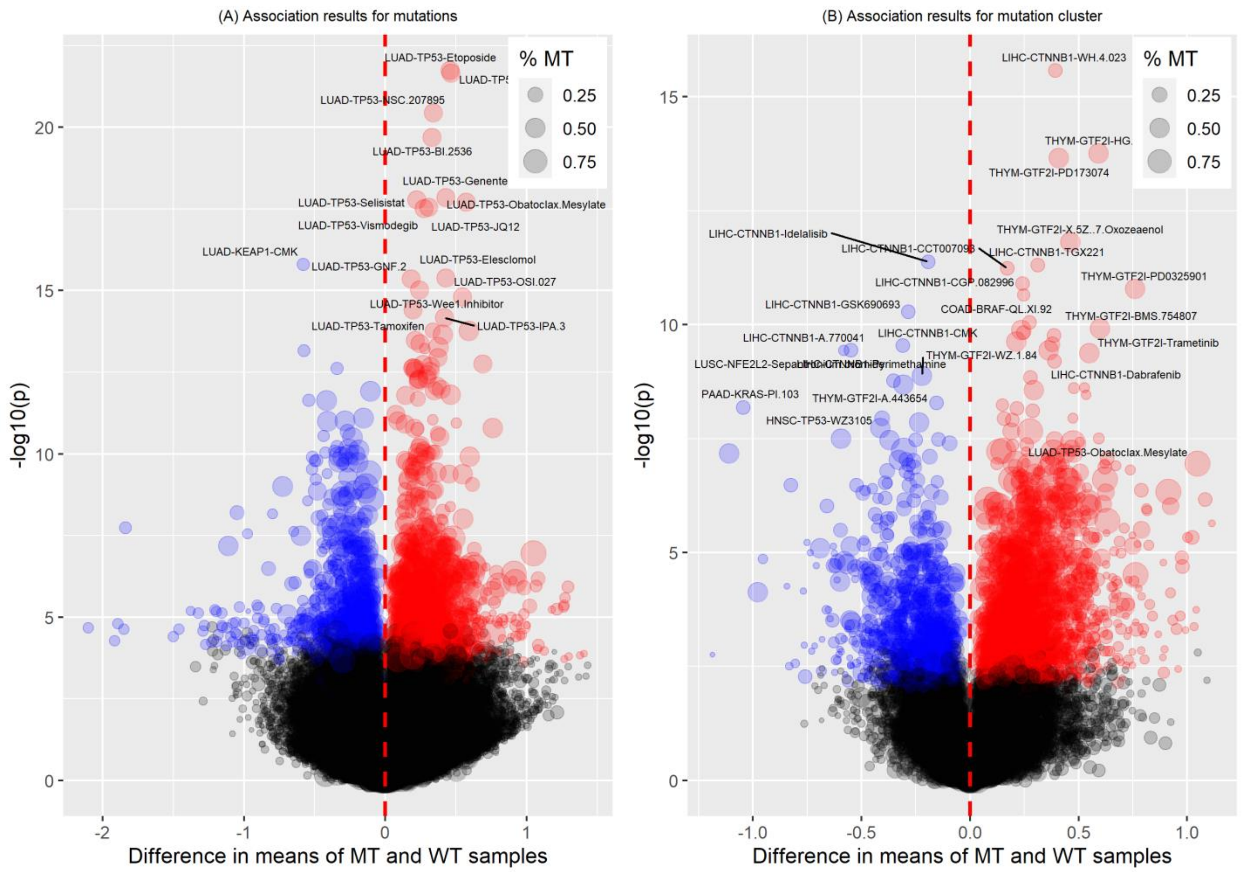

Supplementary Figure 13. Association of somatic mutations with drug response (GDSC). (A) Volcano plot of drug-gene association. Each dot represents the statistics of the association status of a gene in a cancer type with a drug. The *p*-value was obtained from a two-sided Wilcoxon test comparing the predicted response in samples with the mutant gene (i.e., those with deleterious missense SNVs or nonsense SNVs) and samples with the wild type gene. The former group was required to have ≥ 10 samples. X-axis: difference of the average drug response in the samples harboring the mutated gene (MT) from that in the wild type (WT) samples. Y-axis: -log10(p) where the unadjusted *p*-value was used for plotting. Red (difference > 0) and blue (difference < 0) dots indicate significant associations (BH-adjusted $p < 0.05$). The size of the dots is proportional to the percentage of MT samples. (B) Volcano plot of the associations between drugs and mutation clusters in genes. Each dot represents the statistics of the association status of a mutation cluster in a gene with a drug in a cancer type. The *p*-value was obtained from a two-sided Wilcoxon test comparing the predicted response in samples with mutations located in the cluster of the gene and samples with the wild type gene. The former group was required to have ≥10 samples. X-axis: difference of the average drug response in the samples harboring cluster mutations (MT) from that in the wild samples (WT). Y-axis: -log10(p) where the unadjusted *p*-value was used for plotting. Red (difference > 0) and blue (difference < 0) dots indicate significant associations (BH-adjusted $p < 0.05$).
