# Supplementary material for: Deep generative neural network for accurate drug response imputation
Source: Nat Commun. 2021 Mar 19;12:1740. doi: 10.1038/s41467-021-21997-5 (PMC7979803; doi:10.1038/s41467-021-21997-5)
Supplement: Supplementary file 2 — Reporting Summary [file 41467_2021_21997_MOESM2_ESM.pdf]

Corresponding author(s): Zhongming Zhao
Last updated by author(s): Feb 10, 2021

Reporting Summary

Nature Research wishes to improve the reproducibility of the work that we publish. This form provides structure for consistency and transparency in reporting. For further information on Nature Research policies, see our Editorial Policies and the Editorial Policy Checklist.

Statistics

For all statistical analyses, confirm that the following items are present in the figure legend, table legend, main text, or Methods section.

n/a	Confirmed	
☐	☒	The exact sample size (*n*) for each experimental group/condition, given as a discrete number and unit of measurement
☒	☐	A statement on whether measurements were taken from distinct samples or whether the same sample was measured repeatedly
☐	☒	The statistical test(s) used AND whether they are one- or two-sided<br>*Only common tests should be described solely by name; describe more complex techniques in the Methods section.*
☐	☒	A description of all covariates tested
☐	☒	A description of any assumptions or corrections, such as tests of normality and adjustment for multiple comparisons
☐	☒	A full description of the statistical parameters including central tendency (e.g. means) or other basic estimates (e.g. regression coefficient) AND variation (e.g. standard deviation) or associated estimates of uncertainty (e.g. confidence intervals)
☐	☒	For null hypothesis testing, the test statistic (e.g. *F*, *t*, *r*) with confidence intervals, effect sizes, degrees of freedom and *P* value noted<br>*Give P values as exact values whenever suitable.*
☒	☐	For Bayesian analysis, information on the choice of priors and Markov chain Monte Carlo settings
☒	☐	For hierarchical and complex designs, identification of the appropriate level for tests and full reporting of outcomes
☐	☒	Estimates of effect sizes (e.g. Cohen's *d*, Pearson's *r*), indicating how they were calculated

Our web collection on statistics for biologists contains articles on many of the points above.

Software and code

Policy information about availability of computer code

Data collection	We did not collect data for this study. We analyzed publicly available data.
Data analysis	Custom R (version 3.6.3) and python (version 3) scripts are developed and made available at GitHub. All codes are available at GitHub: https://github.com/bsml320/VAEN/. The mutation data of TCGA that we used were downloaded from UCSC Cancer Genome Browser Xena, which alraedy provided PolyPhen results.

For manuscripts utilizing custom algorithms or software that are central to the research but not yet described in published literature, software must be made available to editors and reviewers. We strongly encourage code deposition in a community repository (e.g. GitHub). See the Nature Research guidelines for submitting code & software for further information.

Data

Policy information about availability of data

All manuscripts must include a data availability statement. This statement should provide the following information, where applicable:

- Accession codes, unique identifiers, or web links for publicly available datasets
- A list of figures that have associated raw data
- A description of any restrictions on data availability

The original CCLE (https://portals.broadinstitute.org/ccle), GDSC (https://www.cancerrxgene.org/), and TCGA (https://xenabrowser.net/) data are publicly available datasets. The links for the validation datasets are: GSE33072 [https://www.ncbi.nlm.nih.gov/geo/query/acc.cgi?acc=GSE33072], GSE65185 [https://www.ncbi.nlm.nih.gov/geo/query/acc.cgi?acc=GSE65185], GSE25055 [https://www.ncbi.nlm.nih.gov/geo/query/acc.cgi?acc=GSE25055], GSE32646 [https://www.ncbi.nlm.nih.gov/geo/query/acc.cgi?acc=GSE32646], GSE20194 [https://www.ncbi.nlm.nih.gov/geo/query/acc.cgi?acc=GSE20194], and GSE32989 [https://www.ncbi.nlm.nih.gov/geo/query/acc.cgi?acc=GSE32989]. All results from this study are made available at GitHub (https://github.com/bsml320/VAEN/) or our lab

website (https://bioinfo.uth.edu/VAEN/) due to file size issues, including the latent and weight files from VAE, the predicted drug response for CCLE and GDSC, and the predicted drug response for TCGA based on the CCLE and GDSC models for each drug.

Field-specific reporting

Please select the one below that is the best fit for your research. If you are not sure, read the appropriate sections before making your selection.

☒ Life sciences ☐ Behavioural & social sciences ☐ Ecological, evolutionary & environmental sciences

For a reference copy of the document with all sections, see nature.com/documents/nr-reporting-summary-flat.pdf

Life sciences study design

All studies must disclose on these points even when the disclosure is negative.

Sample size	In CCLE data, we required each cell line origin to have >= 20 cell lines. In the association test between mutations and drug response using TCGA samples, we required the genes qualified for a test to be mutated in >=10 samples.
Data exclusions	Cell lines belonging to under-represented origins were excluded. We consider the "under-represented origins" as those with <20 cell lines. The exclusion criteria was not pre-established.
Replication	We used 6 independent datasets to replicate some of the results. The GEO accession ID of these datasets are provided.
Randomization	To train the Elastic Net models for each drug, we used 5-fold cross-validation. Samples were randomly assigned to each group.
Blinding	We used 5-fold cross-validation to train the Elastic Net models. Investigators were blinded how samples were assigned to each group.

Reporting for specific materials, systems and methods

We require information from authors about some types of materials, experimental systems and methods used in many studies. Here, indicate whether each material, system or method listed is relevant to your study. If you are not sure if a list item applies to your research, read the appropriate section before selecting a response.

Materials & experimental systems

n/a	Involved in the study	
☒	☐	Antibodies
☒	☐	Eukaryotic cell lines
☒	☐	Palaeontology and archaeology
☒	☐	Animals and other organisms
☒	☐	Human research participants
☒	☐	Clinical data
☒	☐	Dual use research of concern

Methods

n/a	Involved in the study	
☒	☐	ChIP-seq
☒	☐	Flow cytometry
☒	☐	MRI-based neuroimaging
